# Identification of putative Type-I sex pheromone biosynthesis-related genes expressed in the female pheromone gland of *Streltzoviella insularis*

**DOI:** 10.1371/journal.pone.0227666

**Published:** 2020-01-16

**Authors:** Yuchao Yang, Jing Tao, Shixiang Zong

**Affiliations:** Beijing Key Laboratory for Forest Pest Control, School of Forestry, Beijing Forestry University, Beijing, China; USDA Agricultural Research Service, UNITED STATES

## Abstract

Species-specific sex pheromones play key roles in moth sexual communication. Although the general pathway of Type-I sex pheromone biosynthesis is well established, only a handful of genes encoding enzymes involved in this pathway have been characterized. *Streltzoviella insularis* is a destructive wood-boring pest of many street trees in China, and the female sex pheromone of this species comprises a blend of (*Z*)-3-tetradecenyl acetate, (*E*)-3-tetradecenyl acetate, and (*Z*)-5-dodecenyl acetate. This organism therefore provides an excellent model for research on the diversity of genes and molecular mechanisms involved in pheromone production. Herein, we assembled the pheromone gland transcriptome of *S*. *insularis* by next-generation sequencing and identified 74 genes encoding candidate key enzymes involved in the fatty acid biosynthesis, β-oxidation, and functional group modification. In addition, tissue expression patterns further showed that an acetyl-CoA carboxylase and two desaturases were highly expressed in the pheromone glands compared with the other tissues, indicating possible roles in *S*. *insularis* sex pheromone biosynthesis. Finally, we proposed putative *S*. *insularis* biosynthetic pathways for sex pheromone components and highlighted candidate genes. Our findings lay a solid foundation for understanding the molecular mechanisms underpinning *S*. *insularis* sex pheromone biosynthesis, and provide potential targets for disrupting chemical communication that could assist the development of novel pest control methods.

## Introduction

Lepidoptera sex pheromones, which are usually secreted by female moths to attract conspecific males, play a key role in sexual communication, and are used as a monitoring and trapping tool in integrated pest management programs [1–3]. In general, moth sex pheromones are composed of two or more components in a unique ratio, and are classified into four types (Type-I, Type-II, Type-III, and Type-0) according to their site of production, chemical structure, and biosynthetic features [4]. Type-I sex pheromones are alcohols and their derivatives (acetates and aldehydes) with long straight chains (C_10_–C_18_) which are used by most moths [1, 5]. Type-II sex pheromones are composed of C_17_–C_23_ hydrocarbons with two or three double bonds at the three, six, or nine positions, or their corresponding epoxide derivatives [1, 5]. Compared with Type-I and Type-II sex pheromones, Type-III sex pheromones with one or more methyl branches possess distinct biosynthetic features, and these components include C_17_–C_23_ saturated and unsaturated hydrocarbons, as well as functionalized hydrocarbons [5]. Type-0 sex pheromones, short-chain secondary alcohols or ketones similar to some general plant volatile compounds, are utilized by the oldest non-ditrysian lineages of Lepidoptera species and are thought to represent the ancestral type of sex pheromone [5–7]. Moth sex pheromones, particularly Type-I, are mainly biosynthesized in and released from the sex pheromone gland (PG) located at the inter-segmental membrane between the eighth and ninth abdominal segments [4, 8].

The general biosynthesis pathway for Type-I sex pheromones in moths is well established; they are synthesized *de novo* through modified fatty acid biosynthesis pathways, and several enzymatic reactions are indispensable, including desaturation, oxidation, reduction, and acetylation [1, 4, 9–12]. All carbon atoms of the fatty acid are derived from acetyl-CoA, acetyl-CoA carboxylase (ACC) converts acetyl-CoA into the fatty acid precursor malonyl-CoA [13], and fatty acid synthetase (FAS) produces palmitic acid (C16) or stearic acid (C18) using acetyl-CoA and malonyl-CoA as substrate and NADPH as reducing agent [14–15]. Double bonds are introduced into the acyl chain at specific positions by desaturases (DESs), of which seven (Δ5 [16], Δ6 [17], Δ9 [18], Δ10 [19], Δ11 [20], Δ12 [11], and Δ14 [21]) have been identified in Lepidoptera species based on signature motifs. For instance, Δ9-desaturases have been divided into two groups: one with a substrate chain length preference of C16 >C18 (NPVE motif), and the other with a substrate chain length preference of C18 >C16 (KPSE motif) [22]. Subsequently, the unsaturated fatty acid is subjected to chain-shortening by β-oxidation, generating sex pheromone precursors of specific chain length [23], and the carbonyl carbon is modified to form an oxygenated functional group, such as an aldehyde, alcohol, or acetate ester, and these modifications involve some key biosynthesis enzymes; fatty acyl-CoA reductase (FAR) converts these acyl chains into fatty alcohols that act as actual sex pheromone components in various moths [24–26], but most fatty alcohols are either oxidized into the corresponding aldehyde by dehydrogenases [27–28] or esterified to form acetate esters by acetyltransferase (ATF) [29–31], resulting in the final functional groups.

*Streltzoviella insularis* (Staudinger) (Lepidoptera: Cossidae) is a destructive wood-boring pest and occurs in many provinces and cities in China. It mainly damages various street trees, such as *Fraxinus americana*, *Ginkgo biloba*, *Sophora* spp., and *Ulmus* spp., causing great economic losses to urban forestry [32–34]. The female sex pheromone of *S*. *insularis* is a blend of (*Z*)-3-tetradecenyl acetate (Z3-14:OAc), (*E*)-3-tetradecenyl acetate (E3-14:OAc), and (*Z*)-5-dodecenyl acetate (Z5-12:OAc) [35–36], and these acetate esters are typical of Type-I sex pheromones. These different components indicate the involvement of different desaturases, β-oxidases, and reductases during sex pheromone production. Characterization of the genes encoding putative key enzymes involved in this process may not only help to elucidate the sex pheromone biosynthesis pathway in *S*. *insularis*, but may also provide potential targets for disrupting sexual communication for pest control purposes. Hence, in the present study, we first constructed a transcriptome library of *S*. *insularis* PGs and identified a series of genes that might be involved in sex pheromone biosynthesis. Tissue expression patterns and phylogenetic analysis were performed to postulate the potential functions of the identified genes. Based on the results, we propose putative biosynthetic pathways for the sex pheromone components in *S*. *insularis*.

## Materials and methods

### Ethics statement

*S*. *insularis* is not on the List of Endangered and Protected Animals in China. The Beijing Municipal Bureau of Landscape and Forestry issued a permit for field collection.

### Sample collection

*S*. *insularis* individuals were collected from *Fraxinus americana* at Beijing Forestry University North Road, Haidian District, Beijing, China, in May 2017. Damaged trunks were chopped down, taken to the laboratory, and larvae inside trunks were fed on the phloem and xylem of the host under natural environmental conditions. Adult moths were sexed after emergence according to the genitalia. The pheromone gland and associated ovipositor valves, as well as parts of the terminal abdominal segments (together abbreviated as PG) were dissected from 1-day-old and 2-day-old female adults during the scotophase, which is reported to be the calling period of this moth [35, 37]. In addition, antennae and legs were also collected at the same time, immediately placed in RNAlater (Ambion, Austin, TX, USA), and stored at -80°C.

### RNA extraction

Total RNA was extracted from 15 PGs (seven PGs from 1-day-old females and eight PGs from 2-day-old females) using TRIzol reagent (Invitrogen, Carlsbad, CA, USA) following the manufacturer’s instructions, with three biological replicates. RNA purity was evaluated with a NanoDrop 2000 instrument (Thermo, Waltham, MA, USA), and RNA concentration was measured using a Qubit RNA Assay Kit and a Qubit 2.0 Fluorimeter (Life Technologies, CA, USA). RNA integrity was determined by an Agilent Bioanalyzer 2100 system (Agilent Technologies, CA, USA), and RNA degradation and contamination were monitored by 1% agarose gels to ensure the quality of the RNA samples for subsequent transcriptome sequencing.

### cDNA library construction and Illumina sequencing

cDNA library construction and Illumina sequencing of samples were performed at Shanghai Majorbio Bio-pharm Technology Co., Ltd. (Shanghai, China). According to the TruSeq RNA Sample Preparation Guide V2 (Illumina), mRNA was purified from total RNA using Oligo (dT) magnetic beads, then fragmented by adding fragmentation buffer. Random hexamer primers were used to synthesize first-strand cDNA, followed by synthesis of the second strand using dNTPs, RNaseH, and DNA polymerase I. All remaining overhangs were converted into blunt ends via polymerase. After end-repair, poly-A tailing, and ligation of adapters, 150–200 bp cDNA fragments were purified using an AMPure XP system (Beckman Coulter, Beverly, MA, USA), and 3μl USER Enzyme (NEB, USA) was incubated with size-selected, adaptor-ligated cDNA at 37°C for 15 min followed by incubation at 95°C for 5 min, prior to PCR amplification. PCR products were purified using an AMPure XP system, and library quality was assessed on the Agilent Bioanalyzer 2100 system. Finally, *S*. *insularis* cDNA libraries were sequenced on an Illumina Hiseq 4000 platform, and paired-end reads were generated.

### Sequence assembly and functional annotation

To obtain the clean reads, the raw reads were processed to remove low-quality reads and adapter sequences. Then, GC Content, Q20 and Q30 were used to assess the sequencing quality. The qualified reads assembly was carried out with the short reads assembling program-Trinity [38]. The largest alternative splicing variants in the Trinity results were called unigenes. The annotation of unigenes was performed by the National Center for Biotechnology Information (NCBI) BLASTx searches against the non-redundant (Nr) protein database, with a cut-off E-value of 10^−5^. Unigenes were also annotated using other protein databases including Gene Ontology (GO) [39], Clusters of Orthologous Groups of proteins (COG) [40], and Kyoto Encyclopedia of Genes and Genomes (KEGG) [41]. The longest open reading frame (ORF) for each unigene was determined by the NCBI ORF Finder tool (http://www.ncbi.nlm.nih.gov/gorf/gorf.html). Fragments per kilobase of exon per million mapped reads (FPKM) values were calculated by RSEM (RNA-Seq by Expectation-Maximization) with default parameters represented gene expression in *S*. *insularis* PG tissue [42].

### Identification of putative genes involved in sex pheromone biosynthesis

Putative unigenes involved in sex pheromone biosynthesis of *S*. *insularis* were confirmed by analysis with the BLASTx program. All candidate pheromone biosynthesis-activating neuropeptide receptor (PBANR), acetyl-CoA carboxylase (ACC), fatty acid synthase (FAS), desaturase (DES), acyl-CoA oxidase (ACO), acyl-CoA dehydrogenase (ACD), enoyl-CoA hydratase (ECH), L-3-hydroxyacyl-CoA dehydrogenase (HCD), 3-ketoacyl-CoA thiolase (KAT), fatty acyl-CoA reductase (FAR), alcohol dehydrogenase (AD), aldehyde reductase (AR) and acetyltransferase (ATF) genes were manually checked by tBLASTn in NCBI online.

### Sequence and phylogenetic analyses

Amino acid sequences of candidate desaturases were aligned with those of other insect species using ClustalW by MEGA (Version 5.0) [43]. Phylogenetic tree construction was performed using the neighbor-joining method as implemented in MEGA (Version 5.0) with a *p*-distance model and pairwise deletion of gaps. Bootstrap support of tree branches was assessed by re-sampling amino acid positions 1000 times [44]. Phylogenetic trees were colored and arranged using FigTree (Version 1.4.2) [45].

### Expression analysis by quantitative real-time PCR (RT-qPCR)

Expression patterns of putative ACC and DES genes in different tissues (antennae, legs, and PGs) were analyzed by RT-qPCR using a Bio-Rad CFX96 PCR System (Hercules, CA, USA). Total RNA was extracted from 25 antennae, 10 legs, and 15 PGs of female moths following the method described above, and transcribed into cDNA using a PrimeScript RT Reagent Kit with gDNA Eraser (No. RR047A; TaKaRa, Shiga, Japan). Gene-specific primers were designed using Primer 3 Plus (http://www.bioinformatics.nl/cgi-bin/primer3plus/primer3plus.cgi) and are listed in S1 Table. The *S*. *insularis* actin gene served as an internal reference gene. Each RT-qPCR mixture was composed of 12.5 μl of TB Green Premix Ex Taq II (Tli RNaseH Plus; No. RR820A; TaKaRa), 1 μl of forward primer (10 μM), 1 μl of reverse primer (10 μM), 2 μl of cDNA, and 8.5 μl of sterilized H_2_O. RT-qPCR cycling parameters were as follows: 95°C for 30 s, followed by 40 cycles at 95°C for 5 s and 60°C for 30 s, and 65°C to 95°C in increments of 0.5°C for 5 s to generate melting curves. To check reproducibility, each reaction for each tissue was performed with three biological replicates and three technical replicates. Negative controls without template were included in each experiment. Relative expression levels were calculated according to the comparative 2^-ΔΔCt^ method (the amplification efficiency was close to 100% for 12 genes) [46]. Leg samples were used for calibration, and actin was used for calculating and normalizing target gene expression, and correcting for sample to sample variation. Data in the form of means ± standard error (SE) from different samples were subjected to one-way nested analysis of variance, followed by Tukey’s honestly significant difference tests, implemented in SPSS Statistics 22.0 (IBM, Chicago, IL, USA).

## Results and discussion

### Illumina sequencing and unigene assembly

We constructed cDNA libraries utilizing mRNAs from *S*. *insularis* PG tissue samples as template with an Illumina Hiseq 4000 platform, and included three biological replicates. A total of 63,881,910, 54,395,274, and 58,219,720 raw reads were obtained from each library. After removing low-quality reads and adaptors, we finally acquired 60,708,992, 51,561,536, and 55,208,486 clean reads, respectively (Table 1). Subsequently, assembly of all clean reads together resulted in 30,307 unigenes with an N50 value of 2072 bp, an average length of 1385 bp, and a longest length of 26,771 bp. Raw reads have been deposited in the NCBI SRA database under accession number SRP179142.

**Table 1:** Summary of sequencing results.

	Raw data			Clean data		
	Repeat 1	Repeat 2	Repeat 3	Repeat 1	Repeat 2	Repeat 3
**Read number**	63,881,910	54,395,274	58,219,720	60,708,992	51,561,536	55,208,486
**Base number**	9,646,168,410	8,213,686,374	8,791,177,720	9,000,405,945	7,640,975,358	8,189,668,630
**Q20 (%)**	97.11	96.94	97.03	98.27	98.18	98.24
**Q30 (%)**	93.08	92.73	92.98	94.92	94.71	94.92
**GC (%)**	46.69	47.02	44.4	46.57	46.87	44.26

### Homology searching and functional annotation

**Fig 1:**
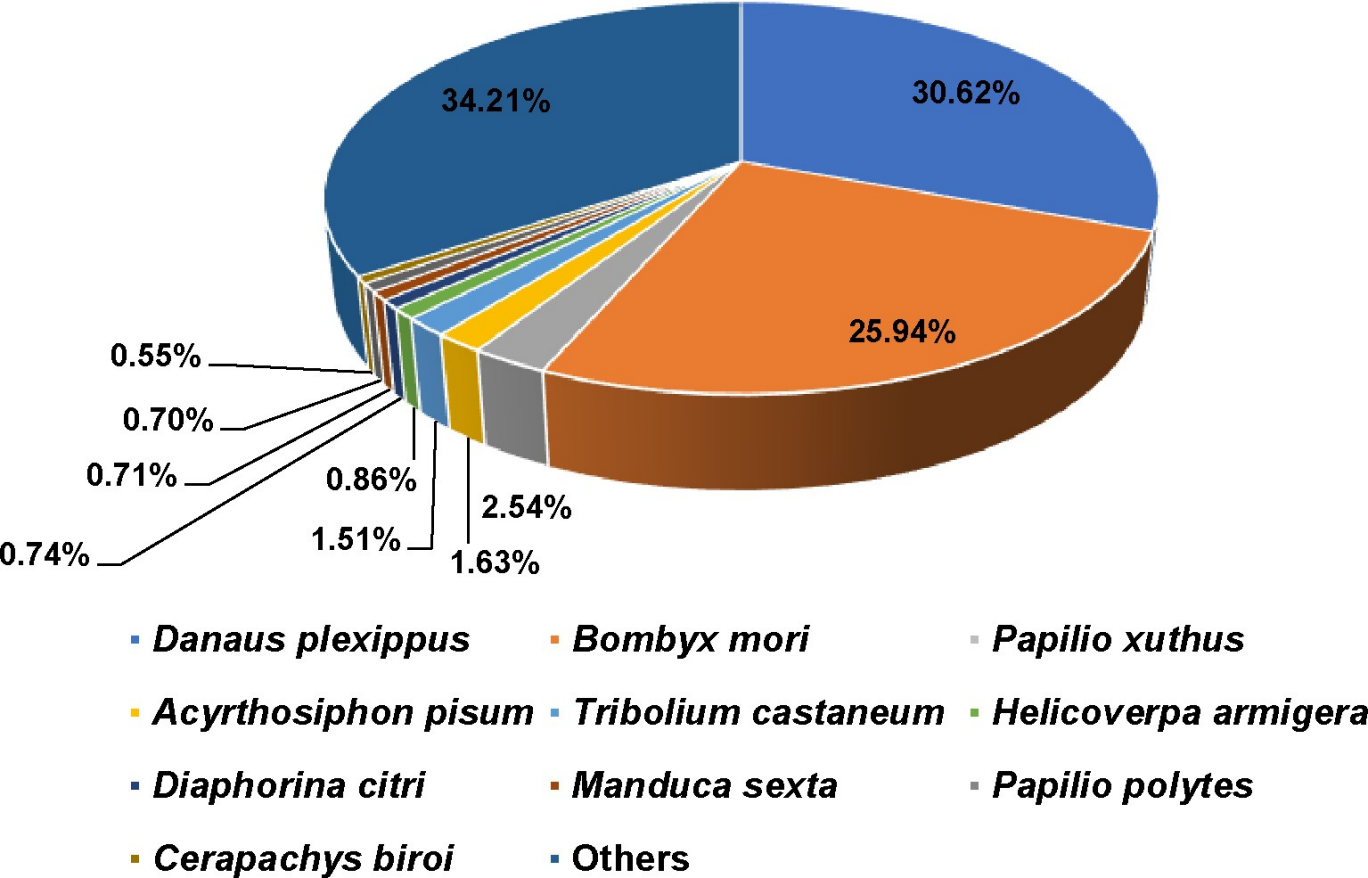
Species distribution based on homology searches of *S*. *insularis* unigenes against the NCBI Nr protein database.

Among the 30,307 unigenes, 16,304 (53.80%) were successfully matched using the BLASTx homology search (cut-off E-value of 10^−5^) to entries in the NCBI Nr protein database. The best matches were obtained for *Danaus plexippus* sequences (30.62%), followed by *Bombyx mori* (25.94%), *Papilio xuthus* (2.54%), and *Acyrthosiphon pisum* (1.63%), as shown in Fig 1.

**Fig 2:**
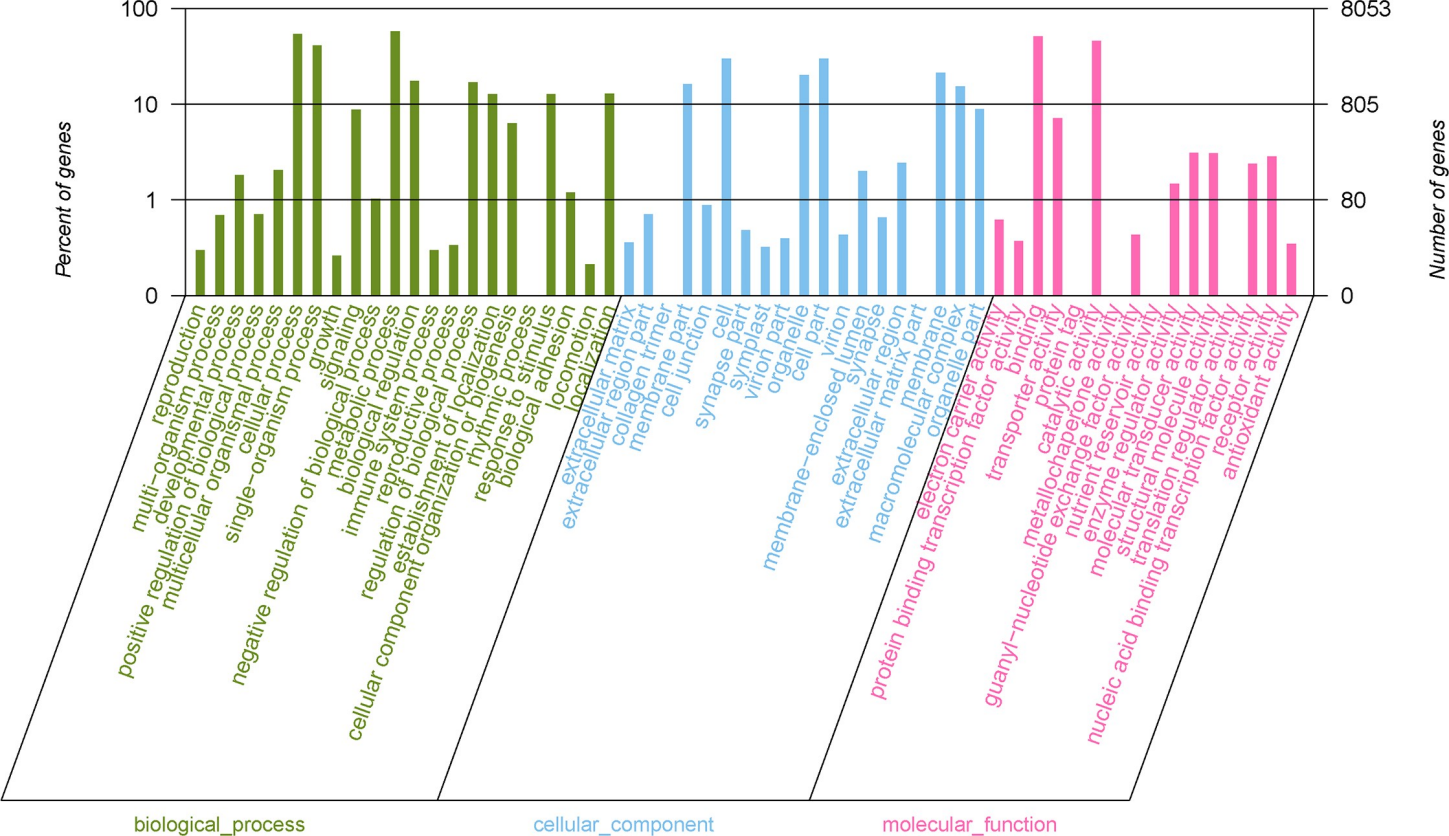
GO classification of *S*. *insularis* unigenes.

**Fig 3:**
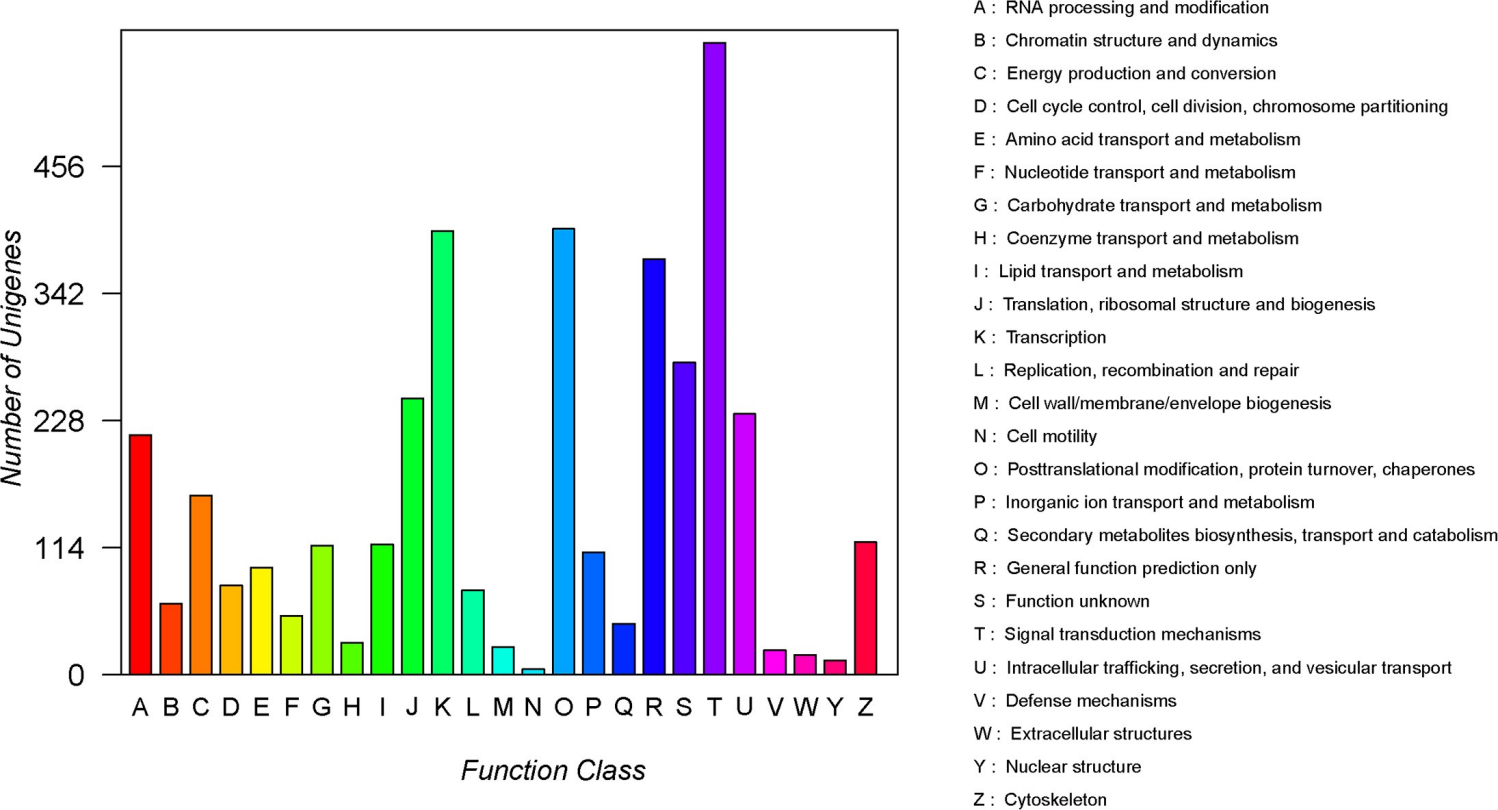
COG classification of *S*. *insularis* unigenes.

**Fig 4:**
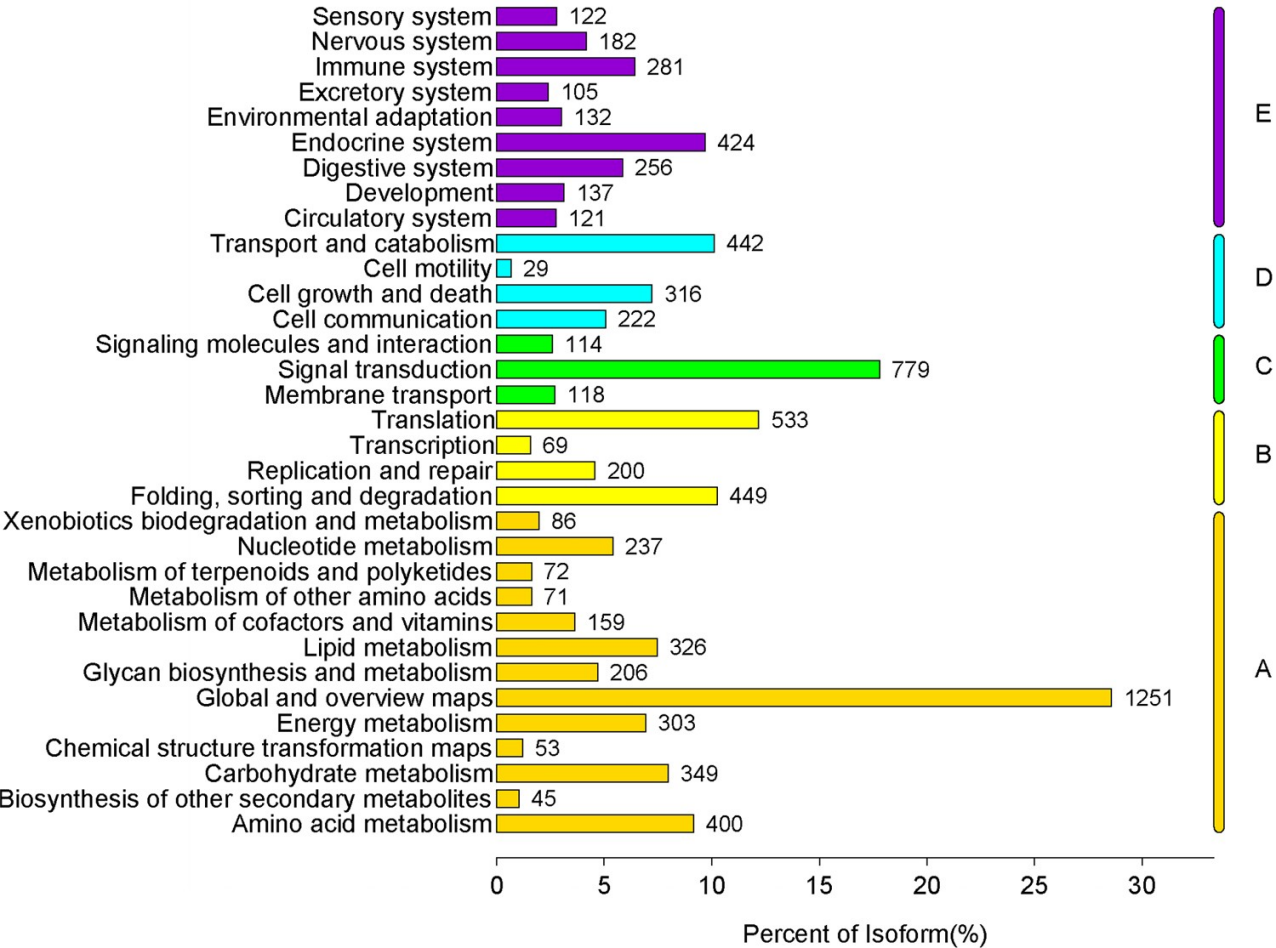
KEGG classification of *S*. *insularis* unigenes.

GO annotation was used to classify the unigenes into three functional groups (molecular function, cellular component, and biological process) according to the GO categories. Of 30,307 unigenes identified in *S*. *insularis*, 8053 (26.57%) were annotated. As shown in Fig 2, 20,072 unigenes were assigned to the ‘molecular function’ category, and ‘binding’ (4141 unigenes, 43.14%) and ‘catalytic activity’ (3695 unigenes, 38.49%) were the most highly represented terms in this category. A total of 12,115 unigenes were assigned to GO terms in the ‘cellular component’ category, and ‘cell part’ (2409 unigenes, 19.88%) and ‘cell’ (2409 unigenes, 19.88%) were the most abundant terms. A further 20,072 unigenes were assigned to GO terms in the ‘biological process’ category, and the main terms were ‘cellular process’ (4329 unigenes, 21.56%) and ‘single-organism process’ (3326 unigenes, 16.57%). In addition, all unigenes were searched against the COG database for functional prediction and classification, and the results showed that 3865 unigenes (12.75%) could be assigned to 25 specific categories (Fig 3); ‘signal transduction mechanisms’ (567 unigenes, 14.67%) was the largest group, and ‘cell motility’ (5 unigenes, 0.13%) was the smallest group. Furthermore, KEGG annotation was used to divide unigenes into five KEGG pathways (cellular processes, environmental information processing, genetic information processing, metabolism, and organismal systems; Fig 4). Most unigenes were assigned to the ‘processes’ branch, and ‘global and overview maps’ (1251 unigenes, 28.07%) was the most highly represented term.

### Pheromone biosynthesis-activating neuropeptide receptor (PBANR)

The biosynthesis of Type-I sex pheromones in female moths has been shown to be regulated by a C-terminally amidated 33 amino acid neuropeptide termed PBAN that is released from the subesophageal ganglion in the brain and transported through the hemolymph to the PG [47–48]. The binding of PBAN to its receptor in the PG cell membrane will induce the opening of Ca^2+^ channels causing the influx of extracellular Ca^2+^, which then initiates sex pheromone production [49–50]. PBANR, a G protein-coupled receptor (GPCR), was first cloned from the PG of *Helicoverpa zea* [51]. PBANR has since been identified in *Bombyx mori* [52] and other Lepidoptera species [49, 53]. PBANRs exist as PBANR multiple isoforms (PBANR-As, -A, -B, and -C) based on alternative splicing of the C-terminus [54]. The various isoforms play different functional roles in the ligand-induced internalization [55], a phase of GPCR feedback regulation and desensitization in diverse moth species [56–57]. Herein, we identified a single PBANR in the *S*. *insularis* PG transcriptome that is 84% identical to *Mamestra brassicae* PBANR isoform B (ARO85772.1) and is very low in abundance (0.56 FPKM; Table 2 and S1 Text). The number of PBANR-encoding genes in the *S*. *insularis* PG was in accordance with *Plutella xylostella* [25], *Agrotis segetum* [58], and *Agrotis ipsilon* [59]. In addition, previous studies identified three isoforms of PBANR in *Ostrinia nubilalis* [60] and *Mamestra brassicae* [61]. However, we did not discover other isoforms of PBANR in our transcriptomic data, which may be explained by lower expression levels in *S*. *insularis*.

**Table 2:** Putative sex pheromone biosynthesis-related genes identified in the *S*. *insularis* pheromone gland transcriptome.

Name	Gene length (bp)	ORF length (bp)	Intact ORF	FPKM value	Best BLASTX match					
					Function	ACC number	Species	Score	E-value	Identity
**PBANR**										
SinsPBANR	1538	1224	Yes	0.56	pheromone biosynthesis activating neuropeptide receptor isoform B	ARO85772.1	*Mamestra brassicae*	726	0	84%
**ACC**										
SinsACC1	723	399	No	3.60	PREDICTED: acetyl-CoA carboxylase	XP_013185423.1	*Amyelois transitella*	164	2E-42	63%
SinsACC2	7616	7101	Yes	28.33	PREDICTED: acetyl-CoA carboxylase isoform X3	XP_013146614.1	*Papilio polytes*	8781	0	90%
**FAS**										
SinsFAS1	312	273	No	0.28	fatty acid synthase	BAM19658.1	*Papilio xuthus*	153	3E-42	88%
SinsFAS2	8170	7173	Yes	90.41	fatty acid synthase	AGR49310.1	*Agrotis ipsilon*	3623	0	81%
SinsFAS3	301	87	No	1.00	PREDICTED: fatty acid synthase	XP_013141731.1	*Papilio polytes*	177	2E-49	85%
SinsFAS4	459	441	No	0.42	fatty acid synthase 1	AKD01760.1	*Helicoverpa assulta*	209	5E-65	62%
SinsFAS5	310	135	No	0.59	fatty acid synthase-like	XP_021208123.1	*Bombyx mori*	164	4E-47	69%
**DES**										
SinsDES1	244	225	Yes	1.81	PREDICTED: acyl-CoA Delta(11) desaturase-like	XP_011561954.1	*Plutella xylostella*	134	2E-36	77%
SinsDES2	449	267	Yes	1.15	acyl-CoA Delta(11) desaturase-like	XP_026752209.1	*Galleria mellonella*	168	4E-48	80%
SinsDES3	864	825	No	0.71	acyl-CoA delta-11 desaturase	AAL16642.1	*Argyrotaenia velutinana*	394	5E-135	63%
SinsDES4	350	228	No	1.20	stearoyl-CoA desaturase 5-like	XP_026757907.1	*Galleria mellonella*	171	2E-49	75%
SinsDES5	305	192	No	0.86	stearoyl-CoA desaturase 5-like	XP_021195328.1	*Helicoverpa armigera*	186	5E-56	80%
SinsDES6	1278	1002	Yes	65.89	desaturase	ARD71185.1	*Spodoptera exigua*	496	1E-172	70%
SinsDES7	1200	996	Yes	4.82	acyl-CoA Delta(11) desaturase-like	XP_028166624.1	*Ostrinia furnacalis*	531	0	78%
SinsDES8	2500	1032	Yes	361.30	acyl-CoA Delta(11) desaturase	XP_028982113.1	*Diachasma alloeum*	345	6E-108	52%
SinsDES9	2947	1143	Yes	5.42	desaturase	AAQ74260.1	*Spodoptera littoralis*	590	0	74%
SinsDES10	7148	1962	Yes	3.36	acyl-CoA-delta9-3a-desaturase	ABX71810.1	*Dendrolimus punctatus*	628	0	87%
SinsDES11	911	393	Yes	1.15	putative C-5 sterol desaturase	KPJ05936.1	*Papilio machaon*	395	4E-134	81%
SinsDES12	483	111	Yes	1.47	fatty acyl desaturase	AHW98356.1	*Cydia pomonella*	98.6	2E-21	77%
SinsDES13	1476	984	Yes	22.89	desaturase	AIM40219.1	*Cydia pomonella*	581	0	85%
SinsDES14	1435	1128	Yes	0.41	desaturase	AIM40222.1	*Cydia pomonella*	638	0	80%
SinsDES15	1563	966	Yes	86.74	sphingolipid delta(4)-desaturase DES1	XP_004930794.1	*Bombyx mori*	612	0	89%
SinsDES16	1484	1017	Yes	1.66	desaturase	ARD71181.1	*Spodoptera exigua*	515	2E-179	72%
SinsDES17	2225	1062	Yes	426.07	acyl-CoA Delta(11) desaturase-like isoform X1	XP_021183600.1	*Helicoverpa armigera*	624	0	82%
**ACO**										
SinsACO1	405	363	No	1.10	probable peroxisomal acyl-coenzyme A oxidase 1	XP_026758799.1	*Galleria mellonella*	215	1E-63	73%
SinsACO2	2480	2013	Yes	42.90	PREDICTED: probable peroxisomal acyl-coenzyme A oxidase 1	XP_013188704.1	*Amyelois transitella*	1166	0	85%
SinsACO3	2792	2070	Yes	1.92	peroxisomal acyl-coenzyme A oxidase 3	XP_022819471.1	*Spodoptera litura*	1181	0	80%
SinsACO4	3173	2097	Yes	11.84	peroxisomal acyl-CoA oxidase 3	AID66678.1	*Agrotis segetum*	1165	0	77%
SinsACO5	375	189	No	0.57	PREDICTED: probable peroxisomal acyl-coenzyme A oxidase 1	XP_014367103.1	*Papilio machaon*	236	5E-77	89%
SinsACO6	2104	1899	No	26.85	probable peroxisomal acyl-coenzyme A oxidase 1 isoform X1	XP_022821900.1	*Spodoptera litura*	964	0	73%
SinsACO7	1919	1893	No	9.16	PREDICTED: probable peroxisomal acyl-coenzyme A oxidase 1	XP_013149571.1	*Papilio polytes*	992	0	75%
SinsACO8	279	243	No	0.00	probable peroxisomal acyl-coenzyme A oxidase 1	XP_021195539.1	*Helicoverpa armigera*	181	5E-52	90%
**ACD**										
SinsACD1	1214	768	Yes	19.22	3-hydroxyacyl-CoA dehydrogenase type-2	XP_026727946.1	*Trichoplusia ni*	464	1E-161	89%
SinsACD2	3886	1902	Yes	214.36	very long-chain-specific acyl-CoA dehydrogenase, mitochondrial isoform X1	XP_026737732.1	*Trichoplusia ni*	944	0	80%
SinsACD3	1056	774	Yes	3.34	3-hydroxyacyl-CoA dehydrogenase type-2-like isoform X1	XP_026761478.1	*Galleria mellonella*	429	7E-149	79%
SinsACD4	1252	933	Yes	105.77	hydroxyacyl-coenzyme A dehydrogenase, mitochondrial-like	XP_022822785.1	*Spodoptera litura*	581	0	89%
SinsACD5	1547	1266	Yes	145.78	short/branched-chain-specific acyl-CoA dehydrogenase, mitochondrial	XP_023946257.1	*Bicyclus anynana*	808	0	92%
SinsACD6	2320	1830	Yes	11.66	PREDICTED: acyl-CoA dehydrogenase family member 9, mitochondrial	XP_013192619.1	*Amyelois transitella*	902	0	69%
SinsACD7	3306	1236	Yes	12.58	short-chain-specific acyl-CoA dehydrogenase, mitochondrial-like isoform X1	XP_028162581.1	*Ostrinia furnacalis*	697	0	81%
SinsACD8	2324	1275	Yes	210.50	probable medium-chain-specific acyl-CoA dehydrogenase, mitochondrial	NP_001298861.1	*Papilio xuthus*	712	0	84%
SinsACD9	4692	1224	Yes	17.89	short-chain-specific acyl-CoA dehydrogenase, mitochondrial	XP_026489065.1	*Vanessa tameamea*	709	0	89%
**ECH**										
SinsECH1	1207	990	Yes	10.81	PREDICTED: probable enoyl-CoA hydratase	XP_013137975.1	*Papilio polytes*	484	2E-168	82%
SinsECH2	1321	303	Yes	2.54	enoyl-CoA hydratase domain-containing protein 3, mitochondrial isoform X2	XP_022822616.1	*Spodoptera litura*	393	3E-85	82%
SinsECH3	1439	894	Yes	13.68	enoyl-CoA hydratase domain-containing protein 2, mitochondrial	XP_028167557.1	*Ostrinia furnacalis*	445	1E-152	79%
**HAD**										
SinsHAD1	1214	768	Yes	19.22	3-hydroxyacyl-CoA dehydrogenase type-2	XP_026727946.1	*Trichoplusia ni*	464	1E-161	89%
SinsHAD2	1056	774	Yes	3.34	3-hydroxyacyl-CoA dehydrogenase type-2-like isoform X1	XP_026761478.1	*Galleria mellonella*	429	7E-149	79%
SinsHAD3	1252	933	Yes	105.77	hydroxyacyl-CoA dehydrogenase	AID66694.1	*Agrotis segetum*	575	0	87%
**KAT**										
SinsKAT1	1395	1194	Yes	10.21	3-ketoacyl-CoA thiolase, mitochondrial-like	XP_028176321.1	*Ostrinia furnacalis*	491	5E-169	63%
**FAR**										
SinsFAR1	1805	1545	No	5.57	PREDICTED: fatty acyl-CoA reductase 1-like	XP_013185409.1	*Amyelois transitella*	761	0	71%
SinsFAR2	1867	1692	No	1.62	fatty acyl reductase 5	ATJ44463.1	*Helicoverpa armigera*	816	0	73%
SinsFAR3	2335	1875	Yes	33.44	fatty acyl-CoA reductase 2	ADI82775.1	*Ostrinia nubilalis*	992	0	80%
SinsFAR4	457	354	No	0.85	fatty acyl reductase	ARD71192.1	*Spodoptera exigua*	193	3E-56	77%
SinsFAR5	967	723	No	0.56	fatty acyl-CoA reductase 1	XP_021197389.1	*Helicoverpa armigera*	360	4E-118	57%
SinsFAR6	2395	1494	Yes	476.06	fatty acyl reductase	AID66655.1	*Agrotis segetum*	441	1E-143	46%
SinsFAR7	2040	1575	Yes	15.48	putative fatty acyl-CoA reductase CG5065	XP_004925992.1	*Bombyx mori*	900	0	84%
SinsFAR8	2910	1578	Yes	0.60	putative fatty acyl-CoA reductase CG5065	XP_026483533.1	*Vanessa tameamea*	1019	0	92%
SinsFAR9	1820	1605	No	9.83	fatty acyl reductase	ARD71186.1	*Spodoptera exigua*	726	0	73%
SinsFAR10	1807	1560	Yes	2.70	fatty acyl-CoA reductase 1	XP_021197389.1	*Helicoverpa armigera*	783	0	73%
SinsFAR11	1875	1500	Yes	65.72	putative fatty acyl-CoA reductase CG5065	XP_028038252.1	*Bombyx mandarina*	792	0	73%
SinsFAR12	2448	1590	Yes	35.68	putative fatty acyl-CoA reductase CG5065	XP_022835056.1	*Spodoptera litura*	635	0	64%
SinsFAR13	4732	1533	Yes	24.19	putative fatty acyl-CoA reductase CG8306	XP_004930778.1	*Bombyx mori*	855	0	79%
**AD**										
SinsAD1	1221	975	Yes	28.73	alcohol dehydrogenase	BAR64763.1	*Ostrinia furnacalis*	529	0	80%
SinsAD2	1746	813	Yes	13.93	alcohol dehydrogenase AD1	AII21999.1	*Sesamia inferens*	360	4E-118	66%
SinsAD3	2923	1131	Yes	35.49	alcohol dehydrogenase class-3	XP_021189392.1	*Helicoverpa armigera*	658	0	94%
SinsAD4	1395	1059	Yes	7.76	alcohol dehydrogenase	BAR64764.1	*Ostrinia furnacalis*	579	0	80%
SinsAD5	1209	750	Yes	37.20	alcohol dehydrogenase AD2	AKQ06148.1	*Cydia pomonella*	327	7E-108	71%
**AR**										
SinsAR1	853	807	No	6.56	aldo-keto reductase AKR2E4-like	XP_028160456.1	*Ostrinia furnacalis*	377	2E-128	69%
SinsAR2	1125	1011	Yes	27.65	aldo-keto reductase AKR2E4-like	XP_028177948.1	*Ostrinia furnacalis*	506	5E-177	71%
SinsAR3	1738	1092	Yes	25.10	aldo-keto reductase AKR2E4-like	XP_022830935.1	*Spodoptera litura*	553	0	75%
SinsAR4	1252	1032	Yes	26.15	PREDICTED: aldo-keto reductase AKR2E4-like	XP_013136681.1	*Papilio polytes*	498	8E-174	70%
SinsAR5	1242	987	Yes	125.24	aldehyde reductase 7	ATJ44502.1	*Helicoverpa armigera*	507	1E-177	71%
**ATF**										
SinsATF1	1775	1269	Yes	23.49	acetyl-CoA acetyltransferase, mitochondrial	XP_028157143.1	*Ostrinia furnacalis*	777	0	89%
SinsATF2	379	285	Yes	0.47	PREDICTED: acetyl-CoA acetyltransferase, mitochondrial isoform X2	XP_013192024.1	*Amyelois transitella*	177	7E-51	79%

### Acetyl-CoA carboxylase (ACC)

**Fig 5:**
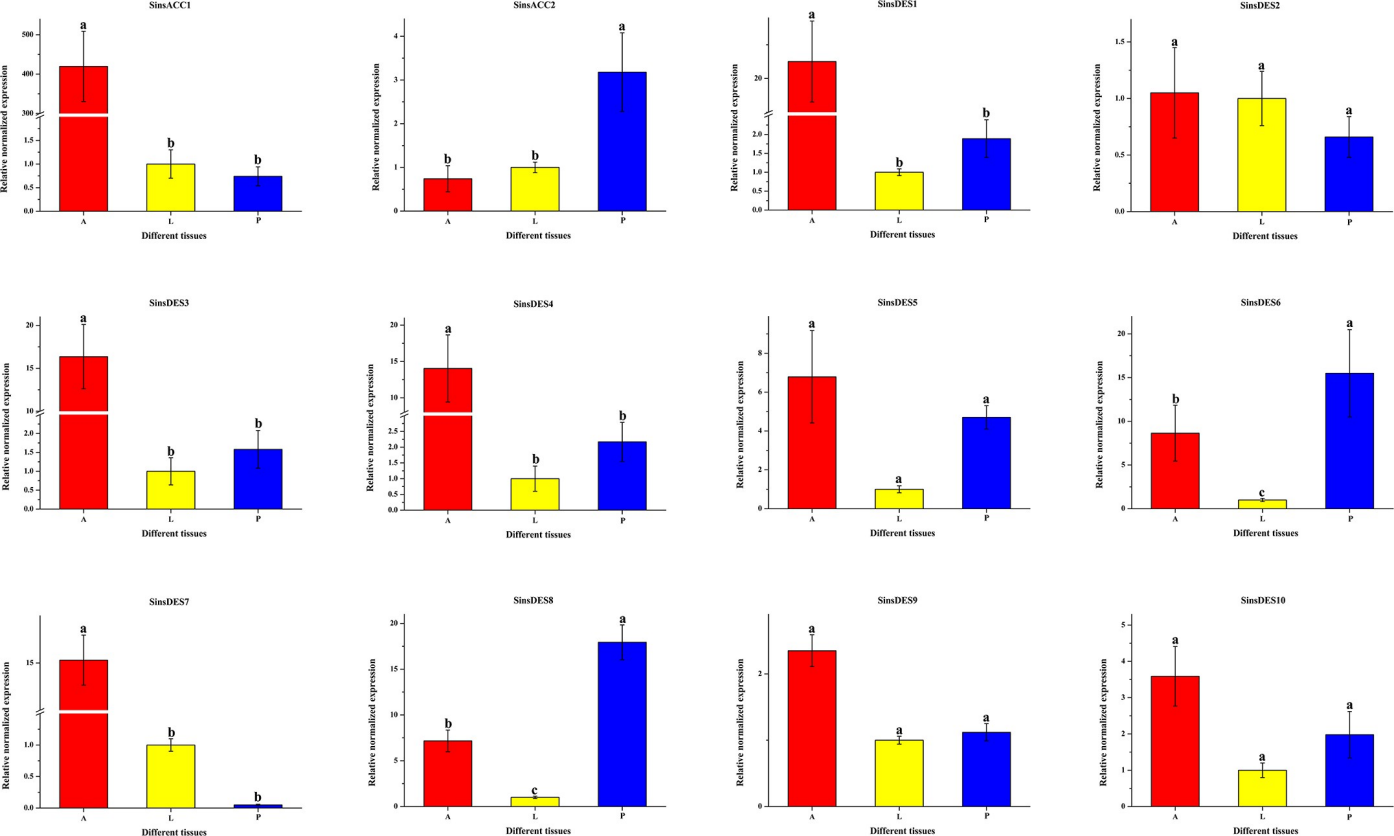
Expression profiles of putative ACCs and DESs in different *S*. *insularis* tissues. A, antennae; L, legs; P, pheromone glands. Actin was used as an internal reference gene for normalizing target gene expression. Standard errors are represented by error bars, and different lowercase letters (a–c) above bars denote significant differences (*p* <0.05).

The first step of saturated long-chain fatty acid biosynthesis is the ATP-dependent carboxylation of acetyl-CoA to malonyl-CoA catalyzed by ACC, a rate-limiting enzyme [13–14]. In the *S*. *insularis* PG transcriptome, we identified two ACCs with lengths of 723 and 7616 bp (Table 2 and S1 Text), similar to the numbers reported previously for other moth species (two in *A*. *ipsilon* [59], one in *P*. *xylostella* [25], and one in *A*. *segetum* [58]). *SinsACC1* with an ORF of 399 bp encodes for an ACC with 63% amino acid identity with the ACC of *Amyelois transitella* (XP_013185423.1), and *SinsACC2* has an intact ORF of 7101 bp that shares high amino acid identity (90%) with the ACC of *Papilio polytes* (XP_013146614.1). The RT-qPCR results (Fig 5) showed that *SinsACC1* was more strongly expressed in the antennae than in the other tissues, whereas *SinsACC2* was mainly expressed in the PG. However, both were present in low abundance (3.6 and 28.33 FPKM) in the *S*. *insularis* PG transcriptome. It was reported that the plastid-specific ACC is inhibited by herbicides that target the eukaryotic form of the enzyme in monocotyledonous plants [62–64]. Eliyahu et al. (2003) subsequently demonstrated that the herbicide diclofop inhibits PBAN-activated sex pheromone production in *Helicoverpa zea*, thereby implicating ACC plays a key regulatory role in fatty acid biosynthesis [65], which provides a basis for the development of a new pest control method based on disruption of sex pheromone production in females.

### Fatty acid synthase (FAS)

FAS is the multifunctional protein that catalyzes acetyl-CoA, malonyl-CoA, and NADPH through-multienzyme complex that catalyzes the synthesis of long-chain fatty acids. Labeling studies demonstrated that palmitic acid (C16) and stearic acid (C18) are the FAS products in most moth PGs [15, 66–67]. Herein, we identified five FASs with lengths ranging from 301 bp to 8170 bp in the *S*. *insularis* PG transcriptome (Table 2 and S1 Text), These results are similar to those reported for other insects, with six and three FASs in *A*. *segetum* [58] and *Sesamia inferens* [68], respectively. Among the five FASs, only *SinsFAS2* has an intact ORF. BLASTX results showed that FASs share high sequence similarity with Lepidoptera FASs in the NCBI Nr protein database (>60%). The FPKM analysis showed that *SinsFAS2* displayed the highest expression level (90.41 FPKM) in the *S*. *insularis* PG.

### Desaturase (DES)

**Fig 6:**
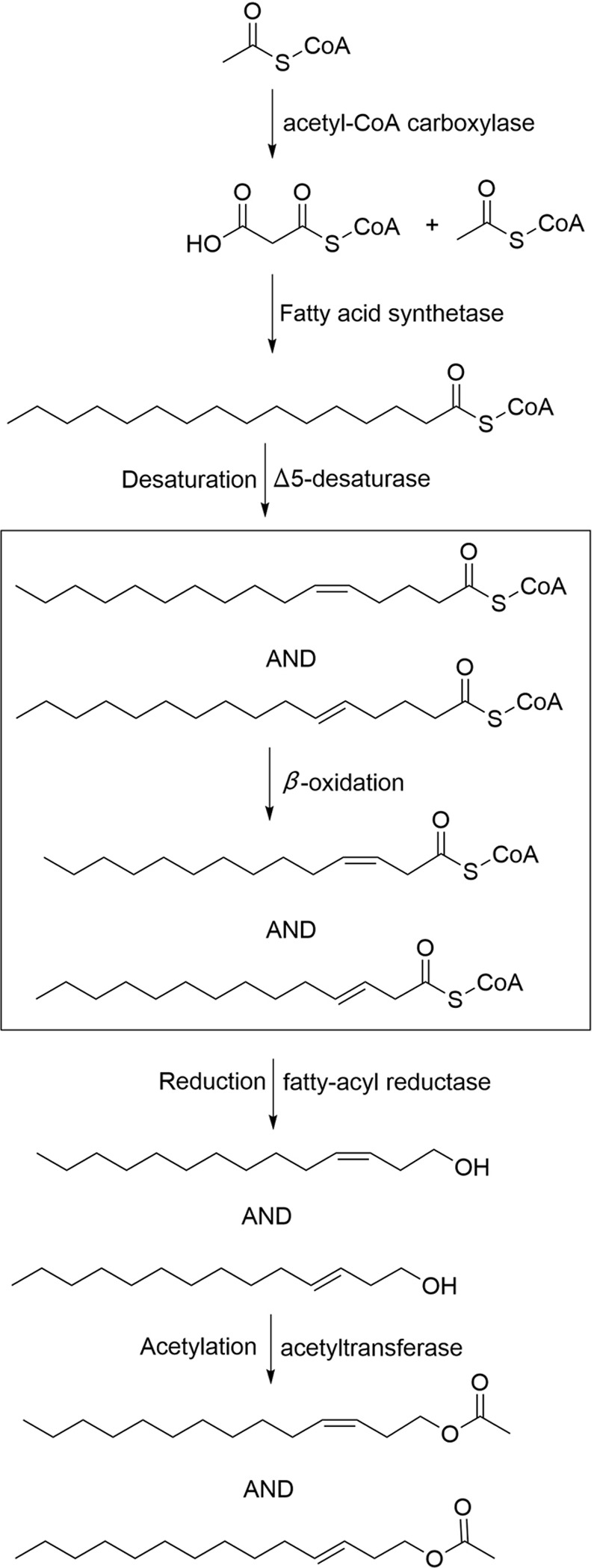
Putative biosynthesis pathway of the sex pheromone components Z3-14:OAc and E3-14:OAc in *S*. *insularis*. The saturated fatty acid precursor palmitic acid (16:0) is desaturated by Δ5-desaturase to form the precursor Z/E5-16:acyl-CoA in the production of two major pheromone components (Z3-14:OAc and E3-14:OAc).

**Fig 7:**
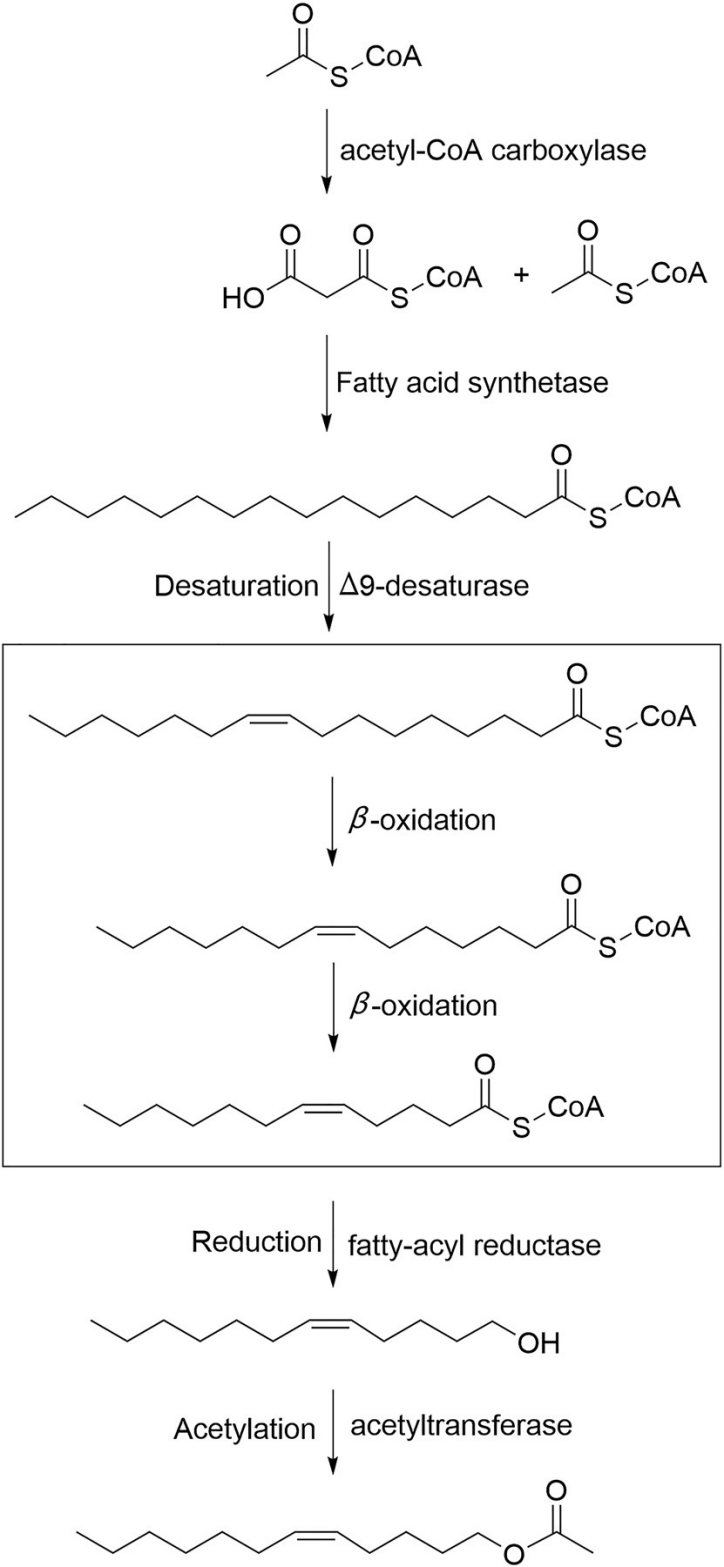
Putative biosynthesis pathway of the sex pheromone component Z5-12:OAc in *S*. *insularis*. The saturated fatty acid precursor palmitic acid (16:0) is desaturated by Δ9-desaturase to form the precursor Z9-16:acyl-CoA in the production of the minor pheromone component Z5-12:OAc.

**Fig 8:**
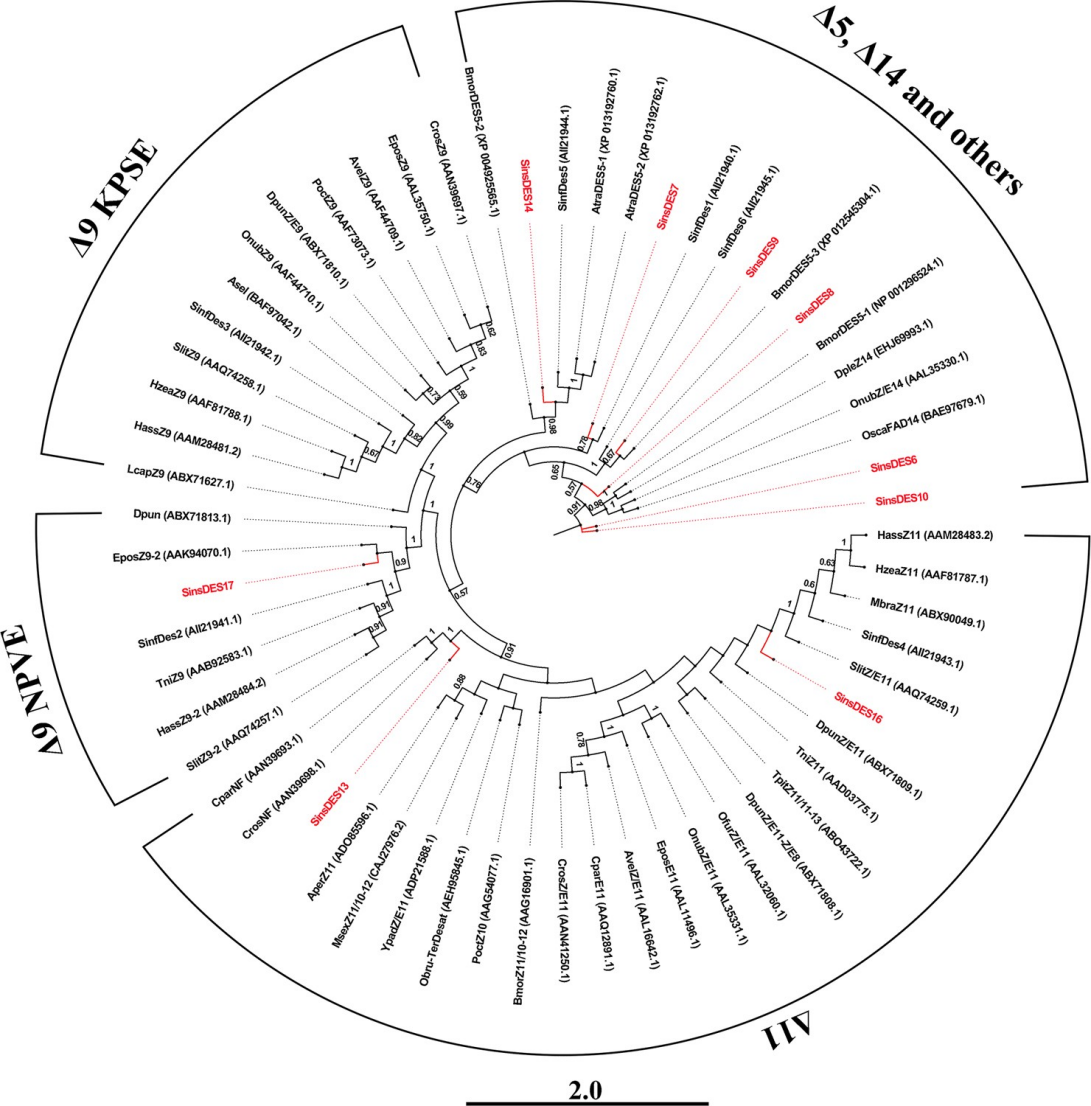
Neighbor-joining phylogenetic tree of selected Lepidopteran DES enzymes. The stability of nodes was assessed by bootstrap analysis with 1000 replicates, and only bootstrap values ≥0.5 are shown at the corresponding nodes. The scale bar represents 2.0 substitutions per site. *S*. *insularis* sequences are colored red.

Double bonds are introduced into the fatty acid chain at specific positions by a variety of desaturases [69]. Three putative sex pheromone compounds of *S*. *insularis* were identified as Z3-14:OAc, E3-14:OAc, and Z5-12:OAc, which are unsaturated fatty acids with acetate esters as the functional group. It is therefore reasonable to assume that the saturated fatty acid precursor of *S*. *insularis* sex pheromones is palmitic acid (C16), which is desaturated by Δ5-desaturase and Δ9-desaturase to form the precursors Z/E5-16:acyl-CoA and Z9-16:acyl-CoA in the production of two major (Z3-14:OAc and E3-14:OAc) and one minor (Z5-12:OAc) sex pheromone component, respectively (Figs 6 and 7). From the *S*. *insularis* PG transcriptome, we identified 17 putative DESs with lengths ranging from 244 to 7148 bp (Table 2 and S1 Text). The number of DESs identified in *S*. *insularis* was more than that in *A*. *ipsilon* [59], *P*. *xylostella* [25], and *A*. *segetum* [58]. Of these DESs, the identity of the best BLASX match in the NCBI NR database ranged from 52% to 89%. Notably, *SinsDES15* identified in the *S*. *insularis* transcriptome shared the highest identity (89%), comparable with *DES1* in *Bombyx mori* (XP_004930794.1). Of the 17 DESs, nine DES sequences were either less than 1000 bp, or no common sites were found for computing distances; thus, we only used the remaining eight *S*. *insularis* DES sequences to construct our phylogenetic tree (Fig 8). In the tree, *SinsDES13* and *SinsDES16* are clustered in the ‘Δ11-desaturases’ clade. The *SinsDES17* sequence shares high sequence homology with ‘Δ9-desaturases’, and it clusters with other enzymes also possessing the NPVE motif. The remaining DESs clustered into the ‘other desaturases’ ortholog clade. The qRT-PCR results (Fig 5) revealed that *SinsDES6* and *SinsDES8* were highly expressed in *S*. *insularis* PG compared with the other tissues, suggesting that they may play roles in *S*. *insularis* sex pheromone production. The other five DESs (*SinsDES1*, *SinsDES3*, *SinsDES4*, *SinsDES6*, and *SinsDES7*) were expressed at significantly higher levels in antennae than in other tissues. All DESs except *SinsDES8* and *SinsDES17* were present at low abundance (from 0.41 to 86.74 FPKM) in the *S*. *insularis* PG transcriptome. DESs play important roles in the generation of structural diversity in Lepidopteran sex pheromone biosynthesis, owing to the evolution of diverse enzymatic properties [22]. Based on the most likely sex pheromone biosynthetic pathways in *S*. *insularis*, both the Δ5- and Δ9-desaturase are likely involved, but it is not clear which of the 17 desaturase genes identified in our study encode these enzymes. Further biochemical analyses of these desaturases are required to determine which ones are involved in pheromone biosynthesis.

### β-oxidation enzymes

After a specific Δ5 or Δ9 double bond is introduced into palmitic acid to form a fatty acyl CoA precursor, the chain of the precursors is then shortened sequentially via a β-oxidation catabolic process to generate different shorter chain pheromone precursors (14C and 12C). Each cycle of β-oxidation involves four reactions: (1) acyl-CoA oxidases (ACOs, in peroxisomes) and acyl-CoA dehydrogenases (ACDs, in mitochondria) act on acyl-CoA to form *E*2-enoyl-CoA; (2) *E*2-enoyl-CoA is reversibly hydrated by enoyl-CoA hydratase (ECH) to form L-3-hydroxyacyl-CoA; (3) L-3-hydroxyacyl-CoA dehydrogenase (HAD) catalyzes the reversible dehydrogenation of L-3-hydroxyacyl-CoA to 3-ketoacyl-CoA; and (4) 3-ketoacyl-CoA is cleaved by 3-ketoacyl-CoA thiolase (KAT) [37, 70–72]. In the *S*. *insularis* PG transcriptome, we identified eight ACO genes, nine ACD genes, three ECH genes, three HAD genes, and one KAT gene (Table 2 and S1 Text). The derived protein sequences of these 24 transcripts share 63–92% amino acid identity with their homologs in other insects. All transcripts were present in low abundance (from 0 to 214.36 FPKM) in the *S*. *insularis* PG.

### Fatty acyl-CoA reductase (FAR)

Chain-shortened fatty acyl CoA precursors are reduced to the corresponding alcohols by alcohol-generating FARs. Fatty alcohols can serve as sex pheromone components in many moths including *Plutella xylostella* [25]. Herein, we detected 13 transcripts homologous to putative FAR genes in the *S*. *insularis* PG transcriptome (Table 2 and S1 Text), similar to the number identified in other moth species (13 in *A*. *ipsilon* [59] and 10 in *A*. *segetum* [58]). Among them, *SinsFAR6* was expressed at the highest level (476.06 FPKM). The FARs in *S*. *insularis* encode proteins shared 46–92% amino acid sequence identity with homologs in other Lepidoptera moths such as *B*. *mori*, *Helicoverpa armigera*, and *Spodoptera exigua*.

### Alcohol dehydrogenase (AD)

Fatty alcohols can also be used as pheromone intermediates to produce corresponding aldehydes by ADs [73]. In the *S*. *insularis* PG, five homologous full-length AD genes were identified (Table 2 and S1 Text). The number of AD-encoding genes in *S*. *insularis* was in accordance with *P*. *xylostella* [25] and *A*. *ipsilon* [59]. Two ADs (*SinsAD1* and *SinsAD4*) encode proteins that are homologous to ADs in *Ostrinia furnacalis* (BAR64763.1 and BAR64764.1) and share relatively high amino acid sequence identity (70%); *SinsAD2* encodes a protein sharing 66% identity with *Sesamia inferens* AD1 (AII21999.1), *SinsAD3* encodes a protein sharing 94% identity with the AD of *Helicoverpa armigera* (XP_021189392.1), and *SinsAD5* encodes a protein sharing 71% identity with the AD of *Cydia pomonella* (AKQ06148.1). FPKM value analysis revealed low expression levels in the *S*. *insularis* PG for all five ADs (FPKM <50).

### Aldehyde reductase (AR)

ARs are a group of the aldo-keto reductases that catalyze the reduction of fatty aldehydes to alcohols [74]. Whether ARs first produce aldehydes which are then converted to alcohols, or vice versa, is very difficult to distinguish in sex pheromone biosynthesis. Herein, we identified five AR genes in the *S*. *insularis* PG transcriptome, and four included intact ORFs (Table 2 and S1 Text). The number of ARs identified in *S*. *insularis* was less than that in *A*. *ipsilon* [59] and *P*. *xylostella* [25]. The deduced protein sequences of these five genes share high amino acid sequence identity (>60%) with their homologs in other Lepidoptera species, and all were expressed at low levels (from 6.56 to 125.24 FPKM) in the *S*. *insularis* PG.

### Acetyltransferase (ATF)

ATF catalyzes the conversion of fatty alcohols to acetate esters, and this is the final enzyme in the pheromone biosynthetic pathway of the *S*. *insularis*. Previous studies showed that ATF is found almost exclusively in the PG, and is active during the photophase and all adult stages [75–76]. ATF is microsomal and exhibits specificity for the Z isomer of 12-, 14-, and 16-carbon monounsaturated fatty alcohol substrates [29–30, 75–76]. However, the enzyme has not been identified at the gene level in any moth so far [58]. In the present study, we identified two transcripts predicted to encode ATFs in the *S*. *insularis* PG (Table 2 and S1 Text). The number of ATF-encoding genes in the *S*. *insularis* PG was in accordance with *P*. *xylostella* [25]. The BLASTX results revealed 89% and 79% amino acid sequence identity shared with putative ATFs of *Ostrinia furnacalis* and *Amyelois transitella* (XP_028157143.1 and XP_013192024.1), respectively. Both ATF transcripts were present at low abundance (23.49 and 0.47 FPKM) in the *S*. *insularis* PG.

## Supporting information

S1 Table Primers used for RT-qPCR analysis of ACCs and DESs in *S*. *insularis*. (DOCX)

S1 Text Nucleic acid sequences of all putative sex pheromone biosynthesis-related genes identified in the *S*. *insularis* pheromone gland transcriptome. (DOCX)

## References

[1] Ando T, Inomata S, Yamamoto M. Lepidopteran sex pheromones. Top Curr Chem. 2004; 239: 51–96. 10.1007/b95449 22160231

[2] Witzgall P, Kirsch P, Cork A. Sex pheromones and their impact on pest management. J Chem Ecol. 2010; 36: 80–100. 10.1007/s10886-009-9737-y 20108027

[3] McNeil JN. Behavioral ecology of pheromone-mediated communication in moths and its importance in the use of pheromone traps. Annu Rev Entomol. 1991; 36: 407–430.

[4] Tillman JA, Seybold SJ, Jurenka RA, Blomquist GJ. Insect pheromones—an overview of biosynthesis and endocrine regulation. Insect Biochem Mol Biol. 1999; 29: 481–514. 10.1016/s0965-1748(99)00016-8 10406089

[5] Löfstedt C, Wahlberg N, Millar JG. Evolutionary patterns of pheromone diversity in lepidoptera In: Allison JD, Cardé RT, editors. Pheromone communication in moths: evolution, behavior and application. Berkeley: University of California Press 2016 pp. 43–78.

[6] Löfstedt C, Hansson BS, Petersson E, Valeur P, Richards A. Pheromonal secretions from glands on the 5th abdominal sternite of hydropsychid and rhyacophilid caddisflies (Trichoptera). J Chem Ecol. 1994; 20:153–170. 10.1007/BF02065998 24241706

[7] Kozlov MV, Zhu JW, Philipp P, Francke W, Zvereva EL, Hansson BS, et al Pheromone specificity in Eriocrania semipurpurella (Stephens) and E. sangii (Wood) (Lepidoptera: Eriocraniidae) based on chirality of semiochemicals. J Chem Ecol. 1996; 22: 431–454. 10.1007/BF02033647 24227484

[8] Raina AK, Wergin WP, Murphy CA, Erbe EF. Structural organization of the sex pheromone gland in Helicoverpa zea in relation to pheromone production and release. Arthropod Struct Dev. 2000; 29: 343–353. 18088939

[9] Jurenka R. Insect pheromone biosynthesis. Top Curr Chem. 2004; 239: 97–132. 10.1007/b95450 22160232

[10] Matsumoto S. Molecular mechanisms underlying sex pheromone production in moths. Biosci Biotechnol Biochem. 2010; 74: 223–231. 10.1271/bbb.90756 20139627

[11] Moto K, Suzuki MG, Hull JJ, Kurata R, Takahashi S, Yamamoto M, et al Involvement of a bifunctional fatty-acyl desaturase in the biosynthesis of the silkmoth, *Bombyx mori*, sex pheromone. Proc Natl Acad Sci USA. 2004; 101: 8631–8636. 10.1073/pnas.0402056101 15173596 PMC423246

[12] Park HY, Kim MS, Paek A, Jeong SE, Knipple DC. An abundant acyl-CoA (Δ9) desaturase transcript in pheromone glands of the cabbage moth, Mamestra brassicae, encodes a catalytically inactive protein. Insect Biochem Mol Biol. 2008; 38: 581–595. 10.1016/j.ibmb.2008.02.001 18405835

[13] Volpe JJ, Vagelos PR. Saturated fatty acid biosynthesis and its regulation. Annu Rev Biochem. 1973; 42: 21–60. 10.1146/annurev.bi.42.070173.000321 4147183

[14] Pape ME, Lopez-Casillas F, Kim KH. Physiological regulation of acetyl-CoA carboxylase gene expression: effects of diet, diabetes, and lactation on acetyl-CoA carboxylase mRNA. Arch Biochem Biophys. 1988; 267: 104–109. 10.1016/0003-9861(88)90013-6 2904242

[15] Bjostad LB, Roelofs WL. Biosynthesis of sex pheromone components and glycerolipid precursors from sodium [1–^14^C] acetate in redbanded leafroller moth. J Chem Ecol. 1984; 10: 681–691. 10.1007/BF00994228 24318604

[16] Foster SP, Roelofs WL. Sex pheromone biosynthesis in the tortricid moth, Ctenopseustis herana (Felder & Rogenhofer). Arch Insect Biochem Physiol. 1996; 32: 135–147.

[17] Wang HL, Liénard MA, Zhao CH, Wang CZ, Löfstedt C. Neofunctionalization in an ancestral insect desaturase lineage led to rare Δ^6^ pheromone signals in the Chinese tussah silkworm. Insect Biochem Mol Biol. 2010; 40: 742–751. 10.1016/j.ibmb.2010.07.009 20691782

[18] Löfstedt C, Bengtsson M. Sex pheromone biosynthesis of (E,E)-8,10-dodecadienol in codling moth Cydia pomonella involves E9 desaturation. J Chem Ecol. 1988; 14: 903–915. 10.1007/BF01018782 24276140

[19] Foster SP, Roelofs WL. Sex pheromone biosynthesis in the leafroller moth *Planotortix excessana* by Δ10 desaturation. Arch Insect Biochem Physiol. 1988; 8: 1–9.

[20] Bjostad LB, Roelofs WL. Sex pheromone biosynthesis from radiolabeled fatty acids in the redbanded leafroller moth. J Biol Chem. 1981; 256: 7936–7940. 7021542

[21] Zhao CH, Löfstedt C, Wang XY. Sex pheromone biosynthesis in the Asian corn borer Ostrinia furnacalis (II): Biosynthesis of (E)-and (Z)-12-tetradecenyl acetate involves Δ14 desaturation. Arch Insect Biochem Physiol. 1990; 15: 57–65.

[22] Knipple DC, Rosenfield CL, Nielsen R, You KM, Jeong SE. Evolution of the integral membrane desaturase gene family in moths and flies. Genetics. 2002; 162: 1737–1752. 12524345 10.1093/genetics/162.4.1737 PMC1462379

[23] Houten SM, Wanders RJA. A general introduction to the biochemistry of mitochondrial fatty acid β-oxidation. J Inherited Metab Dis. 2010; 33: 469–477. 10.1007/s10545-010-9061-2 20195903 PMC2950079

[24] Moto K, Yoshiga T, Yamamoto M, Takahashi S, Okano K, Ando T, et al Pheromone gland-specific fatty-acyl reductase of the silkmoth, *Bombyx mori*. Proc Natl Acad Sci USA. 2003; 100: 9156–9161. 10.1073/pnas.1531993100 12871998 PMC170888

[25] Chen DS, Dai JQ, Han SC. Identification of the pheromone biosynthesis genes from the sex pheromone gland transcriptome of the diamondback moth, Plutella xylostella. Sci Rep. 2017; 7: 16255 10.1038/s41598-017-16518-8 29176628 PMC5701256

[26] Zhang YN, Zhang LW, Chen DS, Sun L, Li ZQ, Ye ZF, et al Molecular identification of differential expression genes associated with sex pheromone biosynthesis in *Spodoptera exigua*. Mol Genet Genomics. 2017; 292: 795–809. 10.1007/s00438-017-1307-3 28349297

[27] Teal PEA, Tumlinson JH. Properties of cuticular oxidases used for sex pheromone biosynthesis by *Heliothis zea*. J Chem Ecol. 1988; 14: 2131–2145. 10.1007/BF01014254 24277148

[28] Fang N, Teal PEA, Tumlinson JH. Correlation between glycerolipids and pheromone aldehydes in the sex pheromone gland of female tobacco hornworm moths, Manduca sexta (L.). Arch Insect Biochem Physiol. 1995; 30: 321–336.

[29] Bestmann HJ, Herrig M, Attygalle AB. Terminal acetylation in pheromone biosynthesis by Mamestra brassicae L. (Lepidoptera: Noctuidae). Experientia. 1987; 43: 1033–1034.

[30] Teal PEA, Tumlinson JH. The role of alcohols in pheromone biosynthesis by two noctuid moths that use acetate pheromone components. Arch Insect Biochem Physiol. 1987; 4: 261–269.

[31] Zhu JW, Zhao CH, Lu F, Bengtsson M, Löfstedt C. Reductase specificity and the ratio regulation of E/Z isomers in the pheromone biosynthesis of the European corn borer, Ostrinia nubilalis (Lepidoptera: Pyralidae). Insect Biochem Mol Biol. 1996; 26: 171–176.

[32] Gao RT, Qin XX. Preliminary study on Holcocerus insularis. For Pest Dis. 1983: 1, 3–5.

[33] Liu HX, Liu ZX, Zheng HX, Jin ZR, Zhang JT, Zhang PQ. Sensilla on the antennae and ovipositor of the carpenterworm, Streltzoviella insularis (Staudinger, 1892) (Lepidoptera, Cossidae). Oriental Insects. 2018; 52: 420–433.

[34] Xu LL, Pei JH, Wang T, Ren LL, Zong SX. The larval sensilla on the antennae and mouthparts of five species of Cossidae (Lepidoptera). Can J Zool. 2017; 95: 611–622.

[35] Zhang JT, Meng XZ. Electrophysiological responses of Holcocerus insularis Staudinger to the female sex pheromone extracts and standard compounds. Scientia Silvae Sinicae. 2000; 36: 123–126.

[36] Zhang JT, Meng XZ. Synthesis and filed tests of sex attractant for Holcocerus insularis Staudinger (Lepidoptera: Cossidae). Scientia Silvae Sinicae. 2001; 37: 71–74.

[37] Vogel H, Heidel AJ, Heckel DG, Groot AT. Transcriptome analysis of the sex pheromone gland of the noctuid moth *Heliothis virescens*. BMC Genomics. 2010; 11: 29 10.1186/1471-2164-11-29 20074338 PMC2820457

[38] Grabherr MG, Haas BJ, Yassour M, Levin JZ, Thompson DA, Amit I, et al Full-length transcriptome assembly from RNA-Seq data without a reference genome. Nat Biotechnol. 2011; 29: 644–652. 10.1038/nbt.1883 21572440 PMC3571712

[39] Conesa A, Götz S, García-Gómez JM, Terol J, Talón M, Robles M. Blast2GO: A universal tool for annotation, visualization and analysis in functional genomics research. Bioinformatics. 2005; 21: 3674–3676. 10.1093/bioinformatics/bti610 16081474

[40] Tatusov RL, Koonin EV, Lipman DJ. A genomic perspective on protein families. Science. 1997; 278: 631–637. 10.1126/science.278.5338.631 9381173

[41] Moriya Y, Itoh M, Okuda S, Yoshizawa AC, Kanehisa M. KAAS: An automatic genome annotation and pathway reconstruction server. Nucleic Acids Res. 2007; 35: W182–W185. 10.1093/nar/gkm321 17526522 PMC1933193

[42] Trapnell C, Williams BA, Pertea G, Mortazavi A, Kwan G, van Baren MJ, et al Transcript assembly and quantification by RNA-Seq reveals unannotated transcripts and isoform switching during cell differentiation. Nature Biotechnol. 2010; 28: 511–515. 20436464 10.1038/nbt.1621 PMC3146043

[43] Tamura K, Peterson D, Peterson N, Stecher G, Nei M, Kumar S. MEGA5: Molecular evolutionary genetics analysis using maximum likelihood, evolutionary distance, and maximum parsimony methods. Mol Biol Evol. 2011; 28: 2731–2739. 10.1093/molbev/msr121 21546353 PMC3203626

[44] Saitou N, Nei M. The neighbor-joining method: a new method for reconstructing phylogenetic trees. Mol Biol Evol. 1987; 4: 406–425. 10.1093/oxfordjournals.molbev.a040454 3447015

[45] Rambaut A. FigTree 1.4.2 software. Institute of Evolutionary Biology, Univ. Edinburgh. 2014; Available from: http://tree.bio.ed.ac.uk/software/figtree/

[46] Livak KJ, Schmittgen TD. Analysis of relative gene expression data using real-time quantitative PCR and the 2-ΔΔCT method. Methods. 2001; 25: 402–408. 10.1006/meth.2001.1262 11846609

[47] Raina AK, Jaffe H, Kempe TG, Keim P, Blacher RW, Fales HM, et al Identification of a neuropeptide hormone that regulates sex pheromone production in female moths. Science. 1989; 244: 796–798. 10.1126/science.244.4906.796 17802237

[48] Rafaeli A, Bober R, Becker L, Choi MY, Fuerst EJ, Jurenka RA. Spatial distribution and differential expression of the PBAN receptor in tissues of adult Helicoverpa spp. (Lepidoptera: Noctuidae). Insect Mol Biol. 2007; 16: 287–293. 10.1111/j.1365-2583.2007.00725.x 17328713

[49] Jurenka RA, Fabrias G, Roelofs WL. Hormonal control of female sex pheromone biosynthesis in the redbanded leafroller moth, *Argyrotaenia velutinana*. Insect Biochem. 1991; 21: 81–89. 10.1016/1367-8280(94)90026-4 7981977

[50] Choi MY, Jurenka RA. Role of extracellular Ca2+ and calcium channel activated by a G protein-coupled receptor regulating pheromone production in Helicoverpa zea (Lepidoptera: Noctuidae). Ann Entomol Soc Am. 2006; 99: 905–909.

[51] Choi MY, Fuerst EJ, Rafaeli A, Jurenka RA. Identification of a G protein-coupled receptor for pheromone biosynthesis activating neuropeptide from pheromone glands of the moth *Helicoverpa zea*. Proc Natl Acad Sci USA. 2003; 100: 9721–9726. 10.1073/pnas.1632485100 12888624 PMC187832

[52] Hull JJ, Ohnishi A, Moto K, Kawasaki Y, Kurata R, Suzuki MG, et al Cloning and characterization of the pheromone biosynthesis activating neuropeptide receptor from the silkmoth, *Bombyx mori*—Significance of the carboxyl terminus in receptor internalization. J Biol Chem. 2004; 279: 51500–51507. 10.1074/jbc.M408142200 15358772

[53] Kim YJ, Nachman RJ, Aimanova K, Gill S, Adams ME. The pheromone biosynthesis activating neuropeptide (PBAN) receptor of Heliothis virescens: Identification, functional expression, and structure-activity relationships of ligand analogs. Peptides. 2008; 29: 268–275. 10.1016/j.peptides.2007.12.001 18243415 PMC3900413

[54] Lee JM, Hull JJ, Kawai T, Goto C, Kurihara M, Tanokura M, et al Re-evaluation of the PBAN receptor molecule: characterization of PBANR variants expressed in the pheromone glands of moths. Front Endocrinol. 2012; 3: 6. 10.3389/fendo.2012.00006 PMC3356081 22654850

[55] Hull JJ, Ohnishi A, Matsumoto S. Regulatory mechanisms underlying pheromone biosynthesis activating neuropeptide (PBAN)-induced internalization of the Bombyx mori PBAN receptor. Biochem Biophys Res Commun. 2005; 334: 69–78. 10.1016/j.bbrc.2005.06.050 15992769

[56] Moore CA, Milano SK, Benovic JL. Regulation of receptor trafficking by GRKs and arrestins. Annu Rev Physiol. 2007; 69: 451–482. 10.1146/annurev.physiol.69.022405.154712 17037978

[57] Marchese A, Paing MM, Temple BR, Trejo J. G protein—coupled receptor sorting to endosomes and lysosomes. Annu Rev Pharmacol Toxicol. 2008; 48: 601–629. 10.1146/annurev.pharmtox.48.113006.094646 17995450 PMC2869288

[58] Ding BJ, Löfstedt C. Analysis of the *Agrotis segetum* pheromone gland transcriptome in the light of sex pheromone biosynthesis. BMC Genomics. 2015; 16: 711 10.1186/s12864-015-1909-2 26385554 PMC4575462

[59] Gu SH, Wu KM, Guo YY, Pickett JA, Field LM, Zhou JJ, et al Identification of genes expressed in the sex pheromone gland of the black cutworm *Agrotis ipsilon* with putative roles in sex pheromone biosynthesis and transport. BMC Genomics. 2013; 14: 636 10.1186/1471-2164-14-636 24053512 PMC3849270

[60] Nusawardani T, Kroemer JA, Choi MY, Jurenka RA. Identification and characterization of the pyrokinin/pheromone biosynthesis activating neuropeptide family of G protein-coupled receptors from *Ostrinia nubilalis*. Insect Mol Biol. 2013; 22: 331–340. 10.1111/imb.12025 23551811

[61] Fodor J, Hull JJ, Köblös G, Jacquin-Joly E, Szlanka T, Fónagy A. Identification and functional characterization of the pheromone biosynthesis activating neuropeptide receptor isoforms from *Mamestra brassicae*. Gen Comp Endocrinol. 2018; 258: 60–69. 10.1016/j.ygcen.2017.05.024 28579335

[62] Golz A, Focke M, Lichtenthaler HK. Inhibitors of *de novo* fatty acid biosynthesis in higher plants. J Plant Physiol. 1994; 143: 426–433.

[63] Sasaki Y, Konishi T, Nagano Y. The compartmentation of acetyl-coenzyme A carboxylase in plants. Plant Physiol. 1995; 108: 445–449. 10.1104/pp.108.2.445 12228484 PMC157362

[64] Harwood JL. Fatty acid metabolism. Annu Rev Plant Physiol Plant Mol Biol. 1988; 39: 101–138.

[65] Eliyahu D, Applebaum S, Rafaeli A. Moth sex-pheromone biosynthesis is inhibited by the herbicide diclofop. Pestic Biochem Phys. 2003; 77: 75–81.

[66] Tang JD, Charlton RE, Jurenka RA, Wolf WA, Phelan PL, Sreng L, et al Regulation of pheromone biosynthesis by a brain hormone in two moth species. Proc Natl Acad Sci USA. 1989; 86: 1806–1810. 10.1073/pnas.86.6.1806 16594018 PMC286793

[67] Jurenka RA, Jacquin E, Roelofs WL. Stimulation of pheromone biosynthesis in the moth *Helicoverpa zea*: Action of a brain hormone on pheromone glands involves Ca^2+^ and cAMP as second messengers. Proc Natl Acad Sci USA. 1991; 88: 8621–8625. 10.1073/pnas.88.19.8621 11607216 PMC52561

[68] Zhang YN, Xia YH, Zhu JY, Li SY, Dong SL. Putative pathway of sex pheromone biosynthesis and degradation by expression patterns of genes identified from female pheromone gland and adult antenna of Sesamia inferens (Walker). J Chem Ecol. 2014; 40: 439–451. 10.1007/s10886-014-0433-1 24817326

[69] Hashimoto K, Yoshizawa AC, Okuda S, Kuma K, Goto S, Kanehisa M. The repertoire of desaturases and elongases reveals fatty acid variations in 56 eukaryotic genomes. J Lipid Res. 2008; 49: 183–191. 10.1194/jlr.M700377-JLR200 17921532

[70] Ikeda Y, Okamura-Ikeda K, Tanaka K. Purification and characterization of short-chain, medium-chain, and long-chain acyl-CoA dehydrogenases from rat liver mitochondria. Isolation of the holo- and apoenzymes and conversion of the apoenzyme to the holoenzyme. J Biol Chem. 1985; 260: 1311–1325. 3968063

[71] Kunau WH, Dommes V, Schulz H. β-Oxidation of fatty acids in mitochondria, peroxisomes, and bacteria: A century of continued progress. Prog Lipid Res. 1995; 34: 267–342. 10.1016/0163-7827(95)00011-9 8685242

[72] Uchida Y, Izai K, Orii T, Hashimoto T. Novel fatty acid β-oxidation enzymes in rat liver mitochondria. II. Purification and properties of enoyl-coenzyme A (CoA) hydratase/3-hydroxyacyl-CoA dehydrogenase/3-ketoacyl-CoA thiolase trifunctional protein. J Biol Chem. 1992; 267: 1034–1041. 1730633

[73] Sofer W, Martin PF. Analysis of alcohol dehydrogenase gene expression in *Drosophila*. Annu Rev Genet. 1987; 21: 203–227. 10.1146/annurev.ge.21.120187.001223 3327463

[74] Bohren KM, Bullock B, Wermuth B, Gabbay KH. The aldo-keto reductase superfamily. cDNAs and deduced amino acid sequences of human aldehyde and aldose reductases. J Biol Chem. 1989; 264: 9547–9551. 2498333

[75] Jurenka RA, Roelofs WL. Characterization of the acetyltransferase used in pheromone biosynthesis in moths: specificity for the *Z* isomer in Tortricidae. Insect Biochem. 1989; 19: 639–644.

[76] Morse D, Meighen E. Biosynthesis of the acetate ester precursor of the spruce budworm sex pheromone by an acetyl CoA: fatty alcohol acetyltransferase. Insect Biochem. 1987; 17: 53–59.

