# Supplementary material for: Identification of putative Type-I sex pheromone biosynthesis-related genes expressed in the female pheromone gland of Streltzoviella insularis
Source: PLoS One. 2020 Jan 16;15(1):e0227666. doi: 10.1371/journal.pone.0227666 (PMC6964838; doi:10.1371/journal.pone.0227666)
Supplement: S1 Table — (DOCX) [file pone.0227666.s001.docx]

**S1 Table. Primers used for RT-qPCR analysis of ACCs and DESs in *S. insularis*.**

| **Gene name** | **Forward primer** | **Reverse primer** |
| --- | --- | --- |
| *SinsACC1* | TTGAACTGCATCGTCTTTGG | AGGCACAGCAGTACACAACG |
| *SinsACC2* | ATTGCGTTCTTCTTCGGTGT | TTTCGTCGTGTCTGACTTCG |
| *SinsDES1* | TCGGAAAATCCTGATGAAGG | ACTGTGGTTCGGAAGTCACC |
| *SinsDES2* | CGTGTTCATCGAAGCTATGG | AACTGCGCCTTCAGTTTCTC |
| *SinsDES3* | ACATTATGCCAGCCGAAAAC | CCCATCCAATTAAAGCGAAA |
| *SinsDES4* | CAGAAGGGGAGCAACAGTTC | GTGGCCCATTTAGCAGATGT |
| *SinsDES5* | GAACTGGAGACGGCACTCAT | TGTTCTGTTGCCCAAAATCA |
| *SinsDES6* | TGGGATGGTTGATGATGAAA | ACTGCACTGGTACCCAAACC |
| *SinsDES7* | AAACCCGGACAAAAGTACCC | AGACCCAACGCTGCAAATAC |
| *SinsDES8* | ACACGCCTTCGGTTACAAAC | TATGCCCAGCCTATTCGTTC |
| *SinsDES9* | TCTGGGGACAGAGCTTGACT | AGGCGTGATGGAAATTATGC |
| *SinsDES10* | GTGACTGGACCCAAAGGAGA | ACATATTGCCAGCCGTTTTC |
| Actin | CGACAGGATGCAGAAGGAAA | TAGAAGCACTTGCGGTGGAC |
