# Supplementary material for: Identification of putative Type-I sex pheromone biosynthesis-related genes expressed in the female pheromone gland of Streltzoviella insularis
Source: PLoS One. 2020 Jan 16;15(1):e0227666. doi: 10.1371/journal.pone.0227666 (PMC6964838; doi:10.1371/journal.pone.0227666)
Supplement: S1 Text — (DOCX) [file pone.0227666.s002.docx]

**S1 Text. Nucleic acid sequences of all putative sex pheromone biosynthesis-related genes identified in the** ***S. insularis* pheromone gland transcriptome.**

**PBANR:**

>SinsPBANR

TATGCCGAACCTGACTCCTTGGATTTGTTGGTACCATTGAGCGTCGCTTATACGGTTATTTTCGTCGCCGGGATCCTAGGAAACACAAGCACATGCGTCGTCATCGCACGTAATCGCTCAATGCATACAGCTACCAACTTCTATCTGTTCAGTCTCGCTATATCGGATGTAATACTTCTAGTTTGCGGTCTTCCACTTGAACTGCATCGTCTTTGGAATCCATCCACTTATCCGCTCGGCGAACCGGCTTGCATAGCATTCGGATTGGCCTCTGAAACGTCCGCGAACGCAACGGTGTTAACAATTACAGCTTTCACTGTGGAAAGGTATATTGCTATATGTAAACCTTTCATGTCGCATACGATGTCGAAGTTGTCTCGAGCGGTGCGTTTCATATTCGCTATATGGGTGTTAGCGTTGTGTACTGCTGTGCCTCAAGCTATGCAATTCGGAATAGTGACGGATGTCGAGAATGGACAGAATATAAGTATATGTACAGTGAAAGGTCACGGTGTTCATCAAGTGTTTGTTATATCGAGCTTTGTGTTTTTTGTGGTGCCAATGTCATTGATATCCGTGCTATACGCCTTAATTGGTGTCAAATTGCGAACGTCTCGTGTTTTACATCCCGTGAAAAAACTATCAGTTGAGAGTAATGGACGCATTAGCGGAAGCACTAGATATCGTAATGGAGCTTCGCAACGGAGAGTTATAAGAATGTTAGTGGCGGTGGCGCTTTCTTTCTTCGTATGCTGGGCACCATTCCACGTCCAGAGGCTGCTAGCGATCTATGGCAAGAGTTTAGAGCATCCATCTGACACTTTTTATCTGGTGTATATAGTGCTGACCTATTTATCGGGTGTCCTGTACTTTCTGTCGACTGCTATCAACCCATTCCTGTACAACATTATGTCGAATAAGTTCAGGAATGCTTTCAAGGTGACATTATCAGCGTGGTGCGGTAGAAGATCAGGGCCACGCTTGGGAAGGACGTACAGTGCGTTACAAGCGTCGCAGAGACAGCGCACTGTCGTCGGCACGGACAGACCGCGCGGGCCGCGGCGTTTGCGCAGGCTCTCCACTGCCACCACGCAACTGTACGACGCGCCTCCGAGAGCGCAGTGCTACAACCCCCAAGATCTTTCAATCGTGAACGAATCGCCCAGTGCTAACGGGCGGTGGGGACGTGCGTGGCGACCACGGAATAATGAAACTTCGGATTCCGTTGGTTCTCCACGAAGCATTTCCAATTCCAGTCTACGTGAAGTTGATGAAGAACTCACCGGCGAAGAGCTTGCCACATACATGTACCAAGTGAACTGTGATATAGGCGGACTCACCTGAGACACGTCAGTTTAGACTGTCGAATAGACAGATGGTTGATGACAGCACTAGGAATAAGTGGTGAAGTCACTCGTTGCCTAGTATTAGCGGTGATGGCTGAATAGTTTTCGTAGAAACGGAAGAATTGTGCAATGCATTTTATAAATGCCCACTCTCGATTGTTGTTGAATGGTTTGATCGAAGTGCAAGCTGTA

**ACC:**

>SinsACC1

ACGGTGCCTTGAGACATAGACGGCCTCAATTTGCTACGTTTCTCAGCTAAAGCCAACTGCTGGGCCGGAGTGAGGACTCGACGTATGTTAGGCGGTGGGGCGTTTGGAAACGAGTCCCGGCCCTCGTGGGAGGGTCCGTCGTCATCTTCCGCCACGCCAGGGTCGGAGGTCTCGTCGCCGACAATGAAGTCGATTTTATCGCCATTTTCGTCGTGTCTGACTTCGTCCGAGTCGATCTTCTCGAAGTTTTCGCTCTCGCTCGACGAGCCCCTCACTTGTTCTTGCTGATCAGCCATTTTGTCACTTCGTGAAATTTCCTGCACGATACGTTTTCTCGTGAGAAACTTAATTAGGTAACATACGAAAACAACACCGAAGAAGAACGCAATTGTAAATAACATCAAAACATCACAACACTGTCACAAAACCATGATTAATTTATTATTTCAGAAAATCCGAATAAAACTACGTCTTCCCGCCAACTATTTGACAGGCGACTGTCGTATACCGCGACTGATACACTCAGCGATACAATAATTTATTATGAGTGAAAAAAAAATACAATTATTCAATCGCTGTAATCACATTAACCGATAAATTTGACGTGTAATATAAACTTTAAATTTTAGACCAAACGCTGATCACTTCTTAAACTGTTTATTTAAATATTTAAAAGGCATGAAACGACATTCTCGGTTCACGTTTTCAAGAGACAACAATATT

>SinsACC2

ATTTAAACTTCGATCTAACCTCACGTCTATATCTGTTTATTATTTATACATTTAGTTTGCATATCGTCGATTTTCAATCCGAATTCAAAATTCTGAGAATAACAATATTAATTTGGTTTGAAACGAAAGTGAAATTGATAGTGAAAATGTGAAAAAGAAAAACAATAGTGTTTATGAGTCTAACGAACACGTTAGCTTCGTTAGTGACGATAACGAATTACGTGAGGAATTTCATAACAATGTTGAAGAAGAGAAGGACCTCGAAACGGTTCGTGCTCGGAGACACCACCGACGAGGGGACCCCGTCATGGGAGAACGAAGATGAAACTGCAACTATACCTTCGAATGTTCAAAAATTTCAAATGACCTTAGAGTCAGAACCTGATAGGCAACAAGATGATGGTCATGATAAGGAGCAAGATGGAAGGTTGCTGAGGCCACCGAATGATCGTACTTTGCAGCCGTCTATGTCTCAAGGCACCGTGATTCACAGTCAGCGATTCCAAGAGAAAGATTTCACTGTCGCCACTCCAGAAGAGTTCGTGAGACGTTTCCAAGGGACCAAGCCTATTAATAAGGTTTTGATTGCGAACAACGGTATCGGCGCTGTAAAATGTATGCGTTCAATCAGAAGATGGTCCTACGAGATGTTCAAGAACGAACGAGCTGTTCGCTTTGTTGTTATGGTAACACCGGAAGACTTAAAAGCGAACGCGGAATACATAAAAATGGCTGACCACTACGTACCAGTGCCCGGCGGCTCCAACAATAACAATTATGCGAATGTGGAACTTATTGTGGACATCGCTATCAGGACCCAAGTACAGGCCGTCTGGGCAGGATGGGGCCACGCCTCCGAGAATCCAAAGCTGCCCGAACTGCTTCACCGCGCCGGCGTGGTTTTCATCGGTCCCCCGGAAAAAGCCATGTGGGCCCTTGGAGACAAGATCGCTTCATCCATCGTGGCTCAAACTGCCGATATACCAACTTTGCCTTGGAGTGGGAGTGAATTAAAGGCGGAATACAATAGTAAGAAGATTAAGATATCGTCAGAGCTATTCGCTAGAGGTTGCGTGACGTCTCCCGAACAGGGATTGCAAGCCGCTAATAGAATTGGTTTTCCTGTGATGATCAAGGCTTCAGAAGGCGGCGGTGGCAAAGGTATTAGGAAAGTGGAGAACCCAGATGACTTTAACAACATGTTCAGACAGGTGCAAGCGGAAGTTCCCGGATCACCAATATTTGTGATGAAGTTGGCCAAGTGTGCTCGGCATCTGGAAGTGCAGTTGTTGGCCGATCAGTATGGTAATGCGATCTCTCTGTTCGGCCGCGATTGCTCCATCCAGCGCCGTCATCAGAAGATCATCGAGGAAGCGCCCGCCGCCATCGCCAAACCCGACGTGTTCATTGAAATGGAGAAGGCTGCAGTACGTCTAGCCAAAATGGTTGGATATGTCAGCGCCGGTACCGTGGAATATTTGTATGATCCAGCCACCGGATCCTACTTCTTTTTGGAGCTCAACCCTCGTCTGCAAGTGGAACATCCTTGCACGGAGATGGTAGCCGATGTCAATCTGCCCGCAGCTCAGTTGCAGATCGCCATGGGTCTCCCTCTTTATCACATCAAAGACATTCGTCTTCTATATGGCGAGTCTCCGTGGGGCATGACCCAGATAGACTTTGATGAACCGAAACAAAGACCCTCGCCGTGGGGACATGTGATCGCCGCTAGGATAACTTCGGAAAATCCTGATGAAGGCTTCAAACCATCTTCCGGTACCGTACAAGAGCTAAACTTCAGGTCTTCTAAAAACGTGTGGGGTTACTTCAGTGTGGCGGCTTCAGGAGGTCTTCACGAGTTCGCAGACTCTCAATTTGGACATTGCTTCTCGTGGGGCGAGACCAGAGAACAAGCTAGAGAAAACTTGGTAATCGCTCTTAAAGAGCTGAGTATCCGAGGTGACTTCCGAACCACAGTCGAGTACCTCATCACATTACTGGAGACTAGCGCCTTCCAGGACAACAATATCGATACCAGCTGGCTTGATGCGCTCATCGCTGAGAGAGTACAATCGGAGAAACCCGACGTAATGTTGGGCGTGATATGTGGATCCATATTGATAGCCGATAGCATCTTCACGGCGAATTTCCAAGAGTTCAAGAGCGGTTTGGAAAAGGGTCAAATCCAAGGTTCCGGCCAGTTAACCAACTATGTGGAAGTTGAGTTGATTCACAGCGGTGTCAAATACAAAGTTCAAGCGACAAGATCTGGACCAACTTCATACTTCTTAGCAATGAATGGGAGTTTTAAGGAGTTGGAAGTGCATAAGCTGACTGATGGTGGCACTCTGTTGTCGGTCGATGGAGCTTCTTTCACGACATACTTGAAAGATGAAGTTGACAAATATAGAATAGTAATTGGCAATCAAACTGTTGTGTTCGAAAAAGAAAAAGACCCTTCGAAATTGAGGGCGCCATCTGCCGGCAAATTGATCAATACTTTAGTTGAAGATGGCGGACATGTCGATAAGGGTCAACCTTACGCTGAAATCGAGGTGATGAAAATGGTGATGACTTTATCATCTCCCGAATCTGGTAAAGTAACATGGAATCTACGGCCAGGAGCGGTTTTGGACATGGGAGCACTTATTGGCACATTAGAACTGGACGATCCATCGCTAGTGACAACAGCACAACCGTATAAGGGCCAGTTCCCACTGGAAGACACTCCTACTGTGTCCGACAAACTGAATCATGCCCATAACAAGTTCAGGACCATTTTGGAGAATACACTCGCTGGTTATTGTCTCCCTGAGCCATACAACACGCCAAGATTGCGCGAAGTTGTGGAAAAGTTCATGCAGAGCTTACGCGATCCGTCCTTACCACTTTTGGAGTTGCAAGAGGTATTATCGTCAGCGTCGGGTCGCATACCGGTAGCGGTGGAGAAGAAAGTACGCAAATTGATGGCTTTATATGAAAGGAACATCACTAGTGTGTTGGCACAGTTCCCGAGTCAGCAGATAGCCAGCGTTATTGACCATCACGCAGCTTCGCTGGCGAAGAGATCCGACAGGGACAACTTCTTTATGAGTACTCAGGCGCTCGTCGTTTTGGTACAAAGATACAGAAACGGCATCCGCGGTCGAATGAAGGCCGCCGTCCACGATTTGCTCAAACAGTACTATCAAGTGGAGAGCAATTTCCAATTAGGTTCGTACGACAAATGCGTGATGGCGCTCCGCGATCGCTACAAGGACGACATGCAAGCGGTCGCCGATATTATATTCTCGCACAATCAAGTCGCTAAGAAGAATTTGTTGGTAACCCTCTTAATAGACCATCTGTGGTCCAACGAGCCCGGCCTCACGGACGAGCTGGCGGCGACACTGAACGAGTTGACGTCGCTGCATCGCGCCGAGCACAGCCGCGTCGCGCTGCGCGCCCGGCAGGTGTTGATAGCAGCCCACCAACCGGCATACGAGCTTCGCCACAATCAGATGGAGTCCATATTCCTCTCAGCGGTGGACATGTACGGCCACGACTTCCATCCGGAGAACCTGCAGAAGCTGATATTGTCCGAGACGAGCATATTCGACATACTGCACGATTTCTTCTATCATTCCAATGCAGCGGTATGTAATGCTGCTTTAGAAGTTTACGTGCGTCGCGCTTATACGTCGTACGAGATAAATTGCTTGCAGCACTTGGGTTTGTCCGGAGAACTCGGAATTGTCCACTTCCAGTTCGTGTTGCCCACCGGACATCCTAACAGGATACCTATAAGTCAAACGGAAATCGAACTGGCGTCGGCTCAGGACCAAGAAGGGATTCCAGCAGAACTCTGTACAGCGGCGATGAGGAAGTGTCATCACCGCACCGGGGCTTTGGCGGCGTTTACATCGTTTGAGCAGTTCCTGCAGTACGCTGATGAATTGCTCGACTTAGTCCATGACTTCGCGAGCAACGCTACCGTTAGACGTGAGGACTTACAAGCTCTGCAGGAAGGCAGCGAGAGTCGCGACAGCACCAGCATCAACATGGGCTTGGAGTATGGCAAGAACCCGGACGCTGAGGATGTGCCATTGGAGCCAATTCACATTCTCATGATCGGCGTTCGCGACAACGGCGAAAGCGATGACAGCGCGCTGTCGCGCCGCTTCGGCGGCTTCTGTCGCGCGCACCGGCACGAGCTGCATCAGAAACGGATCAGGCGTGTCACCTTCATGTCGCTTATCAAGCGTCAATTCCCCAAGTTCTTCACGTATCGCGCGCGCAACGACTTCACGGAGGACACAATTTACCGTCATCTTGAACCAGCTTCCGCCTTCCAGTTGGAACTGTACCGCATGAGGAGCTACGAGCTGGAAGCTTTACCCACCAGCAACCAGAAGATGCATCTTTATTTGGGCAAAGCGAAGGTGAAGAAAGGGCAGGAGGTGACGGATTACCGTTTCTTCATCCGCTCTATCATCCGACACCAGGACCTGATCACTAAGGAGGCCAGCTTCGAGTATCTCCAGAATGAGGGTGAAAGAGTGCTCTTGGAAGCGATGGACGAACTGGAAGTGGCATTCTCTCATCCGTTGGCGAAGAGAACGGATTGCAATCACATATTCTTGAATTTTGGACCCACAGTCATTATGGACCCTGCTAAGATCGAAGAGTCCGTCCTTGGTATGGTGATGCGATATGGACCCAGGTTATGGAAACTTAGGGTTTTGCAGGCGGAGATAAGATTTACATTGAGAATTGGACCCGGAGCTCCAACGAAGAATGTGCGATTGTGCCTGTCCAATGGATCTGGATATTCCCTGGACGTTTACTCGTATGAGGAGGTTTCTGATCCCAGGACCGGAGTGATAATGTTCCAATCGTACGGTCCGAGACAAGGCCCCATGCACGGATTGCCGATATCTACTCCGTATGTCACCAAAAATTATTTACAACAGAAGAGATTTTTGGCGACGTCACAAGGCACGACATACGTGTATGACATACCGGACATGTTCAGGCAGATGGTCGAACGGAGATGGAGGGAATGTATCGAAGATGGCAGCGTTGATGGTCCCATGCCGGACAATGTGATGTCGTCGGTGGAGCTGGTCGTGGAACCGGACGGCGAGCGGCGAGTGGTCGAAGTTACCCGGCTGCCCGGGCAGAATAATGTGGGTATGGTGGCATGGCGTTTAACACTTCTGACGCCCGAATGTCCTGACGGTCGTGATATCATACTCATTGCGAACGATCTCACCTACTATATGGGGTCTTTTGGACCCCAAGAAGATTGGGTGTATTATAAAGCGTCGCAGTACGCTAGGGAGTTGAAGATACCCAGGATATACGTGAGTGTGAACTCAGGCGCCCGTATCGGTGTAGCCGAGGAGGTCAAATCGGAGTTCAACGTCGCTTGGCTCGATTCCGAGAGACCCGAGCGTGGCTTCAAATATCTCTATTTGACACCGGAATCGTACTCCAAGCTGGGGCCATTGGGGTCCGTGAAGAGCCAGCTGATTGAGGACGAAGGAGAATCGAGATATAAGATCACGGATATTATTGGCAAGGAAGACGGTCTCGGCGTGGAATGCCTGCGCGACGCGGGGCTGATAGCGGGCGAGACTGCGCAGGCCTACGAGGAGGTGGTCACCATCTCCGTGGTCACGTGTCGCGCTATCGGCATCGGCTCCTACGTAGTACGTCTCGGCCACCGAGTGATCCAAGTGGAGAATTCATACATTATCCTAACCGGTTACGCGGCCCTGAACAAAGTGCTCGGTCGTCCAGTGTACGCCAGCAACAACCAGCTTGGCGGCATTCAGATCATGCACAACAATGGAGTCACCCACGCGATCGCGCCGACCGATTTGGAAGCGATACGGACCGCCGTCAGGTGGTTGTCGTATGTGCCTAAGGACAAACTGAGCACAGTGCCTATAATGCGCTGCTCGGACCCAATAGACCGTCCGGTCGAGTGGGTCCCGCCGCGCGCCGCCCACGACCCTCGCCTTATGCTGACAGGAGACGGAGCCCGTGCTGGATTCTTCGATATCGGCTCCTTCGATGAAGTCATGAAGCCCTGGGCGCAGACTGTCATTGCTGGTCGAGCACGTCTGGGCGGTATACCGGTGGGCGTGGTGGCAGTTGAAACGAGGACTGTGGAGTTCACTCTGCCGGCGGACCCGGCCAACTTGGACTCCGAGGCTAAAACAGTTCAGCAGGCCGGACAGGTATGGTTCCCCGATTCGGCGTACAAGACTTCACAGGCCATCAACGACTTCTCCCGCGAGAATTTACCGATCATAATATTCGCCAACTGGCGTGGATTTAGCGGCGGACAGAAGGACATGTACGAACAAATTCTGAAATTCGGAGCGGAGATAGTTCGCGCGTTGCGCGGAGCCACCGCGCCCGTGCTGGTGTACATTCCCCCCGGGGCCGAACTGCGCGGAGGCGCCTGGGCCGTGGTGGACCCCAGCGTCAATAACTTGCGGATGGAGATGTACGCCGACAGCGACGCTAGGGGCGGTGTGTTGGAAGCGGAGGGTATAGTCGAGGTGAAGTTCAAACAGCGAGACATATTGAAAACTATGCACCGGCTGGATCCGGAATTGCAACGTCTTGGTGCCAGGATATCCGAATTAAAGGATCAAATTAAAGAGATATCCAAGAACTTCGATAGAAGAGGATCCATAGACGATGTCTTGATCAAAACCGATGCCGGGAAACAAGCGGAAAGCAAAGTTCGTGAATTAGAAACTGAGCTGTTAGCGGCGGAGAAAACGGCCAAAGCGCGGGAAAAGGAACTCAGCCCCATTTACCACGAGATCGCCGTCCAGTTCGCAGAGTTGCACGACACCGCTGAAAGAATGTTAGAGAAAGGCTGCATATTTGACATAATACCGTGGCGCGAGTCCCGGCGGCTGCTGCATTGGCGGCTGAAGCGACTGCTGCGTCAGAACGCGCAGGAGCGGCGCGTGCAGGCGGCCGTGGGCCCCGCCGACAGCATGGACCAGGGCGCCGCCGCCGCCACGCTGCGCCGATGGTTCACCGAGGACCAGGGCGAGACGCAGTCCCACCGTTGGGAGCAGGACAACGAGGCCGTGTGCAAGTGGCTGGAGGCGCAGGCGGCCGACGACGGCTCCGTGCTCAACCGCAACCTGCGCGCCATCAAGCAGGACTCCGTGCTCAGCGTGGTCAACACCCTCGTGCTGGAGTTGACCCCATCACAACGTTCAGAGTTCATAAGGAAACTTTCCGCTTTGGATTTAGAATAACAATTCTCAGCGTCACTCATTTATGAGGGTAGGATGAAAAATAAATTCTATAAATATTAGTTGTAGTTAGATTCTATTGTAAATGTAATAGACGGACAATACACTATGTACACGTGGTCATTTTTTACATAATTCTTATATAGTTGCTATTTTGATTTGTGATCTTGTGCCTATAACTTTAATGTTTAATTTTGTTTGATTACAATTTTGTGTCATATTCATATAGGTGTAAAGTTGTTTAAAAATGCCATTTGTACATTATAAAATATTTTTTTTTTTGTTATCTCTGTGTCTCACACTTTATCCAATCCAATCCAGTTATATTTAATGGATATCCATTAA

**FAS:**

>SinsFAS1

AAGACAGTTTCGCAGCAAGTGTCGTTGGCTGAATTGGGCATGGACAGTATGATGGCGGTGGAAATCAAACAGACCCTCGAAAGAGAATTTGAGATCTTCTTAACTGCACAAGACATCAGGACGCTTACATTCGCCAGATTACTAGAACTGACTGAACAACAAGAAGCAGCGGCGTCCACATCGGCGGCTCGTCCAGCCACGGCTGACGCTGCTGGCTTGCGAGTACTCATGAGGAACTTCGGCGATGAAAAACTAGCTTCAGAACCTTTCATCTACATGCCCACTATGGTGTCTGATGGAAATGAGAACGAA

>SinsFAS2

CGCCTCCGCACAGTCGCGCTGCACGTTCGCGACCCGTATGACGGCAGTTTTGTGCGAACACGTTTGCGTGTCGGTCGATACTCGCGATTGCTAATAGGACGTACGCCGACCTTGTAATTACCGCGGGTGTATATTCCACTAGTGGCCGTCTCTCGCTAGTTCGTCGCGACCGCGCACCGCTGAGGATTCCCTAAGGATACCGGCACCCGACCGCAAAACGCTCAAGGAATTGTTTCATTACTCCATTTTACCATAACCGGAACAGAAGCTTGTGAAAGATGCCAGGTGCAGTGAACGGAACGCGCATCAGCGACGATGACGTCGTGCTGACCGGGCTCTCGGGCCGCTTGCCCGAGTCCGACAATATCGAGGAGTTCGCCGAGCAGCTGTTCGCTGGCGTCGACCTCGTCACGGCCGACGACAGACGTTGGACACCAGGATTACACGGACTACCGGAGCGCAATGGCAAACTCAAAGATTTGGTACACTTCGATGCGACATTTTTCGGAGTCCACGCGAAACAAGCACACCTTATGGACCCGCAGCTGAGATTGCTGCTGGAATTGACCCACGAGACCATAGTGGACGCCGGCCTCAACCCGGCCGAACTGCGCGGCTCGCGCACCGGCGTCTACGTCGGCGTCTCCAACTCCGAGACCGAGGAGCTGTGGACCGCCGACCCGGACAAGATCAACGGCTACGCGCTGACCGGGTGCTGCCGCGCCATGTTCCCGAACCGTATCTCCTACACTTTCGACTTGAAAGGACCTTCGTATGCGATCGACACGGCGTGCTCGAGCTCCATGTTCGCGCTCGCGCAGGCCGTGAACGCGATCCGCAGCGGTCACTGCGACGCGGCCGTCGTCGCTGGCACCAATCTCTGCTTGAAGCCGGCAAACTCGCTCAACTTCCACCGGCTGAGCATGTTGTCTCCGGAGGGCCGGTGCGCGGCGTTCGACGCGAGCGGACGCGGGTACGTGCGCTCGGAGGCGGCCGTGGCCGTGCTGCTGCAGAAGCGCGGCGCCGCGCGGCGAGTGTACGCGACGGTGCGCGGCGCGCGCATGAACACCGACGGCAACAAAGTCCAAGGCATCACTTTCCCCGCCGGTGAAATCCAACGTCAACTGGCGGAAGAGACTTTCAATGAGGCGGGACTGCGTCCTCAGGACGTGGTCTACGTCGAAGCGCACGGCACCGGTACTAAGGTGGGCGATCCTCAAGAAGTGAACTCGATCGCTGAATTGTTCTGCAAGGATCGCAAAGACGCACTATTGCTCGGTTCGGTTAAGTCGAACATGGGACATTCGGAGCCGGCGTCCGGGCTGTGCTCCATCGCTAAGATCGTCGTGGCCATCGAAAACAGCGTGATCCCGGGCAACTTGCACTACACGAGCCCCAACCCCGACATCCCCGCTCTCAGTGACGGTAGAATAAAGGTGGTGGATCGTAACACACCTTGGGACGGCGGTCTGGTGGCAGTGAACTCTTTCGGGTTCGGCGGCGCCAATGCACACATAATCCTGGAGACAGAGCGCGGTGGCCGGCCGGCGCCCACGCAGTACGCGGCGCCACGCCTCGTGCTGGCGTCGGGCCGCACCGACGAGGCCGTGCAGGCACTGCTCGAGCACGCCGCGCGCCACGCCGCCGACGCCGAGCTGCACGCGCTCGTTGACGCCGTACACGCGCAGAACATCCCCGGGCACTCGCGGCGCGGCTACGCCGTGCTCACCGACCCACCCGTGCAGGAAGTGCTGGAGGTAGAGAGCGGTGAGCCGCGACCGATATGGTTTGTGTTTTCGGGTATGGGATCGCAGTGGGCGGGCATGGCTCGCACGCTGCAGCGCGTGCCCACATTCGCGGCAAGCATCGCGCGCACGGCGGCCGCGCTGCGCCCACACGGCCTCGACCTGATCAACATCCTCACCGAAGCACCCGATTCCGCCTTCGAGAACGTCATCAACTCATTCGTGTCCATCGCCGCCGTGCAGGTGGCGCTCGTCGACGTTTTGCGCGAAGTCGGCATCCGGCCCGACGGCATCGTCGGACACTCCGTCGGTGAAGTGGGTTGCGCGTACGCGGACGAGACTCTTACTGCGGAGCAGGCCGTGTTGGCGGCATACTGGCGAGGACGGAGCATCGTGGACGCGAAGTTGGCCCCCGGCGCGATGGCCGCGGTGGGGCTCTCGTGGGAGCAGGCGACGGCGCGCTGCCCGGCGGACGTGGTGCCCGCCTGCCACAACGCTAACGACAGCGTCACGGTGTCTGGACCGGTGGCGTCCGTGGAGAAGTTTGTGGCGGAGCTGTCGGCGGAGGGCGTGTTCGCACGGCGCGTAAACAGCTCGGGCGTGGCGTTCCACAGCAAGTACATCGCGGCGGCGGCGCCGTTGCTTCGCAAGAGTCTGGAACAAGTGATCACATCACCCAAGCCGCGTTCGCAGCGCTGGATCTCCTCCTCGCTGCCAAAGGAACAGTGGGACTCCGATCTCGCGAAGTTGAGCGACGCAGCGTACCACGTAAATAACCTGCTGTCGTCGGTGCGGTTCGCGGACGCGGTGCGCTGCATCCCCGAGCGCGCTATCGTGGTGGAGGTGGCGCCGCACGCGCTCCTACAGGCGGTGCTGAAGCGCGCGCTGCCGCAGGCCGCACACGTGGCGCTCGTGCGCCGCGACCATCCCGACTCCGCCGTGCACCTGCTCTCCGCGCTCGGCAAGCTGTACGGCGCCGGTGCGCAGCCGCAGCCGGCCCGCCTGTACCCGCCGGTGCGCTTCCCCGTGTCGCGCGGCACGCCCGGCCTCGGCTCTTTCGTCAAATGGGACCACTCCATCGAGTGGAGCGTCGCGCACTACGGGAACGCGGCGCGCACTGGCGAGAACATCATCGAGTTTGACCTGTCCAAAGCGGACGACGCGTTCATCGGGGGTCACAACATCGACGGCCGCATTCTGTTTCCGGCGACCGGGTACTTGACGCTCGTGTGGCGCACCGTTGCCAAGTTACACAATAAGAAGCCCGAGGAGACGGCTATCGTCCTAGAGAACGTCCAGTTCAGACGAGCCACAATCGTGTCGCGCGACGCCCCCGTGCGCTTCCTGATCAACGTGCTCGACGGCACCGGCGAGTTCGACGTGTGCGAGGGCGGTAGCGTCGCCGTCACCGGCACCGTGCGCCTCGCCGACGACCCGGCGAGCGAGCGGCTGCCGGCCGACGACGCTGCGCCCGTGCCCGAGCCGGACCTGTTGCCGCTCGAGACCGACGACATCTACAAGGAGCTGCGCTTACGCGGGTACAACTACGGCGGCATCTTCCGCGGCATCCGCGCTTCGGACCCGCGGGGCACCGCCGGTACGCTCGCCTGGGACGACAACTGGATCTCGTTCATGGACACGATGTTGCAATTCGGCATCATCGGAGTGGACACGCGCGAGCTGTACCTGCCGACGCGGCTGCAACGCGCGCTAATCGACCCGGCAGCGCACCTGGCGGCGGTGGCGGCGGGTGCCCCCGTGCTGGTGCGCATGCGCCGCGACATCGACGTCATCACGGCCGGCGGCGTCGAATTCCGCGGGGTCAAGACCAGCCTCGCCCCGCGCCGAGCCAATGCACAAGCGGCGCCTAAACTTGAGAAATATACTTTTCTGCCGTACGACAATGCAAGCGTCACGACCGAGGACACGTCCAGGGCGAAGCGCGATGCGCTCACGGTTAGCTTGCAGCTGGTGCTCGAGAACGCGGGCGCGCTACGTCTGAAGGCGGTCGAGGCGGCGCTTGACCGGCCTGCTGAGGCGCTGCTCACGCCGCACGCGCTGCAAGTGCTCGAAACTGAGCCGCAGGTGCGCGTCGAGGCCAGCATCGCCGCGGGTGCCTCCGCCGCACAGTACAATGCCGCCGTGCAAAATCTCGGAGTCAAGGTGACACAAAAAGATGCTCGCAACGCCACGCCGGAGAGCGAATGCCACTTGGTGCTTGCGGCGGACGTGGTTTCGCGCCACGGCGCAATCGCGCTCGTCAATCTCGCCGCAGCTCTCGCGCCCACCGGTTTCGTCCTGCTAGAGGAGCCGCATAAGGCGCTCGACGACCGCAGCGCACAGGAGATGATCGAACGCGCCGGTCTAACGGTGGTGTCGCGACAGGTGGCCGCCACCGGCGAATACGTGCTGCTGAAGCGGCAGCCCGCTGTTCCCGCGGCCCACGTAATCATCGAGGTCGTCGACGACAACGCTTACAAATGGGTAGAACCGCTGCGCGACGCACTCAAGCGCGCCGAGACCGAGGACATCCGCGTATACGCCGTATCGCGTGCGCCACCTAGCGGCGTGCTGGGGCTGGGTACGTGCCTACGCGGCGAGGCCGGCGGTCGCGCGCTTCGCGTGTACCACCTGCCCAACGCCCGCGAACCCTTCTCGCCGAGCGCGCCCGCTTACGCGGCGCAAGTGGCCAAGGATCTCGCCGTGAATGTGCTGCGGGCCGGCGTTTGGGGATCCTACAGACATCTTCTCGTCGGCGATTCGACGGACGCTCTGCTACAGGTGGAGCACGCATACGTAAACACTTTGACGCGCGGTGACCTCGCATCGTTGCGCTGGATCGAGAGCCCACTGAGGTTCGCCCGCGAGCAGCCGCTGCCGGCGACCACCGACCTATGCAACGTGTATTATGCCCCGCTCAACTTCCGCGACATCATGCTCGCGACCGGCAAGCTGCCGCCGGACGCGCTGCCCGGCAACCTCGCCGGCCAGGAGTGCATCCTCGGCCTCGAGTTCAGCGGGCGGTCGTCGTCCGGCAAGCGCGTGATGGGCATGGTCGCCGCGCAGGGTCTCGCGACGACCGTACTCGCCGACAAGGGCTTCCTGTGGGAGGTGCCGGCCGCGTGGTCGCTGGAGGAGGCGGCCACTGTGCCGGTGGCGTACGCGACAGCATACTACGCGCTAGTCGTGCGCGGTCGGCTGACGCGCGGCGAATCCGTGCTGGTGCACGCGGGCACGGGGGGCGTGGGGCAGGCGGCCATCGCCATCGCCCTGCACGCCGGTTGCACCGTCTACACCACGGTTGGCACGCCCGACAAGCGCGCTTTCCTGCGCGAGCGCTTCCCTGCGTTGCCTAACGAGAACATCGGTAACTCGCGAGACACCTCCTTCGAACAGCTGGTCCAGCGCCGCACGCGCGGTCGCGGCGTCGACCTCGTGCTCAATTCGCTCGCCGCCGACAAGCTGCTCGCCTCCGTGCGTTGCCTCGCCATCGGCGGCCGCTTCCTCGAGATCGGCAAACTCGACCTTTCCAACAACACCCCCTTGGGTATGTCTATATTCCTCAAAAACACAACCTTCCATGGCATCCTACTGGACGCTCTGTTCGACGCCGGCAGTGATAACCCCGAGAAGGCGGCGGTGGTCCGCTGCGTGACGGAGGGTATCGCCAGCGGTGCCGTGCGTCCGTTGCCTTCCACCGTGTACTCGGATCAACAGCTCGAGCAAGCCTTTAGATACATGGCGACGGGCAAACACATCGGAAAAGTGCTAATGCGCGTGCGTGAGGAGGAGGCCTCCGGAGCGAGAGCTCCCACCAAGCTCGTGTCCGCCATTCCGCGCACCTACTTCCATCCCGCTAAGAGCTACATTCTAGTCGGCGGTCTCGGCGGCTTCGGTCTCGAGTTGGCGCAGTGGCTCGTCGTCCGTGGAGCGACCAAACTCGTGTTCAACTCGCGCAGCGGCGTGCGCACCGGCTACCAGTCCTGGTGCATCAGAAGATGGCGCGAGCGCGGGGTGCGGGTGGTGGTGTCGACTGCGGACGCGACGACGGCGGCGGGGGCGCGCGCGCTGTTGCGCGAGGCGGCCGCGCTGGGCGCGCCGGGCGGCGTCTTCAACCTGGCTGCGGTGTTGCGCGATGCTTTCCTCGAGAACCAAACGCCGGATGATTTCCGCGCTGTCGCCCGACCTAAGATCGATGGTACGAAGGCATTGGATGCGGCGACTCGGGAACTGGCGCCCGAACTGGACTACTTCGTAGTGTTCTCGTCCGTATCCTGCGGTCGCGGCAACCCCGGCCAGAGCAACTACGGTCTCGCCAACTCAGCCATGGAGCGCATCGTCGAGCAGCGCCAGGCCGACGGGCTGCCCGGTCTCGCCGTGCAGTGGGGCGCTATCGGCGAGGTGGGGCTGATCGTGGATACGATGGGTGGCGACGAAGCGGTGGTAGGCGGCACGGTGCCCCAGCGCATCGCGTCGTGCATGGAGGCGATCGGCGCGCTGATGGCGCTGCCGCACCCCGTGGCCGCCTCAATGGTGCTCGCAGACAAACGGCGCTCGGCCGCCGCACCGCAACAAGACCTCGTGCATGCCGTCGCCAATATTCTCGGCATCAAAGACCCGAGCAAGGTGTCCGATTCGGCGAATCTCGCCGAACTCGGCATGGATTCGTTGATGGGCGCCGAGATCAAGCAGACGTTGGAGCGCGGTTACGACGTGGTTCTCGGCGTACAAGAAATTCGCGCCCTCTCGTTCGCCAAGTTGCGCGAGCTGACCGGCGGCGATACCGGCGGTGATGCGCCTGCGGCCGCCGCCGACGCGAAATCCGACCACTCCGAGGACGAACTCGTCCAATTCCAGACGATCGGCGAACTCGTGCCCAAACAGGTCATCGTGAAACTACCGAGCCAACCGCCCACCGACCCCACCGCGAAACCAGTATTCATGGTGCACCCCATCGAAGGAGTGGTGGACCTCCTGCGCTCGTTGGCAACGAACGTGCGCGGCACGGTGTACGGCCTCCAGTGCACGGCGGCGGCGCCGCTGGAGGACATGACGGCGTTGGCGCGCTTCTACGTGACGCACGTACGCAGCGTGCAGCCGGAACCGCCCTACACGTTGCTCGGCTACTCGTTCGGTGCGAGCGTCGCTTTCGAGATGGCACTGCAATTAGAACAGGAGGGTTGCGCAGCGCGCGTGGTGCTCGTGGACGGCTCGCCGGCCTACGTGGCGACGCACACGACGCGCGGCAAGAACAAGCGCAGCGTGCGCACCCAACAGACGGACGAGGCCGACGCGCTCACGTACTTCGTGCAACTGTTCAAAGACGTCGACGCCCAGAAGGTGTCGAGCGAGCTGGAGAAGCTGCCGAACTGGGAGGCGCGCGTGGCGCAGGCGACGCGCCTGGTGACGGACGCGGCGCCGTTCGCCGAGGACGCACTGGCGGCGGCGGCGACGTCGTTCTACCGCAAACTGGTGGCGGGCGACTCGTACCGGCCGACGGGACGGCTGCGCGCGCCCGTCACGCTGTTCACGGCGCGCGACAACTATGTAACGCTGGGCGCGGACTACGGGCTGGGCGCGGTGTGCTCGGGCCCGCTGGCCACGCGGCAGCTGGCCGGCAACCACCGCACCATCCTGGCCGGCGACGCGGCCGCCACCATCGCCGCGCACCTGTCCGACCTCGTGGCCGAGACGTCCGCGCACTAGCCCCGAGCGACGGACCGTCCGCTTCGCCCGCATTCATTGTGTAGATCTGACGAATGACAGGCGCGGGAAGATCTCGCGTCGTACCAGCTGTATATAAACACCTAACCTGACGTCGCCGAATGAACCAAAGGTTATCGAATTATGAATTCGATCGTATTGTTAAGATTATTATATAACGTTTGTACGTAGGTATATGCAACATGTCGCTATGTGTATTTACATAGTATTTGAATTTTTACGAATTTATTGTAAACACGGTTTTAGACCGAAACGAAAGTGATATCGTGTTTTCCTTTATTGAATCGCGTAATTTGTAAACGGAGGCGGAGTGCGCTCGCAACGCAGGCGCCTTCGACCGTTTTGGCATTTGTATACTCATCACTTAGTTAAGTGTGATAGCCTCTCTCTCTAGTGCCGCGGGGAATGTAGTGTAATATATCTAGAGTAGTCCGCTATGTATTTATTTACATACACATTTATGGTGACGGCGTGCAGTCGGCCGCTTGTGATACGCGACGGTGCGTATCGTACACAGTACACGTGCACGTACACGTACACGTGCCCAGGATGCGCGGCGCGGCGGCGGCGCGTCACTCGTAGTCCTAAGTAGCAGTAAGTAGTTTCTTTGTATGGTTGTTGTGCTAATTTATTTTATCACTCGAACTGAGCCGGCGCGAGCGCCATGTGATTAAACGTGCATTATTTTTCAAAAAAAAAAA

>SinsFAS3

GTCTACTCAAATACTTAAGTAAAGTGATTAAGACGCCGAAACTTCGTAGTGAACGCTGGGTGTCTACGTCAGTGCCAGAGGAGAAATGGAATGAATCTGCAGCTAAATACTCTTCAGCAGAGTATCACACTAACAATTTGTTGAGTCCTGTGCTGTTCGAAGAGACATCGCTATTGATTCCCAGCAATGCTGTGTTAGTGGAGATCGCTCCTCATGGTCTTTTGCAAGCTATACTCAAACGTTCACTACCCACCGGTTGTAGACACGTACCACTTACTAGACGCGGACATGTCGATAATGC

>SinsFAS4

TACGCACAATTCCTCCATGAGAGATGATGAGTTTGACGTTACCCTCAAGTACCTGTGTATCGTACCGGGCTTAGAAGGTCATCACAAGAGATTTAAGGTGTTATGTGAGCGTTTGAAACTACCCGCATTTGTATTACAACCCGGACTCGACAGATTGACTGAATCTATTCAAGACATGGCGCAAAGATATGCCAATGTACTTCTCAAGAAAACTGAATTAAAAAATAATTTTTACATTTTGGGTTATGAAAGCGGAATTTTGGTAACACTAGAGATAGTAGCTATCTTAGAGGATCATGGCTTAACAGGAACAGTGTTCTGCGTTGGTGGCACTCCAGATGAATTCCGAGAGACGCTCGAAGAACAATTAAGAGGAGTTGATACCGAGGAGGCATTACAAGATACCGTTGTGCGTCATATGTATGCACTGATGACTGGTAGAAATTCAGATCATCTCGA

>SinsFAS5

GAAGGATACAATCCCAAAGTTCAAGTGTTATACCCAAAAGTAGAATTTCCCGTATCAACTGGAACACCGCTTTTGTCGCACTTAGTAGAGTGGGCTCATAATGAAAAATGGAATTTGCCGTTATATGTGTCTGCATTTAGAAAGGTTGCGGCAGCTTGTAACTTTGTAATATCTGTTCATGATGAAGAACACACTTACTTACAAGGAAATTTAATTAAAGGAAAAACTTTATTCCCATTTGCGGGCGCTTTAGTTCTTGCCTGGGATACTCTTGCTATGTCTATGGGTATGACGAGAAAGCAAGTGTCGG

**DES:**

>SinsDES1

TTCTCTTCAACATGCTGACACCAACTATCGTGTTCATCGAAGCTATGGCCCAAATTGGCCAAGCCTACGATTTGAAAAGTGTATCCAAAGATATCATAAAGCAAAGATCACAACGTACAGGCGACGGAACACATCAACTGTGGGGATGGGATGATCCAGAATTTACTGAGAAACTGAAGGCGCAGTTAGGTGTTATAAGCTATGAAGACAGAAGTGAAAAAGTTAAAACTGGTTAATGATTCAG

>SinsDES2

ATTCCTTACGCTAATATTGTAAACTTTTAAAATATTAAAAAGCATCTATTTATTGGCCTGACGATTCTGAGCTCGCACTTCACATGCTGCCCCTAGACTGCAGCCTACTGCGCATATATAGCAGGTTTTGATTAGGTATAATAGATTATTTTTGAGACAATGCGCCTCTTTTTTTGATAACAGCTCTGACGAGGGCCGGGGAAGCATGTTTCATGTCATATGCCCATCCAATTAAAGCGAAAAAATCCAAAAACAACGTGGTGACATTGATTGAATATGCTAGTTCAGCAGCTTTGTAGTCCCAAGGGAATGTGTGATGGTAATTGTGCCATCCTTCGCCCATTGCTACAGCAGACACACCCCAGTTTTCGGCTGGCATAATGTGTCTATCATAAGGTTTGTTGCCCCACATGTGAGCGAAGCTGTTGACAGCCCATGTGAAGTTGAGA

>SinsDES3

TCGACTGCACATTCTACAGGTTTTATACTTTTAAACAATGGCACCAAATAACGTCCACGACGAAGTACTGGTGCCAGAAGGGGAGCAACAGTTCGAGAAGCTAGTTGCCCCACAAGCCGCTCCGAGAAAATACAAAATTTTATATGACAATGTCTTATTGTTTTCATTTGCCCATGTGTTAGCTGTTTATGGTTTATACCTCACTGCGACATCTGCTAAATGGGCCACTATTACATTTCAAATTTTCCTGTATATGATTGGACAAATAGGAATAACGGCAGGAGTGCACAGGCTCTGGTCACACAGGGCTTTCAAAGCGAAACTTCCTCTGGAACTCCTTCTTATGATCATGAATTCGATTGCTTACCAAAACACAATTTTCTATTGGGCCCGGGACCACAGGCTTCATCACAAGTACTGCGATACGGACGCAGACCCTCACAATGCGACCCGTGGTTTCTTCTACTCACATATGGGCTGGTTGCTCGTCAAGAAACATCCCGAAGTCATGAAACGAGGAAAAACAATAGACGTCTCCGACCTACGTAACAACAAGATAGTTATGTTCCAGAGAAATAACGGCATACCGCTCATCGGGACGCTATGTTTCTTATTGCCCACGATAATTCCGATGTACTTTTGGAACGAATCTCTTAACAATGCTTGGCACGTCAACTTCCTCCGTTTTGTCTGCAGTACCAACGCCTCGTTCTTAATAAATAGTGTAGCTCATATGTGGGGTTATAAGCCTTATGATAAAAACATATTACCTACTCAAAACATGACTGTGTCTTTTGCTACATTGGGCGAAGGTTTCCACAATTATCACCATGTTTTCCCTTATGATTACCGTGCGGGAGAACT

>SinsDES4

CACATTCCCTTGGGACTACAAAGCAGCTGAATTGACAATTCGTTTTAATCAGTCGGCAAAAATCATTAAGTTTTTTGAGAAGATCGGACTGGCTTACGATTTGAAAACAGCTTCTCCAGAAATGGTGAGAAACAGGATTATTCGAACTGGAGACGGCACTCATTTCAAACTGGGAAATGATAAAGACAAGTCAGCAGTAACCGCCGTTGGCATCTTACACCCCTTGAATCCTAGCTATACTACCACATACAAAGCTCCTGAAACCAATCTCAAACAAGAAGGTCTTCCCCTATACCACGAAAAAGACGTATTTGGCGTAGAATATAGTGATTTTGGGCAACAGAACACAG

>SinsDES5

GCCGGTCAGAATTCAATATTCACTTGGTGTCGCGATCACCGACTCCACCATCGCTACTCTGACACAGATGCCGATCCTCATAATTCGAAGCGCGGTTTCTTTTTCTGTCACATGGGATGGTTGATGATGAAAAAACATCCTTACGTGTTAGAACTTGGAAAGAAAATTGACATGAGTGATATGGAAAGAGATTGGATGGTCATGTTTCAAAAAAAATATTTTTTCCCTTTATATTTTATCTTGGCTGTATTAATCCCGGTTTGGGTACCAGTGCAGTATTTTGGAGAAAACTGGCTTAATTCTTT

>SinsDES6

GTCATACATCAGTCGCCGTTGTCCATACTAAAATGCAAGATCATTGTCAGTTGTGTATTTCCTCGTAACTTTAACACACGTTTGTTTAAGTAGACACGTAACTTCAATACACGTTTGTTTAAGAGACTATGATTTTATGAAGAGCATATAAAGGATCAGCTGTTTCACTGAATAAAACACCGAGATGGCGGATATCAGCGAGACGGGTCCATATCATCCCGAATCAAATGAAGTATCATTGAGCAAGGCGCGGGAAGCTAACTGGCCAGCTGTCCTCTTCTTTATTCATATACATCTGCTCTCCCTTTATGGACTATGGCTTTTGTTCTTCGAACTGAAGTTAATGACAGTTCTATTCTTAATTATCCTAACTTTCTGCGGCATCTTGGGAGTGACAGTGGGTGCTCACAGACTGTGGGCACACAACTCTTATAAAGCGTCCACTGGCCTTCGCATAATGCTTATGTTATTCCAAACACTAGCGGGACAGGGTTCAATATATGATTGGGTTCAATATCACCGATTACACCACGCACACTTCGGCACAGATGATGATCCCTTCAACTATAACAAAGGCTTTCTCTACGCTCACATTATTACACGTCTGAGAAAGTTAAGTCCTCATCAGGAAAAATTGAAAGATGCTATTGACATGTCGGATCTCCAACGAGACGCTGTCGTTATGTTTCAAAAGAGATTTTATTGGATCCTATATGGCATCGTTTTCCTATTGCTGCCACTGAACGCGCCCCTCGAGTACTGGGAGGACACTGTACTGAGCTCTGTGTTCGTGATCGGATTCGTGCGGTATGGCGCCGTGTTGCATGCCGCATGGCTCGTGGAGAGTAGCATATGTATATGGGGGCTCAAACCCGGACAAAAGTACCCGGCAGACTCTAATGCGGTGTTCGTGCTTTCTCAAACATTTTGGCCGCATTACCATTATCTAGTGCCATACGACTATAAATCTGGAGAATATGGCAGTTATGACAGTGGATGCGCAAGCGCTTTCATACGAGTATTTGCAGCGTTGGGTCTAGCCACCAACCTCCGTACCGTGGAAACGGAGACTGTTCAAAAGGCTCTAGCTGAAACTGCTAAAACTAAGCGGCCAATACAAATCTGCTTAGATGAAGCCGCTAGGAGACAGCATCTGCCAGACGATCACTATCTCAGAAGAGGATGAAATTGAATACTTACTTAATCATTTAGAGCTTACACTGTCTCAACAATGAAAAGCTATTTATAATTATACAAAAATGTGGTATTAAAAAAAAA

>SinsDES7

TTACAAGGTTTCTTTAATTATTTTATTTTAAATCGTTTTACAATAATTACATCTACTTACCTATAATGAGATTTATTTAAACTTACATGTAGAATCAACATATACAAGTAGTAGTAAAACATTTGGCTGAATCTAACTAACACTCTTTTCATTACCTTCGTCTAGCGACATAGGAAATTGTACAGGGCTACCGTCACCAAGTCTGTTTGCTATAGAGTTTATCATAACAGGAGTGGCTTCACGTAAGTCGTATGCCCAGCCTATTCGTTCAAAGAATTTAATAAACGTGGCACCGAAATTGAAAATGTCAAAATGTTCAGCCGCTTTATAGTCAAATGGGAAAGCGTGGTGATAATTGTGCCAGCCTTCACCGAGACTACAGGTAGCGACGAAACGATTTTCTGCCGGCATGATATCTTTGTTGTAAGGTTTGTAACCGAAGGCGTGTGCGAGGCTGTTGACAGTCAGCTCGCTATGGAAAACGGTTAGGAATCTTATGAAGCACTGCCACGTTACTGCGCAATGCCATTTTTCATCCCAGAGCCAAATACTCAATGTTGTGGGTAAAATATAGCAAAACATCAACTTGAAGTAGTTGAAATACCTGTTATAGAATTTTAGTAAAGGATCATCTACTATATCGCTCATGTCTATTTGTTTACCGCGTTTTATTACTTCATCATTCTTCTTCATCATAAGCCATCCAATATGCGAGAAGAATAAACCACGGTTAGCATTATGAGGATCAGCATCTGTGTCACTGTATTTATGGTGCACGCGGTGATCGCGTACCCATTGTTCTAAGGAATTCTGTCCAGCACTTGAGAAGCACATAAGCAGTAACAGCTTTAATGGTAACTTGGCTTTGTAAGCTCTGTGTGTCCAAAGTCTGTGCGCTCCACCAGTGACACCAAATCCACTTGCTACGTACATAATACCAGCAAATACAACAGTTTGCCATTTCACAGGGAAAGTGTAATAGTAACTCCAATAAACCCCGACTACATGATAAAGAGAGATTAGTATGAAATTTGTCCATTTTATATCGTTTTTGAAACCCAAACTTTTCTCGAACTCCCTAAGTGTGGCAGATATCGATGTTGGATGCTTTATTTTTATATTACACATATTTTTTTTTCTAATGTAACACTTTTTTTATTTTAATTATGAATATCTCAAGAGCGTTCCAGCATGTAGCCG

>SinsDES8

TTTTTTTTTTTGATGGTTTAAATATTTTATTTTCTAAATCATGTAAAAATTATTATTATTGATCTAAAAATACCTAAATTTTAGTAGTACATAATAGTATATTTTACTAAATTCACTTATCGTTGCTAAGCAACAAATTCATTTTTAACAATAATAAAGCCAACGTTCTGACCATTCAATTCAATTACTTTTTGTGTTCAATCAAAAGAATTGAACGTAGGAACATTTTTGTTTTCAATTAATACATCAATAAAAAAATATTTGTCCCAAAATATTGACGATTGAATAGAATAGTCAGGTTCAGCTTTTATCATTATGCTTACGTTTATGGAAAATATCAATCGATAAATGAACCGCGGAGTTAAAAAAGCAAAATATTTCTTAAATTCGTAATACAATTTTGATCAAAAATCTGCTTAGGTATGAATCTTATACTTAATAAATAAGGAGAATACGAATATTATTAATACAATAATACATACACGATCATTCTAATAAATAATTAGAGCGCAATTATACTTTGTCGTTTAGCCGAAATCGAGAGAAATGCGGGCTAACAATCACATTTCATTAATTTATCACTAATTAAACGTGGTCGTTAAAAGAATACGGGTAAGCCCGGGCGAAAAATCCGCTTTTTTTTTGTGAATAATGGGCTGTGGTTGGCCTGAGAGGCCAACCACAGTCTTAGGTGATGATAAGTGTCGACAGCGCCGAAGATGGCACGCACTGGCCTAACAAAAAAAATGAATTCGGGTAAAACGACCAAGTATACCTGCGCCATTAGATTGATGAAAACAATGAAATAGATCAAAGTCACTAAATAAACAGCTAAATCGTGCAAAATATCTTCTTTTTAAACAAGATCAAATAGATTTCAAAAATATAAAATAGTACACGGTATTTACATTAATTGGCTCAAATAACTTGTATTCAGTTCAAAACGTAATAAAAACCCAAATATGACGTTACATGCGTAACATAAAAGGAGAGCTGTCCATTTAACGACCTTGGAATAATCGCTTAAAGCATATTTTTTTGGATAAAAAAACGTCCCATCACCAACTCTTATCAATAGCACGATATTTATCATTATACGCGGTACCATCACCCTTTTCGTTAATGAACTTTTTGATATGCTCTTCGGAAGCGAACCTTAGGTCATAAATAAGACCTAGTTTGGCCAAAATGTTGAGGAAAGCCGTGGTTGGATTAAAGAAGAATGCGGAGAATTCTGAAGCTCTATAATCCCATGGGAAGGCGTGATGGAAATTATGCCAGCCCTCTCCGATGGTCAGGAGACTGATCAAATAATTCTCCGTCGGCAGGATGTTCCTATCATAAGTGCGATATCCGTAAGCATGAGCGAAGCTGTTGACGGATGCCGTGATGTGTATCCCGATCGGATATCGGATACACACTTGGGATATAAACGCCCAAGTCAAGCTCTGTCCCCAGAAGTACCACGGGATCAACGTGGGGATGACGGCCACGCACAATGTTTGCATCAGAGCGTAATTGTTGTCAAAGAACTGCACAACAGGATCGGCCAAGACATCATCAACGCTAATCTTCTTCCTACAGTCATCGACTTCCTTCTTCTCCTCCAGCATAAGCCAGCCGATGTGGCTGAAGAAGAATCCTCGCTTGGCATTGTGTGGATCACCGTCGGTGTCCGCGTATTTGTGATGGATGCGGTGCTGGCGAGACCATTGGCGGATTTTCATCTGTCCAGAAGCATAGAAGAACATGGCAAGCATGATGCGTACGGGCAGCGTCGCTTTGTACGTGCGATGACTCCAGAAGCGGTGAATGCCGGCCGTCACTCCCATGATTGTCACATAGGTCATGAAAAATGCCCAAGCGACGAGCACTGGGTGTTGCCGGTACGGGAACGCGAACAGAAAATAGAAAAGCGATATATGCATAACTAACAATATTGCGAACTGTACTGGTACGATTCTTGTTTCAAATTGATACCATTTCAATTTTTTCTTTTCGGTATCACTTTTTAAATTCACATCACTACTATTGCCGATCGTCTCCACGATTTCTTCGGTGACGGTGGAACACTCGGAGCTTGGCGCCATTTTGTGTTATCTTTTCTTTTTTTTAATTGTTAATTATTGTTTAATTTATCCACTCTCAGCTTACACAGCCTTTAGGAGATCACTTTACGATTCTACACTGGTTGTTATGTTATTACACTACACTTTACAACTATTGTTAGATATGCTAAATTAAAATTAATATTAGACTATTCGGATGACATCCTGCCGCGGGAATGCGTCCAATAGTCAATAGTCCATTACCCGCTGCCCGCTGGTGGGGTTCGCGCTTACCGACAGTGATGCCAACATAACAATATTGTACCAAATAGCCAAATACAATATCACTTGCAATGGCAGCACAAAGTGCATATTATATACTGGCATGGCAGTCTTTGGGTTTTCGTACACACGGGCGCTTTTTCGACGCTAAGTAACGTGACACGTGATCT

>SinsDES9

AGATATTGTCAATTTTTAGGAAAAAAGTTGATGCTTGGCAATTTTTCGCGCGTTAATATTATTTAAGTACACATTCTCAAATAGTGTCTACTATACAATTATAATAATAAAATAATACTACACGCATAAAAAAACACAACATGGCTTTTTATTATTTATCATAACAATTTGGTATATAAAATATCTATTTTATTATACATTATGTATTCATGCTCTATAATAAGGTACCGTTTGTTGTCTTGAAAATATTATCTTCAATATTCACTGGTTAGTAAGTAATCGATATTATAATATTATTAAATATGTAGGCATCTTCCTCTCTCCTCTATCACGTTGGGTTACATTACCGCATTTTTAAAGCATATTTATTTCTTTGTTATGACAGGATTCACATATCTCTTATTTTTCTATCCATTAAGTGATAAGGCATGAGCAGCCACAATTAATATTTAATGTGCGTTTAGAAAAATATGTAGGTACACAAATACCTAATGATATTTGTCAATTTCTAACTTTAAAAAGTAGTTACAATTATAAGTATATAATATAATAATCTATGAAACACGAGTTCTGTGTGTGTCAATATTTATGACGGTCCTATATATTATTATATGTATTTCTGTCTCTTTAGTTGCACTCATCATAGAATATACTTATGACGCTAAGCACACAAATTGGGCAGTCAGAGGCGAATATAATCTGATCGAGTGACCGGTCATCATAGCCAAATATTTATGAGCTGTCAAGCTGTCTTATAGGTTTATGGTCGATCACGTGTATGTATGTGACATCAGAATATAAATGTGCTATCCATCTTATATAAAAATACATTGTAATTATTCGAAATAATTATAAATAATATAATTGATTAAGTGATTAAAATAAATTATTAGACCATATTTAATATGGTCAAATATTTTTGTTGGAAGCCTATGTTATATTTATTATGGTAAGACATTCTGTAAGTAGATATTATTTGGTTAAAAATCAGATAAACTTTACATTTAATAATAATATTAATATTTAACAATAACTGCTTGGTTTTTGTTTAGAAATAATAAACAAGAAATTAATGACTAAGAAAGATTACAGAATTTAATCACTAATAATTTAATCATAACGGAATTTAGACGAAACCCTGAAACTTTAATGGAATCAAAACGTTATCGTTCGATATTATATCGAACGGAATATAATCGAATGTTTCACACAGAATGTAAGCAACAATTATTATTGACTAGCTTTATGAAACGTAAAGTTTTATGTAACAATGGTGGATTCCAGAGAGAATCTCGTCTGGTAAGTACCTATTATTAAATCAGTTTGAATTTAATTGTTTATCATTTAAGTAAAGGTACACTGGGTTTGGCGAGCGTTGATATTAGTCGATGTGTTCATGGTCTACGGAGTAAGGATCAGGTTCTTCCATCTCTTCAAAAGCACCGTCCCCGCTTCGAATTGCTCTGTTTTTAATCATTGACGAGGTGGCAGCTTTTAGGTCGTAAGCCCATCCGATTTTGGCAAAGATATCGATGAAGTTGGTGGAGATATTTAATCTTCCCAGTTCAGAAGTGCGGTAATCCCACGGGAACACGTGGTGGTAATTGTGCCATCCCTCGCCCAGGGCTGCCAGGCTCACCACCTTATTCTCCACTGGTTTTATGAACCTATCATAAGGTTTGTTGCCATATAGATGAGCGAAGCTATTGACGCTGAAAGCGATGTTCAGAGTAATGGTGAACCTCGTCACGAAGCTTATTACGAAGCTATTAACAAGGGTTTCGCCCCAGAAATACATCGGGATCCACGTTGGCAATATCACATTTAATATAGCGAATAATGGAATGTACAATAGTTTTTGTATTCTCACTACTGGGTCGGCGAGGAGATCGGTGGATGTCTTGCGGAGAGCAATGCGTCGGTCTTCCACAGCGGGATGGGGCGTCAGGAACAGCCAGCCGACGTGGGCAAACCAGAACCCACGGCGCACGTCATGAGGGTCCGCTGCTGTCTCAGTATACTTGTGGTGTACTCTGTGATCTAGAGCCCACGTGTAGATGTCGCGCTGGCCAGTAATAGTGAATAGTAGCGCGAGGATGATACGCAGCGGTGTCCGCGCGCGGTACGCCCGGTGCGACCATAGCCGATGCACGCCCGCCGTGATGCCGAACCCGGACGTGTAGATTGTCGCCAACACGAATAATATTGTGAGGAATTTCACTCTATTAGTTATAACCAGGTACAGTCCATAAATTGTCACTAAATGCAAAGACACTTGAACTATAAGGTCCGGCCACTTGACCCGTGGCTTGAAGTCCATGGCTTCATATTTACTCAAGTCAAAGTCCATATCACTACTAACTTGTTCACTAGCAATGTTTTGTATAACTGCATTATTATTATCACAGTCTTTTGTATCATTTTCGAAGTTATATTTATCATTATCGTCTTCAATTAGTCTCATTTTTATTTGCGAGTGAACTGGCTCATCGCATGCCTCAATGTGCTGCTGCGCAGGCGCCATCCTTGTAACGTCGCGTGACAACGCACAATTATTACAGTTCTGTTTTATAGACAAATTGCATCCGTGCACACTGACATTCGCCTCCGACGAGTGTAACTGTATCTGGAAACACTCGGACGCACAATACGGGAGCGTGTTGTTGCAGCGCGCGACAAACTCGCGCAATCACGCCTACTTACAAAATGCCGAAAGTGTTATTAGGAGACCTTGTAATATAGTCGGTTGCATCAATTCTGTACGACCGCGAAAAGCTGAGCGGGATATTAGTCACGTTTTGAATGTAGCACGAACGTTGTGCACGCGGGCACGTAGCGGCGTGGAGAGAGCACGAAACAGGCGGCGTGCACGGCGTGCTATGGTGGACTGGACTGGTAGTCTAGCTCGTGCTCCGTCGAACTGATGGGATGGGGTCGGGGCCGTCCA

>SinsDES10

CGTCGATGTGACGTTCACAGTTCAACACGTGAATCTTATATGTTTGTGCGTGAGCCCATAGGCACTAATCTCACCGGCTAATCGTAGTAATTATAATTAGATAATAATAAAATTGTATATAAATATAAATTTCTTTACGAATTTGCTTCATTAATTATACAGGTACAGTAACAAAACACACGTACCTACTTGTGTGCTTTTGATTATTATTGAATTCTTTTTTTATTTATATTGGGAGAACAGTGGCAATAAGTATTTTTGTAAAAATTGATCACAAGAAATACAAAAAATGGCTCCAGATGCGACAGACGTGAACGGTGTACTCTTTGAGAGTGATGCTGTAACTCCTGACTTAGCACTTGCTACACCACCTGTAGAACAGGCAGACAATCATCCAAAGAAATTAGTTTGGAGGAACATTATACTGTTTACATATCTGCATCTTGCTGCACTGTATGGTGGATACCTCTTCCTAACATCGGCTAAATGGCAAACAGATGTATTTGCCTATATCCTGTATGTGATGTCTGGCCTGGGGATTACAGCTGGCGCGCATCGCCTGTGGGCTCACAAATCTTACAAAGCTAAATGGCCGCTGCGACTTATTCTTGTCATTTTCAACACTATTGCGTTCCAGGATTCAGCAATCGATTGGTCGCGCGATCACCGCATGCACCACAAGTACTCGGAAACTGACGCGGATCCTCACAATGCGACGCGTGGCTTTTTCTTCTCGCACGTCGGCTGGCTGCTCGTGAAGAAACACCCCGAGCTCAAGAGGAAAGGCAAAGGACTTGATGTCAGTGATTTATACGCTGACCCTATATTACGCTTTCAGAAAAAGTACTACGGTATTCTGATGCCTCTCGCCTGCTTCGTCATGCCTACAGTGATTCCCGTGTACTTCTGGAACGAGACATGGTCGAACGCGTTCTTCGTTGCAACCATGTTCCGTTACGCGTTCTTACTGAACGTCACCTGGTTAGTTAACTCCGCAGCTCACATATGGGGCGGAAAGCCGTACGACAAGAACATTAAGCCAGCGGAAAACATCACCGTTTCGGTGTTTGCTTTCGGTGAAGGTTTCCACAATTACCACCACACATTCCCGTGGGATTATAAAACCGCCGAGCTCGGTAACAACAGGCTTAACTTCACCACGCGTTTCATTAATTTCTTCGCGAAAATCGGTTGGGCTTATGACTTGAAAACGGTTTCCGATGAGATTATTCAGAACAGAGTGAAGAGGACGGGCGACGGCTCACACTACCTCTGGGGCTGGGGAGATAAAGATCATTCCAAAGAAGAAGTCAACGCCGCCATTAGGATTAACCCTAAAGATGATTGATTTTTTTTTAAATAAATTTTACAGACTCCATTTTCATCAACGTTCATAAGTTGAAAACAAAAAGTTAAAATTTAAAAGATCTAATGTTAAAGTATTATAAATACATTATTATTTAAAAGTACATTGTACGCCTGTATCTGTAGATATATGTATCGTTTATTATTTTGTCTATAATGGAGTCTATGCAAAGGTCTATAAAGGACCGAGTATATTTAGGCTCACCGTGGAATGTCGATTGAACCACATAAAAAAAGACATATTTGAAACATAACAATTAGAAGTTAATATATTTTTAACGTTAAATTTATATGTATTTATCTATTTCGTTAAAATATATCTACCCAGTGAGTCTAGGTGTCCCTGAAAATGCACTGAAAATAGTTTTAAGAAAAAGAGAATTGGATAAAGAATGGAAAATATTTGTATGATTGATACACAAGAATGAATATCGGAGAATAATAAAAATGATAATAATTTGCGAGTTAGTCAAACCCTGGTACTCAGTCGGAACAAACTTTAATTATCACTTTCATATAACATGAATATTAATAGAATGATGACAAACATTTTAAGGGTGTGTTCATGAACTACGTAACGCATTTGAGGTCGTGTCTGTTAATGTTTTCTCTATTCCAACCACTAAAACCTTTTTTTCCCTATAATAGTCTTGCACACTATTCATACGAACACCGTACGTCACGGAATCGGGACGAGGGTGTCCCAGCCGAGTATTAAGGTCCTGGTAGGCATGTGATTTGGGGTAAAAAATTGGAGAATATCGCGTTACTTCATTGTTAATGAGCACACCTTAAAGTCGATGGCAAACTGACGTTCTTATGCCCATAATTTTATTATTATATTAGTATGTAGGTTGAAAATGTAGAACAAATCTTTTGAGACTTGACATTGTTCATTCATCTTCAGATTGACGGAACAGATTTTCATTTTAAGATTCAATTTCTCGAAGGTAGCTTGCATATGCGCTGGTATCATCACCTTCGAATCGTAATAATCGGACACTATAGATTTTCTATTTGTGCGTCGTTTTATTCCAAGGTTTAAAATATGACCGTAAGGTCGGCTGTACATTTTTATTGTTTTCATTAGTTTGTGCCAATAACGACATTGTTAGGACCTTATCCGAAGAGAGAGCATATCCTCAATACTGGAATTGAAAAGTTTTTTGCAGACCTCCTTATTCTCTTACCATTGGGACTGAAGATGACTTAAACGGTTTTTATTATTAATCCCTTTTCTCTCTGATGCCATAATGTCTTCTCAACCTATTATTCCTTTAATAATATTTTACCATCATTACAGCAATATCTTGTCTCTCCATATGTGCTATTTTAAAAATAGAAAATTATCCTTTCCTTTAGCAAATGGTTGTGATTTTTAGAAGCAAAGCAGAAAATTCGCGCTTCTTTTTTTATTATTTTCGATAAAAAATATGTAATTAATGTCAAATACAATTTTTCGATCTATATTCTTTAGGTTTCTTTCTATATTATAGGTAATCTTCTTTATGCAGATTATTTTTAATAGATAAGTTTGCGTATAATCTGGCGACAGCAATATCATTTTTATCACAAATCGTATGAAAAACGGCACTTTTTCTATATTATCCGTTAGCATTACCGCGTTGAATTAGCTCGTAGGAGTCTATTTATGTATATGGCATTAAATTCATAGTACGCCTTAGTTTACCCACTTTGGTATAGTGCTTTCAAGAAATTAGCTTTTGCTGTACTTAAGACTGTTTATATACTATTGACTTAAACGAAGCATAAATGTTATTGTTATCTAATAAAGAGATATCCGTGTGGAGGGTACTGATATACTTAGTATTCCAAAATTCTAAATAAAAAAAAGTTGTGCATTTCGAGAAATCGTGCGGACATACATATTGTAGTAAGGATAAATGGATTTTAATAACACATCAGTATTATAAACCACATAAATAAATGTTACATACAGCTGACTAGTCTTGTTGCATTATAAAAAAGTATTAATCTTTTATGTTATAGTAAAAATTATTTATGCTGTAACCTGCTAAGACACTTGCTAATATTTTCTTTGTTATTTAGTAATTATTATTGAAAGTAATGTACACTTGACGTCGTAGTAATTTATTTTTGTAAAATATTAACTTTTAAAAAGTTGAACTTATTGTTAAGGTAATGTGGTATATAAAAATAAATATTCCAAAAAACACATTGTAAATACAAAATACATTAAATAGATAAAAATGAATACCATTCCCGTCAGTTCCCGGGTTTTATGCGGGATTACAATGTGTACGGATATTGACTGATGTGTAAAGGTATATGTGATATCTAGTTGGGTAACGATAATAATTTAAGAGAAAAAAGTTTCCTAATATTTTCCCTGCGAGCGGTCGGTATTATTAATATATATCTTTTTGTACTTGAATTTTATGACTTTGTATAAAGTTGTATTTTGTAATCGTATTCAGATACATAAGTATGATTAATAATTATATTTTTCATGTTGGAAGACGATTACATACTTGCATGTTCACAGTGATTCGATAATAATGTGGCATATTTTTATGTATATTTGCATTTAATTTGTTTTTTTTTCTTCTTACTAATGCCGTGCAGGGAAAATCGAATTTATGTAGAAAAATGAATAAAACAAATTAATATTTACCACCTATATTTCGATAAAGCACAATTTATTGTTAAAAATTACAAGTCATTTAGCAGAATTAGGTATAATATGTACGAATAGATGTCATTATCATTTCATATACTCGGAGCACACATTTCCATTTCATTGTCTAATTTGTTCAACAAATGTTGTCTAAATTTTCTGAGAATATGTTCACTATCTACTATCATATGTTGCTCGATATATTTACATTAAATATATTATGTATGAAAAAATAACGATTTTTTTATTTTTTAAATTACCTTTATTTAAAACGTTGAACGCCATTTAAAATTGTTACCTAATACAATTTTTACTGAATTATACCTAAAATTTCTAATTATTTTCCATAAATTATAAAGTACTTCCCGAAAATATAATATTAGTGATGTACATCGTTTTGTAACGAACGGTTAGAGACATAGGTACTACCTTTATAAAACACGGATACTGTTTTGGGTTGAATCATTTATTTTATTAAACTTTGACAAGCTATAACTTAAAAACTATTAAAAATATTAACATAGTTTAGCCAAATGCAGTACAAAACAGTATATTTGTCATTATGGTTTTGTGATGGTTCATCGCTATTAATATTATAAACCTTTGCAAGTGAGTAAAGATAATTTATTTAATTATTTTTTAAATACATATACAATAGTAACTTAAATCGAAGGAACACCTCTATAAACTTCATTGGTCACGTGTGAATAGTGTGATCGTCTTCTTGACTGAGGATGCCTTTGTCGTCTTCTATTCGCGGATGTCACTTCCACACAAAGAACTATTAGTTTACTATTACACGGGTACATCTCTTGCTCAAGTAGCTTATTACTGTTCAACGGAGAAGCGATGCCGTAATATTTTGGACTGTCGCTCTGCCATTCGTCACAGAAAGCCTTTGGTGAACGAATACCGTGTACATTGCCTCCGTGCCATACTACTTTCTTTGGCCACGTAGGATCAATCAGAACTTTTCTCCCGCTGAAGCTGTAAATGTTCGGTGCTGACTTGGCGAACACGGCTCCTGATCCATTGAACATGTTTGACCAAGAGTCAAATAACAATTCCCCGTGTAGATTCACAACGGGCAAGTTTCGGTCTTTTGTTGTTTTCACTATTGCTTTTAGATCTTCCACTTTATTAGCCAGAAAGGCAACAAAGGTACCGCTTAAGCCCGACCTCCAAGACTGCCTATGACAATCGTAATTGGCGGCATTTCTTCCAGTTCGATTGGTGAAAGAATACATTTTGCCAGAGTAAGGCTCATTTAGTGCAGCCAAACGAATGTAAGTTCTTTCAATTGGAGCCTCCATAATAGGTGCAGCGGTGGATGACGTCATGTGTAGTGGCAATGAATGTTGCGGCGGGCTTGGATATATGTCCCCAGTTTCTAGTAATTTGCCCATCACAACATATTGCCAGCCGTTTTCGACTCTTAACAGTAACGTTTTCTCACGTAGAACATAAGCCAACGTTCCCAAAGGCGTGCTCGAGGTTTTCTTTATGAGTGCTGTTGTCGTTTTATATATCACAGTGCCAGCAGTATAAAAGTCATCTTCATCGCTGGGCTCACCAGATTTGGCATCAGTAAAAGCGTAAAACGTCTTCTGTGCAACGATCCCTCCAGGTTCTCCTTTGGGTCCAGTCACGGAGATGCCAGGTGGTCCAGGTGGACCCGGTGGGCCGGGTGGACCCGGCACTGGGACATACCCGGTGTTGGGACTAGACGCGCCGGGTGCACCGGGTGGACCTGGAGGCCCAGTGTTTCCAGGCGTACCATCGTCTCCTCTGTCTCCCTTATCTCCCTTCAGCTTTTTAATATCGATTACTGGTGCGCTGTCTCCCTTTGCACCTTTATCACCCTGTAATCCTTTTTCACCTCGGAGTCCAGGTGAACCTATAGGTCCAGGAGAGCCAGTGTTACCCTTAGAACCTTTCGGTCCACTTGGGCCGATTGGACCTCTTTTGCCCAAAGGTCCTGTCGGGCCACGAGGACCGTTTCTGCCAGGTGGTCCTCGATCACCTTTAGAACCTTTCATGAGACTTATAGCTTCTGTAGGCACGTTGCCAGGTGGCCCTGGTTCTCCACGATCTCCTTTATGTCCTCTTGGGCCGACTGGTCCCAGGTCCCCTTTGAGTCCTTGCAGACCACGCTCACCGTGAGGACCATGATGTCCATCTTTACCCGGTACTCCTGGAGGACCTATGGGTCCCATATCACCTTTGTCACCCTTTTCTCCAGGTAGCCCCATTTCTCCTTTAAAACCTCTAAGTTTTTCCACTAGAGCTGCACTTTCAGCTGGATCCATATCGGGATCTAGCAATGATGATAGTTTCGCTGGAATGCCTGGAGCACCATGTTCGCCCTTTATTCCTGAATCACCTTTGTCCCCTTTCTCGCCATTTCTACCGTGGACGCCAGGTTCACCCGGCAATCCGTCGCGTCCTGATTCTCCTCTTTCACCCTTTTCTCCTCTCGGACCTTCTATTCCTTTATGACCCCGATCACCCCTTTCTCCCTTGATTCCCTGATGGCCAGTTTCTCCCTTATCGCCTTGAACACCCTTAGGTCCAGGCGGTCCTGGGGGGCCTATTGGTCCTTGATCACCCTTTTCACCAGGCATAGCTACTTCTTTTGCCGCTCCTTATAACCTGGACCAGGAGGCCCGGGCGGACCTACAACTCCTTGTGGCCCTGGTGGTCCTGGTTCTCCGTCCTTTCCTGCTTCACCTTTAGGTCCTAAATCGCCGTCACGTCCTGGTCTACCATCATCACCTCTGTCACCCACCTCACCTTTGAGACCCGGTGGACCTCTTTCGCCTTGCTCTCCTTGTTTACCGGTCATACCAGGTGATCCTTGAGGTCCATCATCACCTCTAGGTCCACGTTCTCCCGGAGGACCTCTCATTTCAGGCATTGTTTTCAATAAGCTTGAAACAAGAGACTCCGAACATTCACATTTCCCTTCTTCTCCTTTATTTCCCGGTAAGCCTATTGGACCAGGAGGACCTTGTGGACCTTGAGCACCAGAAGGTCCAGCTAAATATTCACCTGGTTCGCCTTTACTTCCCTTTTCACCCTTTGGAGGTGGTTCTCTAGTAGGTATGTCAAAATCTCCTTTAAA

>SinsDES11

TATCGACTGGGTATTTTTTGAGAAATTTATCAAAATGATGATTAAGAATTATTTAACCATTTGTTTGTTTTAAGCATTATATTTTCGACGATAGAATAGAATTCAGTGTGTTCTGTTGCGAGTAACGTAATTGGGTTTTATTAATTTTGCCTAACACACCTCCACGAAATTTCATTATTTAGCAACATTTATGTCACTTTTATTCATTTGTTCTAACAGATAAGGATCATCATTATTCTCCAGAATAGCTTTGGCTTCGGCAGATTGGATGAGAGGTGCATCACCATGAAACGTTTCTTCAGTGTATAGTCGGTTGGTTTTCCTCATTGTACCATGTAGCTTATCCCATACATCCATGTTAAAACCGAAATTGCAATGGAAAAATTGATGGTGCTGATCGTGGAACTCAGCGTCAGGCTGCCATGGTTGCCACCACTGAGACTTAAAATTGATGCCTGAGTGGTCGATGATACCGTGGTAGTAATTATATAAAGCCACCATGTAGAATGGTAGCCAATGCACAGGTATGGTAAACAGCAGTAGACAAAAAGTCAGTTGTACGTGCATGATTTCCACTGGATGTATAGCCGTGACAGAAAATGCTGTTGGCTGCTTGTATTTGTGGTGTAACTTGTGGAAGTGCTTATAAAGCCATGGAGTATGGTAGAGACGATGTAGAATATATGTTGTGTAATCCGCATAAATAAAGATGACAGGGAACTGCACGAAGAACCACAGCCATCCGTACTCGTCGAACTGATAGTAAACACTGGAGGGGTTGTTATTGTAGATGTAGCAAGCGAGAAGGGCGGAGTAGGAGCCAGTGAAGAATAAAGAAAGGGATCCGACCAATATCTCGTGGCGTTCCAGTTCGGGAGACAGCCATTTGTCAGGCTGAATTTTCCATTCTG

>SinsDES12

GTAGGAAATAATAACTATTTATTTATCATAATATGATACAGTCTCACAATGAATAATATGGTCCCTCGTCTACCATACTAGGATTTGCTATTATCTATAATATTAGATATGTAGGAAAATCACAGTATAAACCAAACAACATCTACGCCATGTAAATGTAAAACATATCAATTTCCTTTCATTCTAAGATAGTCCTCATGAATTATTTTAAATAAATAATCCCATAAAGCTATATGATCTTCATACGTACATATATTTAATCATTTTTAGCGGGTTCATCTTCAACGTCATTTGATTTATTTTCTCCACTCACTCGTCCCCAAAGATCGCTACCATCTCCAGTGCGTTTTGATCTATTTTTAATCATATCCGGGGATGCAGTCTTCAAGTCGTAGGCCCAACCAATCCAAGCGAAGAAATCGATGAATAAGGTGCTCAAATTAAACTTGTTATTCCCCAGTTCTCCCGCACGGTAATCATAAG

>SinsDES13

GGTGAAACCGCGGGGCACAGCTAATTATATATAAAATTATTAAAAATCGAATACTTAATTCAAAACCACCCTTTAACTCTTACATACCTAAATATTTTGAAAAATTAACTAATGCAAAATATGAGGGTCAATAAGATATGTTACTATACCAAATTTGAAATCGGTAAGTCGCATAGTTTCTGAAAAAATGCGCTGTGACAGACGTACAGGTAAGCGATCTTATACAATATGTTCCGTTCCGTTGTCGGACATACGGAATCCTAATAATAATTAGAGGTGCGTGGCAATATTCCATCATCACAATAAAAAAAAAAGAAATAATATAATCTAGTCGCAAAAGGTATGTGCTTGAAAGAAAAGACGGATACTAAAATATCAGTTTTAATTACTCTTACAGTAAATCAACTCCACGTTTTTAATTTCTTCTTCTGTTTGATCCTTGTCACCCCAACCCCACAAGTTCCTTCCGTCGCCGGTCCTCTGCGATCTGGCGATTATCACATTGTCCGGTATCACTTTCAAATCGTACGCCCATCCGATCCACGCGAAGAAGTTGATGAACAACGTAGCTGCGTTGAACTTAGTGTTGCCTAATTCGGATGCTTTATAGTCGTAGGGGAATACGTGATGGTAATTGTGGAAACACTCACCCGTCGTCAATAGTGAAACTGACAGGTTTTCAACGGCTTTTATGTTCTTATCGAAGGGTCGGTTACCCCATTTGTGTCCGATGCTGTTCACCAGGAAGATCAAGTTGATCGTGAGAACGTGGCGGAGCATCGTCGCTATATGCCAGGAGTTGCTAAAACTCTCACCCCAGAAATACATTGGGACGACCGTCGGTATGACGTAAGCCCACAAGCCAATTACAAAGAATGCATATTTCTTTTGAAATCTCAACAAGGGATCTTTGTAAATATCTTCCATGTAAATAGTTTTGCCTTTCTCCAAAACTTCGGGATGTTTCCTAACGAACAGCCATCCTATTTGCGAGAAGAAGAATCCACGTTTCGAATTGTGAGGATCGGCATCCGTGTCGCTGTATTTGTGATGTAGACGATGATCCCTCACCCAATAGGTGGCCGTGTTCATGAAAGCGATGCTGTTAAAGATCAGCAGAATTATTTGCAGCGGCAATTTCGCCTTATAAGCTTTGTGGCACCAGAGTCTGTGCGCGCCGGCTGTGATGCCAATTTTGGCCGCTTCCAGTAGAAACAGCGACCATATTATTGTAGCCCACTTAGCGGACGTGAAACACAAATATAATCCATATAATGCAGCCAAGTGCATATACGCAAACTTTATCACATTGTGCTTGACGAGTTTGTACTTGTACGGAGCGGGTTTGTCTTCTTCTCTGACCATTATTATAGACTAAACGGCGAGTCTTACAGGCAGTTAGCTATATATAGCTGACAAAGGTTAACGGACCGTGCGGATCGTGGAACGCCTGGAACAGGGTTGCCCGCCGATGAG

>SinsDES14

GAATATGAAGGGAGAGAAACTCTTCTTGTCTAAATCGGGCTTTAGGGTTTTCTTAATCTAGTCAAAAATTATTTTATCAGTTTTCTATTCTCTCTTTCTATGACTAATTTCAACAAGTTGTCTATCTTCGTCGGGCATGTCTTTATCATCCCATCCCCATACGGGGTTCTCCGAATGATGATGATCATGATCGTCAATAAAATCTTCCTTAGTATTGATCGGATGCGATCCATCTCCTGTTCTGAGCACCCTTCGACGTATCATTTCCGCTGACACTGTTTTGAGATCATAAGCCCAACCGTATTTAGCTGCAAAGTCGATGACGGCCGTGGAAAGGTTGGTCCTGTAGTTGCCGAGCTCGGCCGCCTTGTAGTCCCAGGGGAACACGTGGTGGTAGTTGTGCCAGCCCTCGCCCACCGCACAAATCGCCACCGTAACGTTGTCCGTCGCACCTATGCTTTTATCATAGGGCTTGTTGCCCCAAATATGGGCAGCGGAGTTGACGAGCCACGTGAAGTTCAGCGAGACCGTGTACCGCCAGATGGATGCCACGTACCAGGAAGTCCATGGGTCCTCGCCCCATAGCCACACGGGCAACAACGCCGGGAGGACAAAACATAGTATAGGCATTACTACAAGATACGTCTTTTTCTGTAACATGACAATGGGATCTTTTTCGAGATCTGACATGTCAATGGAGACTCCCTTTTCAAACACTTCCTTGTGTTTACGGACCATCAGCCAGCCGATATGTGAAAAGAAGAACCCTCTTCGGGCGTTGTGCGGATCCGCATCCGTCTCCGTGAACTTGTGATGAACTCTATGGTCGCGCACCCATTCATAAATGTGGTTCTGGAAGGCCATGGTCTGCAGAGTAGCCAGTACGAGTCTCAGCGGCCAACGCGCCTTGTAAGCGCGGTGCGCCCAAAGCCGGTGCGCGCCAGCCGTCACGCCCATCGCGGACAATATCGTGAATATCACCGCAAATACGGGTGTCCATAATTTAACATTTCCTGTCAGCATCAAATATAAGCCGTACAAAGCTCCGATGTGTAAGTAAACAAATGCTAATACGTTCCTCCATACGATCTGCCATTCGTAGTCTCGTCTTTTGACCGGCGCGCTGTACGTGCCAATAGTGGGCAGATTGTCGTCGATATTCTTCGCTGGCAACATTTCGGAGAGCACAAAGGACGTTCTTGATATTGACATTTTGCGGCAATACTCCAATGAAAACTCTGAAAAATCGCTGCAATCGATGAAAATTATAGGCTGAATCCAATTATAATTCGATTTCACACACTTATATACATTTCATACACTGGTGATTTAGATTCGCCGGGTTGTGTATAGTTATTATATTAGCAATAGTTAGATTAGATATGAATGGAACGTGCAGCGAGCGCCCGTGACACTGGGCAAGTAGGGCGGCGAG

>SinsDES15

GGCAAGCGTGTGGATCCGGCATTAGATTTAGTCATCATCATGTGTCAACTGTCAGTGTCAGTCGCTTTTATTCGTTGGAGGGATTATTTCATGTTATCATTTTTCATAAATATTTAAAGTTAAATCAGTCTTTCTTGGCGGTGCCATTCGCGTTTCGTTTGGCTAAAATTCCTTCGACCACTAACTAAACGTTTTTGTAATGGTTTTTGTTTTGTATTAATTTTTCGTATTAATTTCTAATTACTTAAGTATACAAAATGGGAGCGCGAGTGTCTAGAACTGATTTTGAGTGGGTTTACACTGAAGAGCCGCATGCTAGCCGAAGGAAAATAATTTTAGAAAAGTATCCCGAAATCAAACAGCTATTTGGCTATGACCCCCTGTTCAAATGGATAGTGACGGCCATGGTGCTAACACAAATAGCAATGATGCCAATCGTGCAGCACATGAGTTGGCCAGTCATGCTGCTAGTTGCATACTGTTTTGGCGGCGTCATCAACCACTCACTTATGCTCGCAATCCATGAGATCGCCCACAATTTGGCTTTCGGTCACAATCGTCCAATGCATAACAGAATATTTGGATTCTTCGCGAACCTCCCTATCGGCATACCCATGTCAATCAGCTTCAAAAAGTATCACTTGGAACACCATAGATACCAAGGCGACGAAGTGATAGATACAGATTTACCGACATTGTTAGAAGCGAAATTGTTTTGCACCACCGGTGGGAAGCTCGTGTGGCTTATATTCCAACCATTCTTCTACGCCGTACGCCCGCTCGTGGTGAGGCCGAAGGATCCTACTCCGCTGGAATTCGTCAATCTAATTATACAATTATTCTTTGATGCAATCGTCGTGAAATTGTTTGGTTGGAAAATATTAGCGTATATGATACTTGGCTCCCTAATGGCTATGGGCGTGCATCCTGTGGCAGGACACTTCATATCGGAACACTACATGTTCCGAAAAGGTTTCGAAACGTATTCGTACTACGGACCGTTAAATTGGATAACATTCAATGTTGGCTATCATAATGAACATCACGACTTCCCAGCGGTGCCTGGCAGAAGATTACCCGAGGTGAAACGTATTGCGGCCGAATTCTACGATGACCTACCCCAACACAAGAGTTGGTCCGGGGTCCTGTACGACTTTGTCACGGACCCGGACATCGGACCCTACGCGCGGATCAAACGCAAACACCACGGCCTGGACAGCTAGACTTTTACACACTGACAATCGACAATACCACTACTGTAATAGAGTTGAAGATGAGCTATTATGATTTATTTATTATTAATTAGATTTCAATAATCGATTTTGAAAAAATTGTAATCGAATGATTTAGGGTACTTGACTCTGTGAAAGGGACAGTTTTGGAGGTCATTTACTACACTTGGGTCTCAAAACTGTCATTCGACAATTATATCTTGTTATTATATATAAATAAACATCAAGTTATGAGGAATTAAAAAGGCCTTGTGTGATTGCATTTGTCAATAAAGGTTACTTGACTTTGCACACTAGCAAAAAATTTTGCTTTTTCTTTTTAAGCGCCAAT

>SinsDES16

GAAAACTTGCACTTATTAGAAAATTGAATACATTATTAATTAAATAATATAATTTATTAATAAAAAAGACATATTAATAATTTTAATTTAAATAAATGCTTCAATCGAAATAGTCGTTGAAAACGTTTGAAAATCATATTGGCTCTTGCTATTATATTCAGCCTTGGCTTTACAACATTTTGTCGTCATCCTTTAAAACTTCTGCCATGTCTTCATCATCCCATCCCCATAGATTCGTGCCGTCACCTGTAAGCTTAACTCTGCTTCTGATGAGCTCATCTGACACAGTTTTCAAATCGTACGCCCAACCGATCCGAGCGAAGAAATCAATGAATTTCGTAGTCGGATTAAACGTGTTACCAAGTTCAGCGCTGCGATAATCCCACGGGAACACGTGGTGATAATTGTGGAAACCTTCTCCGAGAGTGACGATAAAGGTGGCGACTTTATCTGTGGGCAATATGTTCTTGTCGTATGGCTTGTAACCCCAACTGTGAGCTGCACTGGCAACTAGAAATGTCACGTTCAGGTTGCAAACGTAACGAAGCATATTTATGTGCCAAGCGTTATTCAAACTCTCCCCCCAAAAGTACATCGGAATTAAAGTGGGTAACACAAAGCAAACGGCGCCGATGAATGGCACAGCATATTTTGCCTGGAATCTCAAAACAGGATTATCATAAATATCAGACATATCGATGAGTTTGCCTCGTTTCTTGACTTCGGGATGCTTCTTCACAAGGAGCCAACCGACGTGCGAATAGAAGAGGCCACGAGTTGCATTGTGGGGATCAGCATCGGTATCGCTGAACTTGTGGTGCAGCCGGTGGTCTCGCACCCAATTAGTAGCTGTATTTTGAAACGCTATCGAGTTCATCACCATCAGAATAATTTGCAATGGTAACTTTGCCTTATAACTCTTATGTGACCAAAGTCGGTGAGCACCCGCCGTCAATCCTATTGTGGCTACCACAAATAGGAAAAATGCAAACAAAATTGTGGCCCATTTGGCTGACGTAAAGCATAAAAATATGCCATATATTGCAGCTACGTGCCAATATCCAAATGTTATGAGATTTTGATACACAATATCAAATTTTCTTGGTGCAGCTTGTGGTGCCACCAGTTTTTCTAGGCCGTTCACTTCTTGCAAAACTGCGTTATTTTTCTCAGTATTTGGAGCCATTCTTTATATCGCCTAACCGTCGTATGTTTAGTGTGCGTGTGTAGCAATGAAACCTCGGAGTATCCGGACACAAACTAATACGAATAAATCGAAAATTTAAAAATGGATTCCGAACGTTACAATAGAAGGGCTGTTGACCCCTCCTTTATTTAAAACAATCCTGGTTATACACTTTCGACGGTACGTATGAACTGTTCACTTCGAAGCCTGAAATTAGACTGAAGTCTGGATGCGAAACTGCACAGTTTTAAAGGGACACTAGCCTTGGGCCCTATCCCACTTTCGTCTATACTATTTG

>SinsDES17

CTGCGATCTTCACGCAAATACACAGTAGCTTACATGACATATATATGGGTGCGGGCAAATTTCTTCATACAGTCTTTCGCGATTCATATATCTGATCACACATAAATCTTCAAATTAAAAGTTAACTAATATTCGGTAATTTGTTTAAGGATCGGCACATAATTACTCGTATAAGTGACTCTTCAAATCGATATTTTTTCAAAATTAACACGAACTTAGTGGAATACTGCTACTAGACATATAAAAACTCAAAATGCCACCACAAGAGCAATCTACTGCGTCCTGGGTGTTGTACGAAACAGATGCCGCGACTGAAGATAAAGCCGTTTCGACTCCGGTGCCACCTTCGGCTGAAAAAAGAAAATGGCATATAGTATGGAGAAACGTCATACTGTTCATATTCTTGCATCTTGGTGGTCTCTACGGTTCTTATCTTTTCCTGGCAAAAGCTAAATGGGCAACAAGCATATTTGCGGTCGCCTTATACATCGCTTCTGGGTTGGGCATCACTGCTGGCGCGCACAGATTATTTGCTCACAAATCATACAAAGCTCGCCTCCCGTTACGTGTCCTATTGACTATCTTCAACACGATAGCTTTCCAGGATTCCGTGATAGATTGGGCACGTGACCACCGCATGCACCATAAATACTCAGAAACTGATGCCGATCCCCATAATGCAACGCGTGGTTTCTTCTTTTCGCACATCGGCTGGTTGCTCGTAAGGAAGCACCCTGAAATCAAAGCTAAAGGACACACCATTGATATGAGCGACCTTAAAGCTGACCCCGTCCTCCGCTTCCAAAAGAAATACTACTTGCTGTTGATGCCCCTGGCATGCTTCGTTCTTCCCACCTACATTCCAACATTATGGGGCGAATCCCTCTGGAACGCTTTCTTCGTGTGCGCTCTTTTCCGTTACACGTACGTGTTGAACGTTACGTGGCTCGTTAATTCAGCAGCCCACCTGTGGGGAGAAAAGCCATACGACAAAAATATCAACCCCGTCGAGACAAGGCCGGTGTCACTCGTCGTACTTGGTGAAGGTTTCCACAACTACCACCACACTTTCCCTTGGGACTACAAAACCGCCGAATTAGGAAACTATTCCCTAAACTTATCGAAACTGTTCATCGATATTATGGCTAAAATTGGATGGGCTTATGACCTAAAGACCGTGTCACGTGATGTCATTGAGAAACGTGTGAAAAGGACGGGCGACGGTTCCCACCCCGTTTGGGGCTGGGACGACAAGGATGTTCCCATTGAAGAGAAAAATGAAGCCAAAGTAATAAATCCAGAGAAGACTGAATAAACCAAACGCCTGATACAAAGGTTAGGGTAGGGTTCACTTAAACGACCATGTCCTTAGATATGTAGGAGCTTAGGTACATTTACGTACGTGGAGATGTGACATCTCCACTACTATGTTACGTACATATTACATATGTATATATCGTAGAACACGATTGCGACACTTCTCATTCCTACATATATTGTTCTTACACAGGTATTGATATTTATATATATATATTTAATAATGAAACCATTATTAGAAGTACTGTTGGAACACTATATTTTATAAGACTAGAATTTCATCGCATTGGTGAAATATGTTATCAAATTAAATTACTTCGATAACTTTTAATTGTTAATGTAATAATGTATCTTACCCTATAAGTGAAGCAATATTTTGTGATCAATCAATACACAGACATAATTATTTTAAAAAAGGCAGATATAAAAATACATAGACTTGTACTTAGATATAAATATTGTTGTGAATACTCTTTGCCTTAACATCCGTTACGTAGGTATATTATATATTGTAAATGTATTCATCTATCGGAAAATATGAATTTCCTTACATAATATGTATTTGTGATGAAAGTACCTATGTATAATCTATACATACTTGTCATTAGTTCTATTATTATCAACTTGTCTTTGATAAATAATATAATTGATAAAAATTATAATTAGATGAGTATTGATTGCTGTATAACGTCAAATTATAGCATTCGTAGAATGTAAATATTAATCATTTTTGGCTTTATGTAGTCGTTTAAAACATTATGTATAGTATGTTTAGTTCCACTAAACATGTGTAGGTATGTGGTAAATTACATTTTATAAATTATTTTTGTAATAAGATAGCTTTTAGTTCCTAATATTTATATTGTAGTTTTAAATAAATCTAGCATAACAAAATAACAAAAAAAAAA

**ACO:**

>SinsACO1

GCTGGGAAAGTCTCAGCATGTGTGAAAAGTATCAGAAAACGGATGTCGTCCGGATTGAGTTATGAAGACGCATGGGAATTGACCACTGTGCAATTGGTGGCTGCAGCTGAGAGTCATTGCCGTGCAGTCATATACAACACGTATTGGAACGAAACCGTCCGTCTGACCGCTATGAGTTCCCCCAACGTGAGGGCCGTGTGCGAACAGTTAGTGCGGCTTTACTTGTATTACTGGACTTTGGAGCGGACCGGGGACTTATTGAGGTATTCGTCAATAAGCGAAGAAGATATCGTCAGACTGCAGCAGAAGTACGAAGAACTATTGGCTTTAATAAGACCCAATGCTGTTGGACTGGTCGATGCATTTGATATTAGAGATGAGATCCTGCAGTCGACGCTAGGCGCG

>SinsACO2

TTTTTTTAAGAAAATATAAAAAACTTTATTGTAAACAACATTTAGGTACATCAATCTCATAATATAATCTCTACACTGGTTAAACATTCCAAAACGTAATACTGAGAGATCATGCATTTCAAAAAGGAATGTTTTACATCCACCACCTAACAATATAAACATTATTAAAATACATAATCATTTATTATAAAATTTTTGATCAGCTCCATTTAATAATATAAAAAAAAATGTCTTAATATAAAATAGTTGATTGCCACACCCTCTTGTTATCATTTTTCTTAAAACTAATTTGACATCAGATTTATTAGATCACAGATCACAGTTTCCCTTGCATGAATGGCTTGAGATATTGATGGAAACTCTTGTTCACCGGTTCAGCATTGAGCGGACTCTTGAGCGCGTCCGCCATCAGGCGCTCGTACACGCGCCCGTCGTACGCGCCCAGCGCCGAGTGTAAAATCTCGTCCCTTATATCGAACGCATCGACGAGTCCAACGGCGTTGGGTCTCAGTTTCGTCAACAGCTCCTCGTACCAGTTCTGGAGCTGTCCGATGTCCCGCTCCGATATCGACGTGAACCTGAGCAAGTCTCCCACGCAGCGTAGCGCCCAGTAAACGACGTACAGGTCCACCAGCTGCAGCAGCACCTGCCGGAGCGCGGGCGACGCGCGCTTCGCCTGCGCCTCCGTCTCCTCGTAGTACGTGGACAATACGATCGCGCGACAGTGAGCCTCTGAAGCGGAGGCCAGCTGTACAGACGTCATGTTCCACGCGTCCTCGTACGACATGCCAGTCTTCTGTCGCTTCTCGATGTTGGCAACGCACAGGCCAATTTTACCGGCAGCGACGATGTGGAACCCGCGTATGATGCCCTGTATGGAGTTGTCCCAGGGCGGCGAGCGACGCGCGGCCGCTTCGCGCGCGATGTACGCCACCGTCGGCGGCAGCGCGCCGCTGCCCAGCGACTGCTGCCACGCCTTCACCAGGTACCTGGCGGTCTGCAAGAGCAGCACAGTGTTCTCCCCCTCGTAGGTGCAGGCGGCGGTGACCAGCCCGTAGGTGAGCGGCAGGTTGGAGGCCAGCATGTACCCGTGGCCGCCGCACGCCAGCCGACACCGCTCCACGCACTCCGCCGCGTCCGCTGTGCTCACCGCTTTCAGGCAACACGACAAAGCGTGTAGCTCAGGCAGTCGATCCATGTCGCCGGCGTCCAGTTCGGCGGTGACGTTGTTGTACATGTTCCACAGCCAGTTGGCGCCGAGCCGGTACGCGTGCACCGTGGCGATGCCGATGAACAGCTTGTGCTGCTGGGTCATGTAGTCCAGGATCTGGGGCTCCGGCTCATTTGGTTTCGGTTGCGACTGCCTCCTCACTGCGGAGTATCTGGTGGCGATGGTGACCGCCTTCGCCATGTAGTTGCACATGTCGTTCACCAGAACTACGCGCACAAACATCATGGTGCCGTACGTCAGCTTCGAGCTCGGCGAGTTCACATATGTGCCGTCCTCTAAAACTTTGGAATTCTTCATAAGCATATTTTCCCTCGGTATTCTGACACGATCGAACCCCAAAAAGCCATTATTGGTGCCGTTCATGCCGAGTTTGGCGCCGATTTCGCCGATTTTAATCCCCGGAAGTGGCTCGTGTGTCTCTTCGTCACGCAACTGCACGATGAACGGATGGATGCCATGGCATTGCCCTTTCGTGTACAGTTGGGCCACTACGATGCAGTAGTTCGCTGTGTGTCCCAGTCCGCCGGGCCACCACTTGTAGGCGGTGAGCGTGGGGCTGTGCAGCACGAACTCGCGCGACGCCGGCTCGTACGTCGCCGTCGTCTCCAGCCCGCGGATGAACGTGCCGTGGCCCAGCTCCGTCTGAGCGTAGGTCCCGATAATATTGCAGCTGAACGCCCTGCCGATCCAGTAGGCCTGCTGCTCGACGGTGGCCTGCCCCATCAGCGTCGGAATGAACATCACGTAGTGCAGCGTCAGCGGCGAGCCGTCCGGCAGGATGGCCGAGCCCAGGTAGCCGCCTAGCACTTCCCTATAATTCTCCATGCCAGTGTTTTCTTCTTCCTGCAATCTTCGTATGAGCTTGAACAGCAAGCAGGCTTTTTTGACAGCCAATTCATATTTCTCTTTGTGGCTGAGGTACTCTGAAGGTACATCTTCGAGGTGAATGCCCTCTCTGAGCACTACCTCCTCTCGTTTTTTCCTCTCCTCGGTTTTGTCAACACCACCATCGATTACATTCGTCAATTCTATTGAATTAAATGTACATTTATCCCTTTCTTTTTGCAAATCAGGATTCACTTTTATAAGTTCTTTCATATTCGCAAATAAATCACAACTTCTTATCAAACAACTTAAACTGACAAAATAACTTTCATATATATAATCAATTATACTCTATGCAAAATAACTATTTGGCGATTGGTCTCTGAATAAAAAAAAAGTAAAAAAAGAACGTCTGATGTGACAG

>SinsACO3

AGATTGTCGAGTATTCTCTACCGAGTTGGGGCGTGTGCCGTATTTTCATACATTTTTACTGAAATAAACCTTTAAATAAATTATCAGTTTAGCTAGAAACTACAAAAAATACTACGATTTTTATCTTTTTAGTTTCTTCTAACGTTCCATTTCAAGAGGCATGGAGGCGCTTAATTTTTTGCCGGACCCACCGAGCGGACCGCTAGATGTGTACAGAAAGGCAGCTACGTTCGAGTGGAAAAAATTGAAACTAGCATTGGAGGGAGATATTAATTTGTTGAAATTAAAGTACCATATATGGCATATACTGGAGAAAGATCCTCTGTTTGCACACAATCAGGTCACTCCACCCGTGGAGGAGCAGAAACGTATCACTCAGCTTCAACTGAAGAAGATTAACGAATACAAATTTATAACCGATGAGATGTTTAATTTGAGTTATAGTAAAAAAACTCGAGCCATAATGACAATAAACGAAGCAATCCAATCGCTCAATCCTAGTGTATCAGTGAAAATGGCTATTGGAATCTATCTTTTCTCAAATGCACTTCTGTCTCTTGGTACAGAACGACATATTAAGTTTTATGAAGACACCATTAAGAATAGAAAGTATCTGGCCAGTTTCGCGCTAACGGAGATCGCGCACGGTTCGGACGCACGGCTCATGCGCACCACTGCCACGTACGACCCCGAACACCGGCAGTTCGTCATCCACACACCAGACTTCGAAGCCGCTAAATGCTGGGTTGGTAATCTAGGCAGAACGTGCACCCATACACTGCTCTTCGCCCAGCTAATAACTCCCGATGGCACCAACCACGGTTTACATGGATTCGTTGTGCCCATACGCGACCCTGAAACATTGGAAACTTATCCTGGACTCATTGTCGGAGACATGGGAGAGAAGATCGGCGTTAATGGAATTGATAATGGATTTATAATGTTCGATCAATATCGGATACCTCGCGAGAATCTTCTAAATAGGACCGCTGACGTCACAGAGGATGGTGTCTACGAGAGTTCCTTCTCAGATCCTGGCAGGATATTTGGAGCAGCGCTCGAAAACTTGTCTGCAGGTCGCATCGGAATAATGCAAGAAAGCTGTCACTCGTTAGCGAGTGCGGTGACAATAGCCGTGCGATACGCGGCCACCAGGAGACAGTTCGGTGATAGAGACAACGAGATACCACTGCTGGAATACCAATTGCATCAATGGCGTCTTTTCGGTTACGTGGCTGCTGCGGTCGTGTTTCGTGTATACATCGAGAGTTTTACCCAGGTGTACCTGAACATTGTTGAAAAATCCAACGCTGGTCTCAAAGTCGACAATCTGAGTGACACGGTGTCAGAGATCCATGCAATGGTATCCAGTAGCAAGCCGATGATCTCATGGACAGTTATGGACGCGGCGCAGGAGTGTCGCGAGGCTTGTGGGGGACATGGTTATTTGAAGATAGCGAATCTAGGTGAAATACGCAGTAACCACGAACCGACAATCACATATGAGGGCGATAATAATGTATTGTCGCAGCAAGCGGGCAACTGGCTACTGAGGCAATACGAGGCGGCTGTGACTCGAGGCGAAGTCGCCTCGCCTCTCGGCACCATCGCCTTCCTCGCTGACCACAAGCAGATACTTAGCAACACTTTCCAGGGGACTGAGACCAGGGAACTGAAGACCCCCGAATTTATAATATCGACCTACAAATGGCTACTTTGCTGGCTACTAAAACAAACACACGAGAAATACGAATCGGAAATCGCCAAAGGCCTCACGAAATTCCAAGCCAAGACGAAATCACAGGTGTACAGGTGGAAAATACTCACGCAAGTGTACGCGGAGTACTTAGCGGTTATCTTCTCTCTGGAATCCATAGGCAAGAAAGAGGAGTCGTTGAAGCCCGTTCTATATAAATTGTTCACTCTGTATAGTCTATGGTCGCTCGATAAGCACTTAGTTGAACTATATCAAGGTGGTTATGCAAAAGGCGAAAGTATAGCGAGATTAATCAGAGATGCCATATTGGAGTTGTGTGAAGATTTGAAAGGAGAAATCGTGGGAGTGGTCGACGCTTTGGCACCGACGGACTTTGTGCTGAACTCTGTTCTGGGGAAGAGTGATGGTAATTTATACAAAAATCTCCAAAAAGCCTTATTCCACACTCCCGGTGCGATGCAACGCGCCTCGTGGTGGCGTGACGTTGTCGATGTACCTCGGTCTAAGCTGTAAGTCTAGATCGAATTTAGCAAAATGTCCTAGGTAATATCCGTATCTTCTAGGTGAATTTTATTGTTGCATAGGCGCGTCCAGTATTATGACAAAGATAAAAGGGGGGGGGGGGCAATAAATAATTTCTACAGAACCCTATTCATATGAAGTACAGATATTCAGTTCAAAACCACGTATATTGAGTATAATTGTAAGTTTAGAGGGGTCATTGACCTCTCCTCCCCCCTTGTCTCTCATATTTTTCTCTTTCTTCGGATACGCCTGTTCAGTTTTATGTTATTGTTGCTAACATCGTATGTGTTAGTAAGAACAGTGAGTATACGTCTCATGCCGTGCGTAATAGAACAGAATTTAGGTTTGTCGCCCAGTAGGGATTGTTATCCAAAGTAAATACTGAGCGCTCTACACACCGAACCCGCCAACACAGCCCGGCGGCTCGACATGAGCAACTGTCTTGGCAGTTTCGTCGTTACACTTTGGATATCATCGGCGACTACAACTGAGCGCGTGCGTAAGTCGCCGACATGCCGCCATTATGTCGCCGGGTAACACGATCGGGTTC

>SinsACO4

AATTTCTATTCCGTTTAACAAATATGTAAATTTCGGTTTTAGAATTGATAAAATGTCAACCGGTACAATTATAAGGTTACAAATGGTTAAAAGAAGGCAAGTTTGTTCCCAATATTCGAGGAAGTAAAAAAACTTAAAATCGAAATAGTAAAAATATAAAAATATTTATAGCGCTACACAAGTGATTGCCATAAAATACAAAACCTAGGCACCCTAAAATATAAAACAAAAAACAATCCGTTTATTTCCAATTATCGAGCAGTCGATAACATCCAAAAACACTTTAAAAGTATGAGGTATTATTACACAGTACTTCAATTCAGAATATTATGCAGTGTCAGTGAATGTGAACAGAATACCGGATATAACCTTCACTACCTCGTCGCTACTTGTTTAAAATTGAGTTTCAGGCTTCTTACACTTTGCTCGCAACAAACAAACGACTAAACAAACATATCTCTACCAACGAATTTATATTCAATTGGCAATTCTTTTCTAATGTCTATTAAGTTTGTTTTCTAGAAGATAAGGTAAAAAAAAAAACTCCCTTCCCCCCTGCCCGAAGACCTGGATCCTCTCGTTTAGAGTTCCCAACGTGAGTAATTAAAGAAAATTATTATAGAATTTGATTATAATCAACTCATTCATAATTTATATTTTATTTATAACCAGGGCCGACACGTCCCTATAGGCAAATAAGGCGACGGCCTAGGGCGCTCGAATTTGGCTAGCGGCATAACGAGGAAATTGTTTACACTTTTTTTTAAATCAAGGCGAGGAAAGTGAAAATTATATAATCGGGTCAATCGAGTGCTTTCATAAAAAAAATATTGTTTTAGGTACATTAATGTTGTATTCTGTTGCATATTTTTTGTATAAAGGCAGAGGGCGCCCAGTTTTCTCGTGCCGATGTTTATAACTTGGATGGAATGTAAGATTGCCAGTAGACGACGTCTCGCCACCAGTCCGGTCTAGTCATGGCGTCAGGAGATGTCATCACGCTGTTCTGTATGTGTTTGTAGACCTCTCCATCAGCTCTGCCTAGTACTGACTTTAAGCACAAATCCGGGGGTGCCAGTGTGTCGACCAAAGCCACGGCTTCGGGTGCCAATTGCGAACACAATTTTAGTATTCCTTCACGGAGAAGTAGGCCTTGGTTGCTGGTAAAGAAACCACCAATATATAAAGTTGCAAGATGTTTCTCCAATATGAAAGCGCCGTATAAAGCGACCAATTTCAGTAAAATGTTTCTACACGAAGCGTCTTCAAATCTACAAGCTGTTTTGTAGAAATGTTGTAGAATGAAATTCTCTCCATAGACAATAGATAAAGTGACAGCATTGAAGGATTGTGATTCATTCTTTGCTTGGAAATTGTCTTTGCCTTCGCTCTTGAGTTGGATCACTTTGTTGTAAGTCATTTTTAACATGTATGCCGTAAGCCACTGATATGCGTGCATTAGATTTTTAGGTTCAATCAACTCCTCAACAGTATTCCACGAGCACTTCTCCGTCAACAATTCATCAGCATTGGCCAGGAACTGTACGGAACCATATGGAGTGTCGGCCGATCTTGGTTCTCGTCTCCTGGGCCACAGGTTCAGCAGCCAGTTGCTAGTCTGCTGCAGCAGGAGGCTGTTCTCGCCTTCGTATGTGCAGTTGGCGTCGTTGTCGTTACGTAGATCGCCTATCGACGCCGCTTTCAAATAGCCATGTCCCCCGCAAGCTTCCCTACAGTTCTGAATACCGTCCCGCGCTGTCCAACCGCACAGCGGTTTGGCGGCGGATGATAACGCGTGCATCTCCATACCGCGCGACGGCGCTTCATGGTCCGCAACACCGGTCAAACTGTTAATAGTCATAACGACATGCGCGTAGCCAAACCAGTTGCAAAATATCCTCATGGCGTACGTGGCAGCCAGGTATGGAAGCAGTCGGATTTGCTGCTGCTGGTATTCCAACACAGGTGTTTCCTCATCAGAACTTTCTGGACCAAACTGTCTCCGTACGGCTGAATATCTCACAGCTATAACGATTGCTTTCTGAAGGTAATTCGTCGATATCGATGTTATGAACACTCGACCGCCTGATAAAATGCCCAAGGAAGCACCGAATCTCTTGTTGGGATCCCGGAATGGGGTCTTGTAGTCCCCGTTCTCGTCGACTCCCCCTAATTTGTCCAAAGCCGCTTCCTTTGGCAAGTGGTAGTTGTTGAACATTACAAATCCGTTATCAATTCCGTTCAATCCAATTTTCTCCCCAAGATCACCCACGATGATACCAGGATATGGTTTTAATGTCCCAGGATCTCTTATTGGCACCACGAACGCATGCAATCCGTGATCCTTACCCTTCGATATCAGCATTGCATACACTATGGCGTGAGTTGCGCATTTGCCTAAAGCACCAACCCAGCACTTAGCTGCCTCAAAGTCAGCGGAATGCAACACAAACTGTCTTTTCTCTGGAACGTACGTCGCCGTGGTTCTCATGCCTTTAGCATTTGATCCATGGGCAATTTCTGTCAACGCAAAACAACCTCCGATCTTGGCTTTTTCGATGTCCTCAACGTAGTGATAATGGTGTTCCCGGCCACTGCCGCGTATCACGTTTGCAAACATACGGAATGTGAGTGACACTTTGATAGCCAGGGAACTGTCAAACATGAACATGGCTTCGTTTTCCGCTTGGAATAATCTTGGATCTTCGACTATTTTTTCAATAGGTATAAAATTTTCATTGTATATTGCATACATCCTCTTGATGGTGATGTGACGTTCTTCATCCAGGGTACACGTCGCATTTGGGTTTTGGAAGAGTGGATGGCTTTGCATGAACTTATATACATCATGTTTTAATTTCAACGTGTTCAAATTTTCATAAACTAACTTCATCCGGCGCCAATCAAAAGTGGCTTTTTGTCTATATTTCTCCAATGGACCGGAAGGTAAGTCCGGGAATAAATCCTTTAATTGTTCGTCTGTAACGTGCACTTTATTATCTAATTCCATTATTTGCAATAATTGTTTAAAATCAAATATATATATATATATTACACTTTTACTTTGATAAGAGACTAAATTCTTGAATTAAACTATTTCAACAGGTATAATTAAAAATTGAAGCGCAACTAAGATTCTAGCTGCTATAGTGCCCGAGATTCCTTCACAAACA

>SinsACO5

TCACCATCGCGGTCAGATACTCCGCTGTCAGGAGACAGTGCCAGCCTAAGCCTGGTGAACGTGAGCCTCAGATCCTAGACTACGCGACGCAGCAGCACAAGTTGTTTATATGCATAGCGACGGCGCACGCGTTGCAGCTGGCGGGTGACTGGCTGTGGCGCGCTTTCACCGCGGTGCTCGCGGACATGCGCACGGGCAACACAGACAGCTTGCCTGAGCTTCACGCGTTATCGAGCTGTCTGAAGGCTATAAGCACTTCCGACGTGGCCTCTCTGATAGAGCAGTGTCGGCTCGCGTGCGGGGGTCATGGGTACATGTTGTCCTCGAACCTCCCGCTGCTGTACTCGTTCGTGAGCGCCACCAGGACGTACGAGG

>SinsACO6

GTGTTTGTTATGCAAGATAAGTGGGTATAGTGCTATCTCATTCTGTTCGTCATGTAGTAAGTGACTTGTTATCAATGTTATGTTTATTATCTTTTATAACAAATAAACTATTCGCTATTACATTATTCGCAGCTCGTGTGAGAACTGTGAGTTTATAGTATAGTAATTAGAGTGTTTTTTAAATTAATAAATAACATAAGAAATAATGGTAACTAACAATATTAACGAAGATTTAGCGAGAGAGAGAAGAAAATGTGATTTTAACATTGAAGAGTTGACTAATTATTTGGATGGAGGGGTTATGTATACGGAGAAAAGAAGGAATATTGAGAACAAAGTATTAAGCGTGCAAGGGCTTATGGACAAGGTGCCCGAGGAATATTTGAGTCACAAGGAAAAATATGAAAATTCAATACGCAAGGCCGTATTGCTGTACAGAACTTATCAGCAGATTGACGAAATGAGTCAAACGGGTATTGAAGGAGAAAGGATCCGTTTCCGTAATAGTGTTTCAACAGCAGTATTTAAAGACAATTCCCCGTTCGCTCTGCACATAAGTATGTTTATCCCTGCACTCATGGGTCAGAGTAATGAGGAGCAGAAGAAGTATTGGTTGAAGAGAGCAAATAATATGGAAATAATAGGCACTTACGCTCAGACGGAGCTCGGTCATGGTACATTCATCAGAGGTTTGGAAACAACTGCTACTTATGACCCCGATGCTGAAGAATTTATCCTTCACAGCCCGACTATATCTGCTTACAAATGGTGGCCGGGTGGATTGGCACAAACGGCTAATTACTGCGTGGTCATGGCCCAATTATACATCAGGGGGAAAAACTATGGCATCCAGCCTTTTATAGTGCAAATCCGCGATGAGGAGACACACATGCCTCTGTCGGGGATAAAGTTGGGAGAAATAGGCGCTAAGCTGGGATTCAACACAGTCAACAATGGTTTCCTGGGCTTTGATAATTACAGGATACCAAGAGATAGGATGCTGATGAAGAACGCTCAGGTTTTGAAAGACGGCACGTTCAAAGCATCGCCAAACAGTAAGCTGACGTATGGAACCATGGTGTACGTGAGAGTTATAATAGTCAACGGCATGGCCCATCATTTGGCTAAGGCCGCAACTATCGCGGTCAGATATTCAGCTGTTAGAAGACAATCGCAACCAAAGCCCGGGGTACCTGAACCCCAAATCCTGGACTATGTCACTCAACAACATAAACTTTTCATTGTGATAGCATCGAGTCACGCCTTCAGCATTGTCGCCTGGTGGCTCTCGGAACTATATTTCAGCGTCACAAGAGATTTAGCTGTAGGAAAATTGAAACGTTTACCTGAGTTGCACGCGTTGGCTTGCTGTCTCAAAGCAGTGACCACAAAAGATGCTGCGAGCCTCGTAGAGCAATCCCGGCTCGCGTGTGGTGGGCATGGTTACATGTTGGCCTCCAACCTACCCCAGCTGTACGGGTTAGCCACTGCGGCCTGCACGTATGAAGGGGAAAATACCGTACTGATGTTACAGACTGCCAGGTCTTTAGTGAAGGCTTGGCATCAGGCAACCAAAGGAGAAACTCTGAGTCCTACGATGGCTTATTTATCGGACAAGAATCCTTTGGAACGATGGGATGGTTCCGTCGATGGAATTATAAAAGGGTTCCAGAAAGTGGCTGCTGGTAAAGTGTCATCATGTGTAACCAGTATTAACAAGTACACAAAAGCTGGATTGTCTCCAGAAGACGCCTGGAACAAGACCTCCGTCCAACTCGTCGCTGCTTCAGAGGCCCACTGTCGCGCCGTCATACTGTCTACATACAAGTCCGAAGTGGAAAAGAAGACATCGTCATCCTCCGAAACCCTGAAAATGGTCCTTCATCAATTGGTCCAGTTATACGCTTCATATTGGGCTTTGGAGAGATTGGGTGATTTACTTTTGTACACGTCCTTGACGGAGAGCGACGTGCAAGAGTTGCAGAGCAGTTACGAGGAACTCCTCGCCAAGTTACGGCCCAACGCTGTCGGTCTCGTCGATGCCTTTGATTTTAGAGACGAGATTTTGAATTCAGCGCTAGGCGCGTACGACGGGCGCGTGTAC

>SinsACO7

CGCGCCCGTCGTACGCGCCTAGCGTCGACTGCAGGATTTCATCTCTAACATCGAAAGCGTCCACCAGTCCAACAGCGTTGGGTCGAATTAAGCCTAGAAGTTGCTCATACCACTGCTGTAAACTGTTTATATCTTGCTTTGATATTGATGTGTACCGCAGTAGATCACAAAGTTTCTCCAATGCCCAATAAGTCGCATATAGCTCAACTAACTGATGTAAAACTGGTCGCAATTTAGGAGACGATTTGGAAGTCATCTCTCTAATTTCTTCAAAAAATGTGGATAGAATAATTACTCGACAATGAGCCTCTGCCGCAGCAACCAATTGTACTGAAGTCAAATTCCAAGCTTCCTCATTGGACATTCCAGAGACGAGCCTCTTTTTCATATTGTCAACGCAGGATGATATTTTTCTTGTAGCGATTACTTGAAATCCCCGTATGATTCCTTCAACTGAGTTGTCCCAATTATTTGTGAATCCTTCAACAGAAACGGTCTTCAAATAAGCAACGGTCGGAGGTAATTCTGAAATTTTGACTTGTTGCCATATTTTCACCAGATACCTGGCAGTCTGCAACAGCAAAACAGTATTTTCGCCTTCATAAGTGCAGGCTGCTGTCACTAAACCATATGTGAGCGGAAGGCAGGAAGAGTGCATGTAACCATATCCACCGCAGGACAATCGACAGCTCTCCACGTGACCAGCCGTGTCAGACGTACTGACTGCTTTTAGGCAGCAAGCTAAAGCGTGAAGCTCTGGTAATCTTTCCAGATTTCCTCCAATCAACTGTTCGTTTACATCATTAAAAGTATCCCAAAGTTTTTTAGCCGTTATCCTCAGTGCATGACATGTTGCAATACCTATGATTATTTTATGCTGTTGTGTCACATAGTCCAGGATCTGAATTTCTGGCTCTCCATCTTTGAGTTGTGACTGGCGCCGCACAGCAGAGTACCTGGTAGCTACTATAACAGCTTTAGTTAGATGGTTGACCATGTCGAACACTATGACTACCCTCACAAATACCATTGCTCCGTAGTTTAATTTGCTATTTCGAGATTTCACGTATGTCCCATCCTCTAAAACCTGGGCGTGTTTCATTAACATATGTGTCCTAGGGATTCTGACGTTATCAAAGCCCAAAAACCCGTTATTAACAGGATTAAGACCAAGTTTGGCTCCAATATCTCCCACTTTAATTCCAGGGAGCGGCATATGCGTTTCCTCATCCCTGACCTGGACTATGAACGGATGAATACCGTGACATTGTCCCTTGGTGTACAGTTGAGCAACTACAATACAGTAATTCACAGTGTGTCCCAGTCCGCCGGGCCACCACTTGTAGGCGGTGAGCGTGGGGCTGTGCAGCACGAACTCGCGCGACGCCGGCTCGTACGTCGCCGTCGTCTCCAGCCCGCGGATGAACGTGCCGTGGCCCAGCTCCGTTTGTGCATAACTGCCAATAATGGTAGAATTCCATGCTCTGGCAAGCCAGTGCGCCTGCTGCTCCTCATCGCCCTGATTCAGGATCGCAGGCATGAACATGATGTAGTGCAGACCGAGCGGAGAACCGTCGCGGAAGCTGGAGGAGCCGAGAAGACCGCCCACTACATCCCTATAATTAGTAATGCTGGCCTTTCCTTCCTCTTGCATGCGGCGCACCAATTTCATTACCGCAACTGACTTTCGAACTGCATTCTCATATTTCTCTTTGTGGCTAAGATATTCCTCTGGAATGTCATCAAATATTTCTTTTTGACTTAAAAACACGTCTTCTCGTTGTTTCCTTTCTATAGTCCTCTGCTCTCCTCCATCTATCAAAGTTGTTAGTTCGTTTATATCGAACGAACACTTTCCCCTTTCTCTTTGCAAATCGGGATTTATTTCACACATGTTATCTTTCAACTGAATACTACTG

>SinsACO8

TAAAGAAGTATCTCCATGTCTGCTACATCTAGGCATGTTTGTGCCCGCGATCATGGGGCAATGTACGCCCGAACAGCAAGCGTATTGGCTGCCACGAGCGCTGGACATGCAAATTGTGGGAACTTATGCCCAGACAGAGTTAGGTCATGGCACGTTCATTCGCGGACTGGAGACAATCGCAACATACGACCCAAACACCGAGGAGTTCGTATTGCATAGCCCCACGCTAAGTTCGTATAAATGGTGGGCCGGAGGGCTTGGCAACACAGTGAACTATTG

**ACD:**

>SinsACD1

AAGGCTTCATATAGCCAGAGCCGAAAAAACTAATTTAAAAAAACGTGCTTATAATGGTTATATTCGTATTTCGTATAAATGTATAATGATAATGTACTTTGTGTTCACTATATCTAAATTTTTTATATTTTGTGTCGCTTATCTACAATAATTTCCTGTTATCATTTATTACGGTCAACTATCTGTTCATGCCTGTTACATTAATTGTTTATTTAAGTTATTATTTTTCATAGCTGTGTGTGTATAGCATAGATTAGAGTTTTAAAAACTTTAATTACTTGGGTGCCTATCTGTTATCGCAATATTCTATATAACATAATTTTTTCAAAATGTTTAAAGGTCTAGTAGGTTTAGTAACTGGGGGAGCGTCAGGGCTTGGCCGTGCAACAGCAGAGCAGCTCCTTAAACAAGGTGGTCGCGTTGTAATATGTGACTTGCCTTCTAGTCCTGGTAAAGATGTAGCCAAGCAATTTGGTGAGAATGCTGCATTTGCACCTCTCGATGTGACATCCGAAGAAGATGTAAACAATGCATTACAGTTAACTCGTGACAAGTTCGGTCGGCTCGATGTACTAGTCAATTGTGCAGGAATTGCCACTGCAGTTAGAACTTATAATTTCAAAAAGAATCAACCGTTTGATCTGAAACAGTTCCAGAGAACTATTGAGGTCAATCTAATAGGCACATTTAACGTGATCCGGTTAGCAGCTGGTATGATTGGAAAGAACGCGCCAGATGCAGACGGTCAAAGAGGCGTTATAATCAATACAGCAAGTGTGGCTGCTTTCGATGGACAAATCGGTCAAGCGGCATACTCTGCATCAAAAGCCGGCGTTGTTGGTATGACGTTACCCATTGCGAGAGACTTTTCTAAAGCAGGCATCAGAGTCGTTACTATAGCGCCTGGTCTGTTCCGCACGCCCATGATGGAACAACTCCCCGAACCGGCCATTGCGCAACTGGAAGCGTCAATACCATTTCCATCGCGGCTCGGTCATCCTAGTGAATTCGCTCTGCTCGTTCAAAGTATCATACAGAACCCGATGCTCAATGGTGAAACTATACGTCTAGACGGCTCGTTAAGAATGCAACCTTAGTTTCTTAAATAATAGTCGTGGATGAAATAGAATGGGTTCAGTACCTCTTTGTTTGAAATACAATGAATGTAAGGTATATAATTGTAATGTAGAAATATATTGGGTTTTATAAAAAAA

>SinsACD2

CACGGATAAAAAGGCGTTAACATCAGTAGACAAAAAACTACTAGATTACTTGTATTGCATAAATACTAGCAACATTTCAAAATGTTGCCAGACGTCTGCCCAGTTATATTTTTTTATTCGGGTAATAATAGGTACTCTATCTAGTCTCTGATGTGTTGCCCCCATAGGATTGTCCATATTACGATGTATAGAGTTGACGGGGACTGCACTTGGTAGTCGCGCCAAGTACTCTATGATCCGCCCGATACTTTTGTTTAATTAGTTATAGTTCGTTTCTTTTTTGTACATCGTGTAGAGAAATATATTGTACGCAAATTAAGTAGATAAAATTGTTTAGTTTACGTGTCCATTAACTAGTTGTTTCGTACATAATTCATAATTATCGTGAATAGAACCCAAATACAATTACTAAATTAAATGTTTGTAAATTATTAAACTTTTTTGTTTCGGAAATTTAGTTCCACGTTTTATTATCTGATCATGAAGAGTGTAAAGCTGGTGACTGGCGCGTCGCGCTGCCTGGGAGCTGTTCCTGGCACCTCTGGAAAGCCCTCGCTGTACAGTGCATGTGGTGGATGCGGGAGCGTACGGGGGCTAGCGGACAAGGGCGCGCCGCGAGCGGGTGCGCGCCAGAGCGGCTCCTTTACGCTGAACCTGTTCCGCGGCCAGCTGGAGACGGCACAGGTGTTTCCGTATCCCGAGGTGCTTTCCGATGACCAGAGACAGATGCTCACCGAACTCGTGCCGCCTGTTGAGAAGTTCTTCCAGGAGGTGAACGATCCCGCGCGGAACGATGAGACGGCGCAGATCGAGGAGGGCACGCTGAAGGGGCTGTGGGACCTGGGCTTGTTCGGGCTGCAGGTGCCGGCTGAGCTGGGAGGCCTGGGCCTCTCCAACACGCAGTACGCGCGCCTCGTCGAGGTGGTGGGCGCGCACGACCTGGGCGTGGGCATCACGCTGGGCGCGCACCAGTCCATCGGGTTCAAGGGCATCCTGCTGTTCGGCACGCCGGAGCAGAAGAGCCGCTACCTGCCGCGCGTCACGGGCGGCGAGTACGCCGCCTTCTGCCTCACCGAGCCGTCCTCCGGCTCCGACGCGGGCTCTATCAAGACGCGCGCAGTGCTCGCGCCCGACGGCAAGCACTTCGTACTGAACGGGTCCAAGATATGGATCAGCAACGGCGGCATCGCCGAGATCATGACCGTGTTCGCGCAGACGCCCATCGAGAAGGACGGCAAAACCGTCGATAAGGTGACGGCGTTCATAGTGGAGCGGTCGTTCGGCGGCGTGTCGTCGGGCCCGCCGGAGAACAAGATGGGCATCAAGTGCTCGAACACGGCGGAGGTGTACTACGAGGACGTGCGCGTGCCGGTGGAGAACGTGCTCGGCGGGCTCGGCAACGGGTTTAAAGTGGCGATGAACATCCTCAACAACGGCCGCTTCGGTATGGCGGCGGCGCTGGCGGGCACGCAGCGCTCGGCGCTGCGCGTGGCGACCGAGCACGCCGCCACGCGCACGCAGTTCGGCAAGCGGCTGGCCGAGTTCGGCGCCGTGCAGGAGAAGCTAGCGCGCATGGCCATGCTGCAGTACGTCACCGAGGCGCTCGCCTACATGGTCAGCGGGAACATGGACGCCGGCTGCCAGGACTACCACCTCGAAGCCGCCATCTCCAAGGTGTTCGCTTCGGACTCAGCGTGGACGGTGGTGGACGAGGCCATCCAAATCCTTGGCGGCATGGGCTACATGAAGGCGACGGGCCTTGAGCGCGTGTTGCGCGACCTGCGCATCTTCCGTATCTTCGAGGGCACCAACGACATCCTTCGCCTCTTCGTCGCACTCACTGGCATCCAGTTCGCGGGCTCGCACCTTCAGGAGTTGCAGCGCGCGTTCAAGAACCCGACGGCGCACCTCGGGCTGATCTTCAGCGAGGCGGGGCGTCGCGCCGGGCGCGCCGTGGGGCTCGCGCGACTGCCCGACATCGGGCCGCTGGTGGCGCCGCCGCTGCAGCCGGTGGCGCGCGAGCTCGCCAAATGCGTGGACGCATTCGGCGCCGCGGTGGAGGCCACGCTCGTCAAGTACGGACGCGGCGTCGTCGACCGCCAATTCGTGCTCAACCGACTAGCGGCGGCCGCGATCGACGCGTACACGACGGCGGCGGCGGTGTCGCGCGCATCCCGCGCCGCGCGCCTCTCGCTGCCGACAGCCGCCGACGAGCTGCGACTGGCGGAGGCGTGGGCGCAGGACGCGGTGGCACGCATCCCCGTGCTGTTGGCGGCGGCGCGCGAGCAAGACGCGCTGGCGCAGTATTCGCGCCTCGCAGACATCGGCACCGCCGTCGCCGGCCGCGGCGGACAGCTCACCGGCAACCCGATCAACCTGTAGCGGCCACGCAACTGACGCACCCCGCGCGCGCCTCTCATAGACTGTAGTGTCGTCTCGTCTAGCGTGTGTCGCACATTTTCGTTCGGAACGTTATTATGATGGACTCCCGCGTTTTCATCCCGTTGCGCGAACCGTTTCGTCCCAGTCCAAAGGCGCCGACCGGTCCGGTGACATCGAGCTGCCCGGCCGGAGGAATGAAAACTAAACTTCTTTTCATATTCACTATTTAATCAGGCACAAAATATGTACAAATATTGAACCGTAACGTCGGTCGACTACGCGTCGATCTGCAGTGAGCAGTCCCAGGCCTTGGCGTTGACCTGCTTGAGAGTGGCGCGCAGCGCGGCGCCGGGTCCGAGCGCGAGCGTGAGCGGGAAGGCGTCGCCGGGCGTGCGCGCGTATATCGCGTGCAGTGTCTGCTCCCAGCGCACGGGGCACATGACCTGCTTGGGTAGCTGCGTGCGCACGTGCGAGGCGGACGCGTAGGGCCGCCCGTCTACGGACGAGTAGACCTTGAGCGCGGGCGCGCGCACGTCGCACAGCGCCACAGCGGAACGCACGGCGTCGGCAGCGGCGCGCATGGCCGCCGTGTGGAACGCTCCCGCTACACGCACGCGCGCCGCGCGCCGCACGCCGTACTGCTTGCCCTCCCGCTCCAAGTACTTCAAGGCCTGAAACGACACGGAAGTCATGTTGAAAGTTTCGAGCGAATTCATTTGGTCGGACTATTATCGACTTTGTTTATATTGTTAATGTGTAAATTAAAATAATAAATATATAGATCAATTAATTTATTATAAAAATGATATTTCATTCAAAATCTGATCTGGCTCTTTTGTCTCTCTGAGAAATTATTTTACTCTAATTTCCTCGAACTCTAAGTTGCCCAAACACAGAGGCTGCTTAAACGGTGCTCCGGGCGAGCAATCCGATATACGTCTGGTTCCAGTTTCATCACAGATGATCAAACAGAAATTTGAATAGGAATATTTAAATTGTGATAAAAATACTTGTACACGATTCACAATAAATAAATAAATAAAGTATATATATTTAAGATGGGACAAGAAAAGTGATGCTTAGGATGAAGCAGAGACAGTGCGCCACCGGCGCAGGCATTTATTAGCCTCTTGATCTGAAGAGACGCCGAGTTGTGAGAATCAGGAAGTAATAGTTTATTCCACAATTTGTTAGTATGTGGGACGAGTCGGCAAATTGCTTAGATCGAATAAAGGTGTATTAACCACACAATTTATTCTTGCGTGCATAAGCTTCGACGCGTGCTGACCCTAACTGGGCACTAATGAGCCGATGATGAAAAGGAACTGAAGGAATTAAACTGCGCACGCTCTCCAAAATGTATATTTATATTCAAAATAATACATTTTGAATATAAAAATATAATGCAATATTTTACTACTTTAAGTGAGCAAATCTTATATAACTTCCAGAAAAAAATCACTCGTAGACCTGATGGTTTAGCTAACACATGTTTTGTTTCAAA

>SinsACD3

GACAGTTTTCATTTTACTATAAAATTACGCATGTATTGGTATTGCAACAATTTCTCAAAATTTCAAGTCAATAGATCGACTACTTATCGAGTTTTGCAACCACAAACGTACCGAGAGAGGCCGAATTTTTATATTATATACATGTAAGATTACTTAACTATTTATATACATATTATACTAATATGTATTAACATTTAAAGAGGAAACCATCTTTGAGCACCATCTAATCTTATAACTTCACCGTTTAGCATAGGATTCTCAATTACACTTGTAACTAAATGCGCAAATTCTTCGGGCTTGCCTAATCTTGAAGGAAATGGTATCATACGTTTAAGAAAATCTAACACTTTCTCCGGTAAATCCGACAATAATAATGGAGTTTCAAATAGTCCTGGCGCAATAGTGACTACTCTAATGCCCTGCGGTGCTAAATCCCTCGCTATGGGTAAAGTCATACCAACAATGGCGGCTGTTGAAGCCGCGTAAGCTGCCTGTCCTATGTCTGCTTCAAATGCGAATGTCGAAGCTGTGTTGACAATAACACCTCTCTGTCCATCGACGTTAGGTTCATTTTTGCCTATCACACCTGCAGCTAATCGTATTGCGTTAAATGTACCTATTGTGTTAACATTTACACATTTGATAAAATGATCCAAATTACATGGTTTGTCTTTATTAAAATTGTACGTCTGGTATGCCTGAGCAGATCCTGCACAATTGACGAGTGTGTCAAGGCGACCAAACTTATCACTAGCTAAGTTTATTGCTTTATTTACGTCTTCTTCAGATGTGATACATCCGGAACTAGCAATCACATTTTCACCAAGTCGTTTCGCCACTTCCTCCGCCCTTGTACCCTGAATATCTAAGATCACAACCTTTCCACCGCTCTTCACTAACCTCTCAACGGTCGCTTTGCCAAGACCTGATGCACCACCAGTCACCAAACTAACCATACCCTTTAACATTGTTGTATTTCTTAGAAATTCTGTGTGAAAATTATATTTTTTATATAGTAACTAAAGTAAGCGGTAAATTTGTTTTTACAGATTATAA

>SinsACD4

TTTTTTTTTAATTTAGAAAATATAGTTTATTTATTGCACATCTTAAAAACATACATAATTAGTGTGTAAAAAGTCACAATATAATACGCTTAAAAACTTCAAAACATAACTTCATGAAGTTGTAAAACTTTCCAAGTAAATCACAGTCTTATTTCTTGTCATATTTGTAGAAACCTTCGCCTGTCTTCACGCCGAGTTTGCCTTCGGACACCAATTTATTCAACAGCGGTATTGGGTTGAACAGCGGTTCTTGGGGATATTTCTTGTGCCAACCGTCCAAGATGAACTTGTTGGTGTCCAAACCCACATAATCGGCGAGCTCTAAGGGTCCCATGGGATAGCCTGCTCCTAATTTCATAGCGATGTCGATATCGCGAGCTGACGCGTCACCTCTCTCAAACAATCTAATAGCCTCTGCTATATATGGTACCAGCAGTCTGTTGACCACGAAGCCGGGTGTATCCTTGCATGTAATGCAGGTCTTGCCGACGGCCTTGCCCCATTCCATCATGGCTTTGTAGGTCGCTTCCGATGTCTGATCGCCGCGAACCACTTCGAGGAGACGCATCACTGGCACGGGGTTGAAAAAGTGCAGGCCACCAAATTTATCTTTTCTGTTGACAACTGAAGAGATCTCATTGATTGATAGAGATGATGTGTTGGACGCGAAAATAGTGTGGTTCGGAGCAACCCCATCCAGTTGCTTGAAAAGTTTATGTTTCACTTCAATGTTCTCAACAATGGCTTCCACAACTAAATCTGCATTTTTAGACGCGACCCCAGGGTCAGTAGAAGTTTTGATTCTACCAAGAGACTCGGTAACAAATTTCTCGCCTTCCTGAGGATTATCCTTGTATAATTTCTTAGCTACACGACCAAGGTTCGTGCCAATTGATTTCTGCGCTTTCGCTAGTACATCTGAACTGACGTCTACAAGAGTCACATTTTGACCAGCTTGAGCAGACACCTGGGCGATACCAGAACCCATAAGGCCTCCTCCGATTATAGTTACATTCTTAATTGCACTTTGAACAGCAGCAGAACTAGAAAAGTTTCTAGCGATAACTCCAAATTGAATCATTTTTATTGCACTTTTGCTTACCTACATAAAATTCTTAATTAAAACTTGTTTTTTTTTTTTAATTTACAATAAAATAATTGATATATTATGTGTGTAAGACGTTTTATTCTATAAAAAAAAGTTAAAACATTTAATTCCAAAGTTCAATCTACTGCCCAAAATTAAAGTGGC

>SinsACD5

AGAGCATCGATAAAGATATCTAAAACAATAATACATAATTACTTGTGATGTGTGTATATCACAATCAATTTGGTCGAAACATGTTATCGTACCCGACTGAGATAACATTAAAAACTAGCCTCTAATATTATGTGCTTGGAATCAAACCAGTTTCATTCCGGCCCGGCCTGCGCAAGCGCGACCCGCATATTCAACTAGCGCACGTTGCATTTGCAAATAGAATAAAATAAGTATAAAAAGAAAAAAACAAAACGGCATTATGTTCCCATTGCGTCGTTTAGTTGGCAGTAAGATATTGGAGCAATGGCGGGCGCCTGTAGCAGTCGGATCTAAGATTCAAAATAAAAATTTCAGCTCGGAGGCTGGTACTCCGCGACCTTTGAGCTTGTTGACAGATGATGAAGTGGCCATGAAAGAAACAATCAGAAAATTGGCAACGGAACAAATAGCACCTCTAGTCAGGAAAATGGAAGATGAGCACAAAATTGATGAAGGCATCAGACAGTTGCTGTTCGATAATGGTTTAATGGGAATTGAAACCCCAACGGAATATAGTGGTTCGGGGTGTGGATTTCTCACTATGATGCTGGTGGTAGAAGAACTGTCCAGGGTTGATCCAGCAGTAGGGGCGTACGTGGACATCCATAACACTTTAGTCAATTCCCTCTTCATGAAGCTGGGCACAGAGGAACAGAAGAAAAAATACTTGACCAAACTCTGCACGGAATATGCCGGCAGTTTTTGCTTGACAGAACCGACATCGGGATCTGATGCATTTGCCCTAAAAACTGTTGCCAAGAAAGATGGCAAAGATTACGTCATAAATGGCTCTAAAATGTGGATCTCAAACTCAGACGTCGCTGGAGTTTTCCTCGTTATGGCCAACGCTGATCCATCCAAGGGCTACAAAGGCATCACCTGCTTTATAGTAGAACGGGAGACCCCTGGCCTGACCGTCGCCAAGCCCGAGAACAAGCTCGGTATCCGTGCGTCTGGCACGTGCATGGTGCACTTCGACAACGTCAGAGTTCCCGAGGAAAATATTCTTGGAGAATACGGGCATGGTTACAAATACGCGGCAGGATTCCTGAACGAAGGTCGAATCGGTATCGCGTCTCAAATGATTGGCCTCTGTCAGGGATGTATGGACGCCACCATTCCGTACACACTTGAAAGGAAACAGTTCGGACAAAAGATTTACGCTTTCCAGGGTATCAGCTATCAAATAGCTCATCTCCAAACTCAACTGGAAGCGGCCCGCTTGCTCACGTATAACGCCGCTAGACTGAAGGAGCATGGTATGGATTTCGTAAAAGAGGCAGCCATGGCGAAATATTTCGCTTCAGAAATCGCTCAAACCTTAACATCCAAGTGCATAGACTTCATGGGCGGAGTGGGCTTCACTCGCGACTTCCCGCAGGAGAAATTCTACAGGGACGCCAAAATAGGCACCATTTATGAGGGTACCAGCAACATGCAACTGCAAACAATAGCCAAACTTATCGAAAGAGAGTATTCACAATAAACTGTTTTTAAATCAAAAAAAA

>SinsACD6

CAACTACTGGCAACACTATTAAAATAATAAATGTCATCAGCATCAAAATAAAATAACCTCAAAATCTTATTCCACTAAACCACAAATAAAGAACATATTTTGTGTTAAGATTAACATTAAACATTATTTAATATATAATTTATATATATATAAGACGTCATTCTTTATTCTTTAAAAATGAATATTGCTCGCACTATATCTAAATTTCGATTAACAAATATCACTAAGAATGTTTATAGAGAACTACGTATTTCTTCTATTAATTGTGAATCTACACATGCCTCACAGCCAAAAGTGCAAGAAGAGAAGTTCGATTTCGAAGATCTAAAAGTTCTAGAACGAACCGAACGTAGAAAAGCTCAAATTCAGCCTTTTATGAAGAATATTTTCGTGTCCGTATTCGACCGAGAATTACTTGCTTACCCAGAGATTTTAAATAAAGAAGAAACAGAGGCGTTAGAAAGTAGAGTTTCTGCGTTAGAAACTGTTTTCTCCGACCCGAAAAAGACTTCTGAAGACCGAAGAAATGCTTTAAAAAAGACACAAATGTACGCCGCACCAGTGAAACTCACTAATAATGGCTTAGCAATGAATTTCACTGAAGCCCTAACGTACTTAGAGACTATATCGTCAGATTTGAGGCTTGGTAAGGAAATCAGCGACCATTGGGTGGGTTTACAAGCATTGGAAAAAGGCTTATCACCAGACAACTACCAACAAATTGTAAATGATATTACGTCGGGAGAAAATTCTATTGCATTGTGTATTAAAGAAAAAATATCAGAGAGAATAACACAGCCAGATTTTAGAACTAAAGGCGAATTGAACCAGCATGGTGTATGGCATGTATCGGGCGAGAAAGTCTGCCACCAAGCCAACGGATATTTTCTTGTACTAAGCGTAGTTGAAGGAAACAGAATGAAAGCGTTCCTCATCCATCCCGGAGCAAAGGGGGTCACCTTTGATGGGAACTTCATCACTTTTAGAAACACTCCAGCCACCCCTCTGGACGAGATCAGTGAAGCATCTCTCGCTCAAGTATTGGGAACTTCGCGTCTCCACACCGCGGTGCTGTGTCGCAACAGTGTCAAACGGGCTTTACGAACGTGTCTTGACTTTGTGAAGACCAAAGCATTGTCTGGCAAGCCTCTGATCGATCTTTCGACCATTCGTGCTATGATTGGCGAAGCGTTACTGGATATATACGCTTCAGAGAGTGCCGAATACTTCACCGCCGGTCTCCTTGATGGATACCAAAACCCTGATGCTGAACTGGAAATGGCTATGTGCAGAAACTTCATATCGAACCGTGGCATAAAAACCATAATCAATCTCCTCGCCATACCGGCGATGGAGCACGAGGAGGAGTGCAACAGGATCCTGAACGAAGCCAGACATCTGACGTTGAATGGTGAAACGCTGGACAGCGTCAACATGTTTATAGCACTGAACGGTCTACATCACGCTGGCAAAACGTTGGCAGAGGAAATAAAACAAATAAGGAATCCATTGCTCCATCCAACTTTTATTATAAAGAAAATATTCGAAGACAGACATCAGGTAAAAGACGATCCAAAGCTAACACTATACTTGGCTGAATACCTCCATCCCTCGTTGAGGCAGCCGGCGAATCAATTGGAGTACTGCGTGCTGAGAATGCGATACGCGTGCGAAACCCTCATGAGCAGACACGGAATGGAAGTGGCCAATGCTGTCACTGAACTGAATAGATTGGCCGAAGCGGCATCGGAGATATTGGCCATGACATCGGTACTGGCGAGAGCGTCCAGGTCTTACTGTATCGGTATTCGCAACGCCGAAATGGAAATGAAGCTGGCCGCCTGTTTCGTGGAGAAGACGAGGGACAAAGTCAAGAAACTTATCAAGGACATCGACGATGGGGAGTATCTAAACTTGGACAATTTCGTCATACAGTTCGGCAAGAGAGTGGTCGAAACCAACAGCACGAGTATAGTTGAGAAGCCTACCGCTAGGGTGTTTTGGTAGAAATTTTTCACGAGTCTATCTATACCGCATACACGGGGAGCTTTGCGTTTGTGCGAGCGGGCGCCGAATGTACGAAATAGGGGGTAAATCAGTAGTCGATACAACTTGATACTTTCAGACGAGAATTCGCACAAATGCAAAAAGAAATTGCGCACCGTACACCTGATAGAAGTGAAACTTCTCAATGTAGCTGCTTTTCAACCCTCGTAATAGGCTTTTCCGGTATAAACCATTTTGGTAAATTATAGATTTACCAAATATTACGTTTTGGTAAATTATAGTTTACCAAATATTACGTTTTGGTAAATTATAG

>SinsACD7

AAATTTCAATCTTAAAAATTTTATAAATTACGGTTGAATTTCGACCGTTCACGGGGCCTTTAGTTAATCATTAAGACACAAGCGTATACATAATTACAGTCGCAAGCGTATACGTAATTAAATTTAAACATACCAACCCCGATTGACACGGCCCGACTCGAAACTCAGCACGATGGTTCGCGAACGCGAATAGTTGTGATTCTGTCTTGTACGTTGTATAAATCGAATAAGTGGCCGTTCTACTACATTAATATTTAATACGGTACGGTAGTATTATACTAATTCTATGCCAATGCGTTCTCTGCCATACAGGACTGTTATTAGACCTCTTTCAAACAAAAGTTTACATTCAAAAAAAAAAAAAACTGTAACGACTCTGTGTGTGCTATGCTATGTAAAATGTGTCAAGTCTAAAATGTGTTCAATCAAAAATTGCGTAGATCGTATTATGTACCGCACAGGTTTTCTACAGGATCTTTTTATTTTTAGATGTATTGTGTCATCAAGTCCAACGTTAAAGTTAAGATAAAAATATGAACGAATTTGGCTTGTGGAATGTTGATGATTCGACATCGACCCATATACGAGCCACTGCCACTGCTGGGCCCGGGTCTGCTCTCAACTGTGAGAGGGTACGGGTCATGCTCCACCCGCGGGCCCAGGGCGGGGCGGCGGAGATTTGTGGAAGGAAGGACAGTGACCAACCGTACTCTCAGAATTTTATCAAGCTGCTTATGGCAAAGGTATCTGACAAGGTTTAACAACTTTCATAATGTGTAAACATTATGAAAATCGACACCAACTACTTCACGTGCCTTCTGTAGCACGGAGACGTTCGAGATGTGCCACTGCAGCGGTCACCCATCCAGCTGCCGACCGCGGTATGCGTTGGATAACCACGAAGCCGCAATGTTGATGATTGTTGACTTAACATATTTTATGCAGTAGAACACCAAAGTAATATTTTTAACATTAATGTTTTTAATCTTTTTGTTTAAACAGTTTTTCTCTTATCGAGCATTTTCCATTTAGATCTTAGTGGAATGCACGTCAACTCTTCCTACGCACTTCTTTTACATCATGAATATGAACGTACATATGAAGAAATATTCTTAATGAAAAAAAAAAAACTTAAGAATTAAAGACCGCAAAACGTACTACTTATCTGTACCTCACAAGTAAACTTCCAGAATTTTACAATAGAGATACCTTAGAGATACAGCATTTGCAGGAGTTGAAAAAAGTTATTATTTGCGAAATTATAATCTGTACTGATGTGTTCCTTATGGCAATCAAATAATCAAACCCGAATTTCTTCAATTGTACGTAAGTGTGGTGGTGATTACGACAATATTAAGTAACCTCTTAACAAATTTAACATTAACAAATATATACATATATAATTCTGATGTTCATTTACAATTTATTGTCCTAAAATTTTAATGGCGTAATATTTAGATCGATTGAGCGTTAAAATGGGCAGCTATCGATTTATCAATATTTCAAGAGCATGTAAGGCCGTTAGTAAATATTAACAAGTCATTTCTAGGCCTGCCAATACCAATTACAAATATCGATTACAATTCAACAATACGGCACGACTATTTGTATCCCAGTCTTATACAATGACAGCGCGTTAACTTGAGATTAGACTAGCAAGCGTTACCGGTTTTACACGTCAGAGGGCGTCGTGTTCTTTGAGAAGAAAGTGTCCGACGAGACGCTTCTGTATGTCCGTCACACCACCGTATATTTGTGTGCCTCTTGCGTCCCGGAAGTGCCTCTCGGCGTCATAGTCGGTGGAGAGCCCTCTGCCGCCGAGGATCTGCACGCAGTGATCGGCGACGGCGGTCGCGTTGCGCCCCGACACGTACTTTGCCATGGAGCTGTTCTTGGTGCACACCTCGACCGCCGCGCGGTACGTCAGCAGGCGCGCCGACTCCACCAATATGCACATGTCTGTTAGACGGTCCTTGACTGACGGTAATCTGGTGAGACTCTTGCCAAAAGCCTCTCTCTTCTTCGCGTAGTTGATCGCCGTGTCCAGAGCCGACTGCGCGATGCCAACAGCATGGGCTGCAATGCCAATCCTGCCGCGGTCTAGCTGCTCCATGGCAATCTTGAACCCCTCGCCCGGTTCGCCCACCATGTAACTGCTTGGCACTCGAACTTCGGAGAGCGTGATGTCGCACGCCGTGGCCGCCCTGTGACAGACGTTGACACCAATTAGTGGTTCTTTTTTCCCTCTGAACACGCCTTCTGCGTCCAAAGGCACCAAGAAACAGACAAGCCCCTTATGTCTGAGTTCAGGATCGAAGGTGGCGAAGACGGCGGTGGCGTTCCCTTCGATGGCCGACGTGACCCAGCTCTTTTTGCCATTCAGTATCCAATAGTCACCGTCACGTGTCGCTGTAGTCTTTATACTGGCTACGTCGCTGCCCGCTCCTGGTTCACTAAGAGCGAAACATCCAACAGCGCCATTGGTGAAATCCTTCAAGAATATCTCCTTCTGCTCCGGAGTGCCTTTCTCATCGACCAGATTGGCGTACAGTAGATTGTGAATGGACATCACCATGCCCGTGCTGGCGCAACCGCGAGATATCTCCTCGACCGCTACAGCGACCGATAGATAGTCCAAGCCTAATCCACCGTACGTTGCGTCCACGCATGCACCCATTAGACCTAGTTCTGCTAACTTCTTTATCTGACTGAAAGGGAATCGCCCTTGTAAGTCCAGCTTTGAAGCATTAGGTTTCAGATGCTCATTAGTAAAATTCCTGGCCAACTCCTGGATGGCCAGTTGCTGTTCATTTAAGTGGCTGGTGAAAGTCCGGCTTTGAGATGATAATATCTTTGCACATTGCAGAGAATACAGAGTAAACTTTGTAGATTTCAGAAGTGCGCTCGCTGCCATGATTGTAGATAACAAATGGAAGTAAAAAATATCACACAATGAGTTGGATCATACACAGATATGACACAGTTGACAATAAAATACGATAATGTATGATAATAACTCAAGGTCAATGGTATAAGGTTAAAAATTCAGTTGTTTTTAATCCAAACAATCGGTACCTACAGTAGGCGCCAATTTTATAATTGGAAAAAGACCTTAACATGAGTAGAATTCCGGTCGTAAAACGCTTTAGAGTAACACTGATTCGTAAATGTGATTAATAAAATAATTAGTTAAGCTATCAGCCGACGCAGTTATAGATGCAAATTAATTTTAATTTCAAAACGATTATTAAAAACGTATATACAAAACACAATACAATGTCCCAGAAAATAATTTGTTGCCACTTG

>SinsACD8

GCATATAAACAAACATACAAAATCAGCTCTCTCTCTCTTTACATTACTATAGTTTTAATATTATTCTTACTTATGATTTGTCGACAAATTGCTATTCGGTAAAACCATAAAAGAAAATTTATTCTATACCGTCGTCTTCAATTTTGTAGCCATAGGGAAAGATCATTTGAAAAGACACTGGAGCATTTAAAATACTATTACCTAGTTGTAAATATTTTTTGTGTAATCCCAAGTTGAATCGAATCGATTCGGAATGGTTTAGTGATTAGGGTAGTTATATTTGTAAAAAAAATACATATATATACATAATAAAAATAAATACATATATATAAATAAAAAAAAATAGATATATATACAATAATAATGTTTATTTGTGTCAGTTAACCATTCACAATAATACGCAATAATAATTTCGTTTACACCATTTTCATACCGTACACACGAATTTTTATTTTATAAAAAATCTATATTAACACCATTTCAACACAACACATTTGTTGATATATGAACTTTTATAACTTGATTAGACTACATAAAAATACAAGTTATAAATAATTTATTCTTTTTTATTGTGATTGTGAGAATTAAATATACATTTTAAATTAAATGTTTAAGATACAAACAAATTAATTTAGGACTTCTGTGTATATTTAAGAATTTAAAAAACGATGTTTATTCATTAGACTGTTTGGCTTCGGTGAATAGTTCCCTCGAAATAATGAGCCTTTGGATCTGTGATGTGCCCTCGTAAATCTGATAGATCTTAGCGTCTCTCATAAGTTTTTCGACTGGATATTCGGTGTTGAAACCGTTTCCACCGAATATTTGGACAGCATCGGTCGCAGCTTTATTGGCAACGTCGGCAGCGTGACACTTAGCTACTGACGCTATCACGGTGTTCTTTTGACCGTGATCGGCCATCCACGCAGATTTCTGCCAAGCCATACGAGCAGTTTCAACGCCGATCGCCATGTCGGAAAGCATAAAAGCAACCGCTTGATGATAAGCTATTGGCACGCCGAAGGTTTTACGTTCCAGAGAATATTTTGTAGCTTCGTATAAAGCCCTTCTCGCTAGACCCGTAGCACCGGCAGCAACCGGTGGTCGGGTTATGTCGAAGGTGCCCATAGCAATCTTGAAACCAGCGCCTTCCCCTTGAAGCACATTTTCCTTAGGAATGCGCACATCCTCGAACGTGATGCCTCGCGTATCCGAAGCGCGCTGTCCCATGTTTTGTTCCTTACGTCCTACAGTGACACCAGGCCATTCCCTTTCGACAATAAATCCAGTGAAAGCTTTGCTTGCTGGACATGTGGGATCTGGGTTTGTTCGGGCCAATACGAAGTACCAATTAGCAACACCACCGTTGGTGATCCACATTTTCTGACCGTTTAGAATCCATTCGTCTCCCTTTTTCTCTGCTTTAGTTTTGACACCAGCCACGTCGGAACCACAGCCAGGTTCAGTAACGCAATATGCTGCTACAAGCGGCTCTTCAATCAGTCTACCGAGATATTTTTTTTGTTGCTCCTTAGTACCAGCGAGAAGAACTGGGGCTTGTCCTAAATTCGAACTTGTCAAAACTAAACTTATACCAGCGCATCCGAAAGCTGTTTCTTCAATAATAATACATTCGTCGTATATTCCCATACTTCCCACTCCACCGCAATATTCAGGGATGTGTCCATTCATCAATCCGAGTTCCCAGGCTTTCTTGATGATGGGCCATGGGTACTCCCCAGTTTTGTCGTATTGGGCTGCTACTGGAATGATTTCCTCTTTGGTGAACTTGCGAGCCAAATCTTGAAGTGCCTTTTGCTCGTCGTTTAGTTCATAGCAAACGCCAGTGGCAGGGATTGGTTTCTTGATAAAACTTAAGGCTGGTGTAGTAGAGAGTTTCCTATATATGGGGCGAGTAGCACGAGCAATCTGGGATATAGGATTCATTATTTCCGAATATGGAATATTTCTAAATTAAACTAAATAATTTATATTGATATTTGGTAATATTAAAATTGTATGGACTTTGTACAGCGTTGGTCGCAATAACAAGAATAAAAAAAAAGTTATATTCGTATAAACGTCAAAATCGCAAATAGAGTACAAAAAATAATGACGGCTGACGAACCACTGACGAACTCTAGTTAGAGCTCCACATTTTTATATTGGCGTAGAATAATAAATTGTACGTGTACTTTGACCACTTATAGCTTAACAATAATTTTAATGATTCGTTAAATATTAATTACAGAAAAGTAGTACGATTCTTTAAATATATATAAATTGGTTAGATTTTTTAATTTTGCATTTTAACTACTTTGGCT

>SinsACD9

AGGCTCCATATAGCCAGAACCGTAAAAGCTTAAAGAAATAACAAAAAATAAGTTTTTCAGAGTAGGTACTAGAAAACTGATTGCCTATATATCTTTGCAGAGTACTTTTATTATTTTATTTAAGTTTTCGAACGCACAAAAGTTCAAAGTACTTTTTATATTTACGTTTTTTGTTTTTAATAGTTTTATTATATAGGTATTACGTTTGCTTATTTCTATTGTTTTTATTATTAGTGTTAATTTGCTCTACTAAAATGTTCAGTGCTCTTTCAAAAAGTCTCCAAATATTTACTCCTACCGTTGCGCGGCATTCACGATGCATAGCTTCTCTGTCTGGATTACCTGAAACTTACCAGATGCTGTATAAGACGTGCCGGGATTTCGCAGAGGGTGAACTAAAACCCAACGCAGCAAAGTATGATCGCGAGCACTTATATCCTGAAGAAGCTATAAAAAAAATGGGTGAACTCGGTCTTATGGCCATAGCTGTGCCAGAAGACTTGGGTGGAGCTGGTCTGGACTACCTCGCTTATGCTATAGCACTGGAGGAAATATCTAGGGGATGTGCCTCAGCTGGTGTCATCATGTCCGTTAATAACTCACTTTATCTTGGTCCAGTACTGCACTGGGGGACTGACAAACAGAAAAAGGAATTTATTACACCTTTCTGTAGTGGTGATACAATTGGATGTTTTGCCCTTTCGGAACCTGGCAATGGTTCTGATGCTGGTGCCGCATCGACTACGGCAAAAGATGCTGGTAACAAATGGGTTTTAAATGGCACTAAATGTTGGATAACAAATGGTTATGAAAGCAAAGCTGCCGTAGTCTTCGCAACAACTGATAAAAGCTTAAAACATAAAGGAATATCAGCATTTATGGTTCCTAAGCCCATAAAAGGTTTAGAATTAGGAAAGAAAGAGGACAAGCTAGGTATCAGAGGCTCTTCAACATGTTCACTAATTTTTGAAGACTGCGAGTTACCTAAAGAGAATATCTTAGGAGAACCAGGGTTTGGTTTCAAAATTGCCATGATTACACTGGACTCTGGTAGAATTGGTATCGCCTCACAAGCATTAGGTATTGCACAGGCATCGTTGGACGTTGCAGTGGAGTATGCATCAAAACGCATGGCATTCGGCAAGCCTATAATGAAGTTACAAGCCATACAAGCCAAGCTGGCCGACATGGCCCTCCGGTTGGAGTCCGCTCGACTGCTCACGTGGCGCGCGGCCTGGCTCAAGGATAATAAGAAACCGTACACTAAGGAGGCAGCGATGGCGAAATTGGCCGCTTCTGAGGCTGCTACGTTTGTGTCTCATCAGTGTATACAGATCTTAGGCGGCATGGGCTACGTATCAGATATGCCAGCGGAGCGCCACTATCGCGACGCTAGGATCACAGAAATATACGAAGGCACATCTGAAATACAAAGGCTAGTTATAGCTGGACAACTCGTTAAGGAATATGGTCTCTGATAAATTTGAGTATTGAATAGTCCTTAATATACAGTAATACCCCGAAGTTATGCAGGTGGGACCGAACGATAACCGCGTAAGTCGGGATACAGCTTTGCATATAAGCGCTTACTTTGTTTAAATGGGATCAGAGTCTTACTAACAATAGTATTGAAGATAAATCTACTAAAACTCTAACAACACTGACTGCTTGACAGAACGCACAGCCTAAACCGGTGGGTCTAGAAACATAAGATATTGCACAAAATTTCCTTATATTAGAATATTCAAAAATCCTTATTAGTCCACGACTACGTTAGGTGAGCACTAATAAAGATTTTTGAAAATTTGTCCCTTAAGGGGGTCTGGGGCCGGGGCTGGGGCTCCGAGCTACACTCGGGCGACAAGCTAGTATGTAATAATATTTTAACTATTTCAATATATTTAATCAATTGTAGTTATTTTGATGTAGATTTTATAAAATCTTTTTATTTAATTTTGCGCAGTATTTCTTCGTAGCACGATAATATTTTTTTTTTGTTGCCCGTTTGGCGCCGGAACATGAGCAGAACGTGGAAACGAGAATTGCGCGGCTGAGGGACGAAGCGGTAGGGGATAATTAAAAACTACGCAACTTCGAGTTCGCATATGTCGGGGTAGTGTAAGTCGAGGTATTACTGTACTCACAAATATTATCGAACGAATTAATTGTATTTGGTATATGTTTATTTGTAATATTGGCATTAGAATTTCATCTGTACATCGTCGACTAAATTATCCAAATTCCTATTCGCAAATCAAATAGTGAAGTTAATTTCGGTGTAGTCTGCAGAGGTCATAATCATAAATTCTCCAAAAAAAAAAATTACATGGTTTATGTAAATTTCAAATTAACATACCTTTATTTTCCGAATACCGATTGCATTGAATGAAAGCTTAAATGTTACTCCGATCTCGGAAGAATACACCACTGAAAGCTCCATTGTCTACTTTTCCCAAAAATTGTCTTAATGTCGTCCGATAGATAGTCAGTATGCGTTTCACTAAACCATATTTTTTTAAATATCTTCAATGTACAAGTATCATCCCTAGTACAGTTTTATTATATGTACCTAAGTATAGATAATTTGTAAAACAATCAGTATTGTCTCACCCCTTTAAAATCAATGAACTTACTATATTAATGTTAATGATGTGTGTTAAATGTGTATTTAATGTATGTATTTTACGATCTTCTGCTTCTATATTAAATAATACTATACAAGTAATATATAGAAGCAGAAGTTCGTAAACGCTAATTAGTGGGTGGCAGGCCTAATGTGCCATATAACGAAGTATCATTTATACGAATGTTTAACAAATTTAGAAAAAAATGTAAATAACTTATAAATAGCTTAAAATTGAATTTTATACAATGAGTTATTTATAAGAAACTACTCATAAGTTTTTTTTTAATATGAAATTCAATTTTTTAGTTTCAGAATACAATTTTTTAGCTCTTACCTTGCCTTTATTTCTGTTACACTCTTTATAATGAAAACATTTTAAAAAAAAATCACACTTGTTGCTTTGGTTCTTAAACCAGGAAAACCTGTATACTACAAGCACCAGAAACAACAGAAAACTGACCTATTACAAAATGCTTGTCGCTTGTAGGAATCAAACCTCTGCTTGTATCAATATAATGTTATTATCAATATAATGAATAATTTTGATATAATTTTGAACCTATGATTGACTAATAGAAAAAATTTTGAGATGTTAAGACTACATATAAAAGTATGAATAAAAAAAATCAGTGCATCAAGTGGTCCTCATGGATTTCGTGAATTTAACACATACCATAGATGGGATTATCTAAATATGGAGTGCAATATCCAAAAAAGTTAAACCAAACCACATTATTGGATATAATTATGTGGTTTATCCTGTGGTTCCTGGAGTGGTACTAAGCTAAGTAGGACCATGTTGTAAGCAAAGACCGTAACATGTTCTCTATTTAAAAACTAAATTATAATATCTTATACATTGATCTAAATATGTAGCTAATTATATTGTTAAGTAGACAAAGAATGTCAGGTGAATACTTGTTTTCACACGTGCACTATTTTCTAAGAAACAATGGTAATAATAAAAGTAAACTCCTGAATAAGTATTGCTTATTTAATTTTGTAGCGTTCACATAACATAAAAAAGTAAGATTTTATAATTCATATATCAATTAACATCTATATGATTGTGCTAGATTATTTATTTAGATTATATAATTATACACAAAGGAAAGGTAATAAAATTCAAAGAATTTGAAATAAATGCTCAATATAATAATATTGTCAACTTTGGCTAATACTTTATTTATTTACTGGCCTGGACCCGAGAGTGACTTGTCCATGAATTTCCTCTTCGCAAAACACAACCACCATTTACTCGGTTTGCCATCTTCTGTAGCAAATACCTTTTCACATACTCTAATTCCTGTCTCGTGTCTCAACTGCTGCAAGTACTGTCTCATAAGATCGGCCTCCTGAGGACTAGAAGGTTTAGCATAAACTGAGTTCAAAGGGAAGCCTGACTCTCCGGGAATGTCAAACTTAGATATAGCAAGCATATACATCTCTTGCTGGCCTTGATTCTTATTGGAGCACTTTTGCAGCCGCTTCAAACATTCTGAGATATATAGAGTCAAGTATATAAGTACCCTATCTGCATCTGACTTTATCTCATAAAACCTAAAGAACACATTTGCTTTGAAATAGTTAAGAGCTTCGTCAATAATATCCAACTCTTGCTTGGGGTCCGTTGGAGCGGGGCCTCTGTAAGTTGTTCGTAGGGGCAGAATAGCTAAATTACCAATAGACTGGGTGTAGTCTGTTAGCGTTGAGTGATAAGCCGGCATTGTTATTGAATAATTTGCACACTTTTCCAATACAAGCTGTATTAATATATTCAAAATCTTCAAGGGCTAATTTCAAACCATAAAATACGTTTTCCTTCGATTAACAATCACTAATATATAAAATGCGGTCTGCAAAAAATAATTTGCCACAATATTTAATTATACTTGTTATCATATATCATATATAGAACATAAATATAATGAATTATGAATCACTACAATCAAATTATTACAATATTAAGAACAATAAAAAAATTTAACAGATTAATAAAAAACCAATGTCAACTATATTTATTAGTCTGTGAAGTCAACGTTTTATTTATTTGTTTATCTATTTTC

**ECH:**

>SinsECH1

GCTCTGGCAAAATCACAGATTAATTTATATTTCATATTAATCTTAAATAAATCGTGTTATTTACAAAAGGCGCTATTTATTTTTAAATTTAAAACATGAGGCTTATAATCAGAAGATTGGTTTCCGCACACACTTTATCTCGACCTAAAATAAACGAAGTTCGACAGTATTCCAATAATGTTAAAAATAAAAACGATAAACTAAAGAAATCGGAAGAAAAAGAAATACGAAAAAATATTGTAGTTGAAAAATATGGTGGTATAACCATATTAAATATAGACCGCCAAAAAACACGCAATAGCCTTGATGAAGCAACTCTTCGAGAGATGGCAGCTGCCATTGACGCCTTTGACAAAGATGAGCAAGCTAAAGTGTTGGTCTTTAATGGAGAAGGTGGATCATTCTGTTCGGGCTTTGATATTGATGAGATAGGAGAAAAAGGATATAGTACACTTAAGGATGCTGCGTCTCGTCTACTCCGTCGGCCGTTGTGCGACAAGCCAACAATAGCGGCAATGGCAGGGTATGCCGTCGCAGAAGGGTTTGAATTGGCGCTGACGTGCGACCTGCGCGTCATCGAGGACACGGCTGTAATGGGCTGCCTCGGCAGGAGGTTCGGCGTTCCACAAAGTTTGTTCGGGGCTCGGCGTCTGACCTCACTGATTGGCTTATCACAAGCGCTGGACTTGCTTATGACTGGACGTCTTATCAACGGCGCGGAGGCCAATCAGAAAGGGCTGGCTTCTAAACTTACTGCCACTGGGACTGCACTGGGCGATTCCATAAAATTGGCCAAATGTCTAATGAAGTTCCCACAAAACGCGCTCATAATGGACAAGTTGGCCGCTGTAAATTCACAGCTGAACCCGAACAGTGAAGAGAGTATGCGCGATGAAGCCGTCATGACTAGTTTACTTGGTGGCGCAATTGAAGATGTCAATGAAGGAGTGAAGAAGTTCCAGGAAGGTATCGGAAAACATGGCAAGTTCTATAACCTGACTGAAATTCCTCTCAAGGATTGGGAATTGGAAGAAACTTTGGAAGAGATCACGGTCAAAATAGATAAAGATGAAAAGAAATCTTGAATCTGATCGTGTGATAAAAACAAAATACCTAGTCATTAATCGCTTTTGTATTTAAATGGAGAATATATATAAAAAAAAGTACGATTGCGTCGAGCTGAACAAATTAATCGAATATTAAAAAA

>SinsECH2

CCATTTAGTCGCAATATCCATCAGTCTGATTGATTTCACAATATTTCATTTATAAATTCACAAACATATTTTTAAGTATATACAAAATATTATTTGTGGTTCCAGTTAGCTTTTCTTTTTTCAATGAAACTTTTTATTCCTTCTTGTCCATCATCAGAATTGATGTTTTCAACCATTACATTTTCACCCAACTTATAGGCATCGAATAAATGTAGATCGATTTGTTTACAGAAGAATTCTTTACCTAAAGTGATAACCCTGCGACTTTTGAGTTTGATTTTTTCGATTATTTTGTACACCTCATCATCCAGTTGATTGGCTGGCACTACTTTTGTGACGAGACCACTGATATATGCTTCTTGCGCTGTGATAGTGTCACCTGTTAATAGCATAAACAAGGCTTTACTCTTTGGTATGACTCTACCGACTGCTATTCCTGGTGTTGAACAAAATATTCCAAAGTTAGCTCCAGGAGTGGAGAATTTACTTGTGTCTGTAGAGACAACAATATCACATGTTGCGACTAGCTGGCAACCTGCAGCAGTGGCAAAACCATTTACCTTTCAAACAAAACATTGTTTAAAACTTCCAAAATCTTTTTTGGAACGTATTACATAAATTAAAATAAAAGAAAAGTAATTACTTTAGCAATCACAGGTACAGGACTTTCAATGATTGCTTTCATTAATTCCGTTGCTTTTTTAAATATTATTTTATGTTGATTTATCCCTGAATTTGATTGCTGGAAATGAGAATAAAATAAGGGTGACAAATAAAGAGGAAAGTTTATACTAGGCGAAAATGTTTACAGTGGATTTCAGAGCAGGTATTTTTAAGAATAATAAGTGCCTAGATCCATCTCAGAATGGTAGGAGAGGAATAAATAAGGTAAATTCTGTTCAACTGTCTCAAACATAGAATCAAGAGTGTAATGTCTATGAGTGACAACTAAATTATTATAGGGTACATACTTACAAATTCTTTTAAATTATGACCTGCAGAAAATACGTTTCCTCTAGCAGATAACACTATTGCTCTCAATGATATATCTTCTTTATTTGTGTTTATTCCGTCTATAAGGTAATTCATCATATCCAATGACAGTGCATTTTTTGTTTTTTCATCGTGTAAAATGATTTCTCGTATCCCATTACTTTCATTGGTGATAATATATTGATTATGTACTTTCCGACAAAGAATTAATCTTGAACATTGTTTCTTGTACAAAGCAAACATTATCGGTCAATAATTATATATTATTTTTGAATCGAAACCAATTAAAATATATTGTTTTACTACTTTCATCAAAACAAACTCTGTG

>SinsECH3

GTTTTTTTACAAAAATGCAATATAAAATACAACAAAATGTGATAGTAATAGAGTATAATATAATAATATTATAAAATCTGTAAAATAACAAGTGTATATAAAATATCAATAAATCTATTTATTATCATGCAATTGTACTTATTGGTCCCCAAACCTCTAGAATATATTTTAAAGTATTATAAAGTCCTTACTAATTGGATGAAAAGTAAAGCATTAAAGGCACTCGTCAATCAGTTCACTTTTGATATTCAATGACCTTCATACACTGGCTTCCTTTTCTCTATAAAAGATATCATGCCTTCCTGCCTGTCTTTTGTGGGAATGTTTTTCTCATAGAACTTCTGTTCGACTTCATATCCATCCTTTATGCTGAGCTGGATACCCTCATTGATGGCTTGTTTAGCACACCTGAGTGCTATGGGGGCATTTGGTAGGATTTCTCTGGCCAAGTCGAGTGCCTTCTCAAATGCTGCATTCTTGCCACTGTTTTGTTCGACAACATGGTTCACTACACCTAATTCTTTAGCTTCTTTTCCATTTATTATTCGAGATGTAAATATCAATTCTTTTGCAATATTTGGATGTACTACTCTTGGCAGCCTCTGAGTACCTCCAGCACCAGGGATTAATCCCCGTCCCGTCTCCACTAGACCAAGCCGTGCAGTTTCAGCTGCCACTCTGATGTCACATGCCAATGCCAACTCCAGGCCCCCACCGACAGCAACACCGTCTATAGCAGCTATGGTGGGCATCGGCAAATCTTCTATTTCAATGAAAGTACCTCTGAGTCTACGAACAAACAGAGCTACCTCCTCGTCTGACATGTCAAAGCGTTCCTTTAGATTAGCTCCAGCACAAAATATTTCAGGCACCAAGCTGTGTAGTATTACAACTGAAATTTTGGTGTCTTCTCTAAGGAGTTGGTTGAGTTCTCTCATAGCGTCGACTACCTCGAAACTGAGGGCGTTTCTATTGGCGGGGCAGTTGAGGCCGTAAAGCGCGACGCCGCGGTCCGCGCCGGCCAGCTTGCGGAATACCACAGGCCCCGCGCTGTCGCGCTCCTGTTGCTTCTGTGTGGACAGAGGCCGAAGGTTCCGCCCCTTCAGCACCCATCTAAACGTATTCAGCCTTGGTACTATCATTGTTATTTCACGGGATATAATCAATTAACAGATGTTTGAAAGTAATTAATACGCTCGGTCTACTTGTCGCACTCGGTAATATAATCTATATAGGCGTTTGTTTTTCTTAACAAGTCTTATCATTAGAGATAAGGTTAGTAGCGCACGGCCGCGGATTCTACAAAGAAAAGAACTATACAAGTATTTTCCATTGTTTACAATGCGATGCGGATGCGGAACTTTAAAGTTTAAATTAATTATTTAATTTTGACAGTTCTGTAGTCTTCTTTTCACTTCAGGGTCTTTTCACTTCGAACA

**HAD:**

>SinsHAD1

AAGGCTTCATATAGCCAGAGCCGAAAAAACTAATTTAAAAAAACGTGCTTATAATGGTTATATTCGTATTTCGTATAAATGTATAATGATAATGTACTTTGTGTTCACTATATCTAAATTTTTTATATTTTGTGTCGCTTATCTACAATAATTTCCTGTTATCATTTATTACGGTCAACTATCTGTTCATGCCTGTTACATTAATTGTTTATTTAAGTTATTATTTTTCATAGCTGTGTGTGTATAGCATAGATTAGAGTTTTAAAAACTTTAATTACTTGGGTGCCTATCTGTTATCGCAATATTCTATATAACATAATTTTTTCAAAATGTTTAAAGGTCTAGTAGGTTTAGTAACTGGGGGAGCGTCAGGGCTTGGCCGTGCAACAGCAGAGCAGCTCCTTAAACAAGGTGGTCGCGTTGTAATATGTGACTTGCCTTCTAGTCCTGGTAAAGATGTAGCCAAGCAATTTGGTGAGAATGCTGCATTTGCACCTCTCGATGTGACATCCGAAGAAGATGTAAACAATGCATTACAGTTAACTCGTGACAAGTTCGGTCGGCTCGATGTACTAGTCAATTGTGCAGGAATTGCCACTGCAGTTAGAACTTATAATTTCAAAAAGAATCAACCGTTTGATCTGAAACAGTTCCAGAGAACTATTGAGGTCAATCTAATAGGCACATTTAACGTGATCCGGTTAGCAGCTGGTATGATTGGAAAGAACGCGCCAGATGCAGACGGTCAAAGAGGCGTTATAATCAATACAGCAAGTGTGGCTGCTTTCGATGGACAAATCGGTCAAGCGGCATACTCTGCATCAAAAGCCGGCGTTGTTGGTATGACGTTACCCATTGCGAGAGACTTTTCTAAAGCAGGCATCAGAGTCGTTACTATAGCGCCTGGTCTGTTCCGCACGCCCATGATGGAACAACTCCCCGAACCGGCCATTGCGCAACTGGAAGCGTCAATACCATTTCCATCGCGGCTCGGTCATCCTAGTGAATTCGCTCTGCTCGTTCAAAGTATCATACAGAACCCGATGCTCAATGGTGAAACTATACGTCTAGACGGCTCGTTAAGAATGCAACCTTAGTTTCTTAAATAATAGTCGTGGATGAAATAGAATGGGTTCAGTACCTCTTTGTTTGAAATACAATGAATGTAAGGTATATAATTGTAATGTAGAAATATATTGGGTTTTATAAAAAAA

>SinsHAD2

GACAGTTTTCATTTTACTATAAAATTACGCATGTATTGGTATTGCAACAATTTCTCAAAATTTCAAGTCAATAGATCGACTACTTATCGAGTTTTGCAACCACAAACGTACCGAGAGAGGCCGAATTTTTATATTATATACATGTAAGATTACTTAACTATTTATATACATATTATACTAATATGTATTAACATTTAAAGAGGAAACCATCTTTGAGCACCATCTAATCTTATAACTTCACCGTTTAGCATAGGATTCTCAATTACACTTGTAACTAAATGCGCAAATTCTTCGGGCTTGCCTAATCTTGAAGGAAATGGTATCATACGTTTAAGAAAATCTAACACTTTCTCCGGTAAATCCGACAATAATAATGGAGTTTCAAATAGTCCTGGCGCAATAGTGACTACTCTAATGCCCTGCGGTGCTAAATCCCTCGCTATGGGTAAAGTCATACCAACAATGGCGGCTGTTGAAGCCGCGTAAGCTGCCTGTCCTATGTCTGCTTCAAATGCGAATGTCGAAGCTGTGTTGACAATAACACCTCTCTGTCCATCGACGTTAGGTTCATTTTTGCCTATCACACCTGCAGCTAATCGTATTGCGTTAAATGTACCTATTGTGTTAACATTTACACATTTGATAAAATGATCCAAATTACATGGTTTGTCTTTATTAAAATTGTACGTCTGGTATGCCTGAGCAGATCCTGCACAATTGACGAGTGTGTCAAGGCGACCAAACTTATCACTAGCTAAGTTTATTGCTTTATTTACGTCTTCTTCAGATGTGATACATCCGGAACTAGCAATCACATTTTCACCAAGTCGTTTCGCCACTTCCTCCGCCCTTGTACCCTGAATATCTAAGATCACAACCTTTCCACCGCTCTTCACTAACCTCTCAACGGTCGCTTTGCCAAGACCTGATGCACCACCAGTCACCAAACTAACCATACCCTTTAACATTGTTGTATTTCTTAGAAATTCTGTGTGAAAATTATATTTTTTATATAGTAACTAAAGTAAGCGGTAAATTTGTTTTTACAGATTATAA

>SinsHAD3

TTTTTTTTTAATTTAGAAAATATAGTTTATTTATTGCACATCTTAAAAACATACATAATTAGTGTGTAAAAAGTCACAATATAATACGCTTAAAAACTTCAAAACATAACTTCATGAAGTTGTAAAACTTTCCAAGTAAATCACAGTCTTATTTCTTGTCATATTTGTAGAAACCTTCGCCTGTCTTCACGCCGAGTTTGCCTTCGGACACCAATTTATTCAACAGCGGTATTGGGTTGAACAGCGGTTCTTGGGGATATTTCTTGTGCCAACCGTCCAAGATGAACTTGTTGGTGTCCAAACCCACATAATCGGCGAGCTCTAAGGGTCCCATGGGATAGCCTGCTCCTAATTTCATAGCGATGTCGATATCGCGAGCTGACGCGTCACCTCTCTCAAACAATCTAATAGCCTCTGCTATATATGGTACCAGCAGTCTGTTGACCACGAAGCCGGGTGTATCCTTGCATGTAATGCAGGTCTTGCCGACGGCCTTGCCCCATTCCATCATGGCTTTGTAGGTCGCTTCCGATGTCTGATCGCCGCGAACCACTTCGAGGAGACGCATCACTGGCACGGGGTTGAAAAAGTGCAGGCCACCAAATTTATCTTTTCTGTTGACAACTGAAGAGATCTCATTGATTGATAGAGATGATGTGTTGGACGCGAAAATAGTGTGGTTCGGAGCAACCCCATCCAGTTGCTTGAAAAGTTTATGTTTCACTTCAATGTTCTCAACAATGGCTTCCACAACTAAATCTGCATTTTTAGACGCGACCCCAGGGTCAGTAGAAGTTTTGATTCTACCAAGAGACTCGGTAACAAATTTCTCGCCTTCCTGAGGATTATCCTTGTATAATTTCTTAGCTACACGACCAAGGTTCGTGCCAATTGATTTCTGCGCTTTCGCTAGTACATCTGAACTGACGTCTACAAGAGTCACATTTTGACCAGCTTGAGCAGACACCTGGGCGATACCAGAACCCATAAGGCCTCCTCCGATTATAGTTACATTCTTAATTGCACTTTGAACAGCAGCAGAACTAGAAAAGTTTCTAGCGATAACTCCAAATTGAATCATTTTTATTGCACTTTTGCTTACCTACATAAAATTCTTAATTAAAACTTGTTTTTTTTTTTTAATTTACAATAAAATAATTGATATATTATGTGTGTAAGACGTTTTATTCTATAAAAAAAAGTTAAAACATTTAATTCCAAAGTTCAATCTACTGCCCAAAATTAAAGTGGC

**KAT:**

>SinsKAT1

TTTTTTTATTTGCTCGAGACGTATTTATTATCATCACCCTGTTCAATAATGCTGAGAATCATTATGAAAATGTAATATTTCACATGACTGTGTATAAACCTATAAATATTTTTTGTTATTTACAAATAGGTATAATAAGTGTTTGTAAGCATGGCATTAACGTGCAAAGGAATATTCATAATTGGCGCGAAACGGACGCCATTTTGTAATATCGGTGGTCCGCTGCGAGAGTCGGCAGCATCGCACGCTTTTGCCGCCGCGGCGCAGCACGCGATACGTTCGGCGAATCTAGACGCGAACTTGATCGATAACACGGTGATCGGGAATGCACACTTTCTGAGCCAATGCGACGGCGGTAAAACTGCGAGGTACTGCGGCCTCTACTCCGGAGTGCCCTTAGACAGGCCGGCGTTGGCGGTCAACAACACTTGCGGAACTGGTCTCCAAGCAATAGTCACTAGTTCTTTGGATATATTGACAGGAATGGCAAAGATATCACTTTCTGGCGGCACAGAAATTATGTCTACCCTACCATTCCTAGTCAGAAATGCCAGGTTCGGCACAACTTTGGGCACGGCTTACAAACTGGAGGATCACATCCAGAAGCAATTTCTGGATTCCTATACAGGGTTGACGTTGCAACAGATAGCCGATAGAGTGGCGAAGATATATGGATTTAAAAGAGAAGAATTAGATGCCTACGCTTTTGATAGCTATTTGAAAATGAAAGCAGCACAAGAAGCAAAGGTATTCGAAGATGAATTGACGAAATTCACAGTGAATCTTAAAAAGAAATATGTCCTTATTGAGAAAGACGAGATAGCCAATATAAATATTAGCGCTGAAGAATTAGGACGTGCACAACCTTATGACAATGAGTCAGTGATAACGACGGGAAATTCCGGTCTCCCAGCGGACGGCGCTGCCGCTTTGCTCCTGGCATCAGAAGAAGCAGTCGCCAGACACGACATCAAGCCTTTAGTCAGAGTGGTGGGATGGGCTTGCGTAGGTGTTGATCCAATTGAGACAGGACTGGGCGCCCTGGCAGCGATTAAGAAGTTGATGGTAGTGACCGGGAGGAGAGTGGACGATGTGGATATATTTGAGATAAACGAGACGTTCGCTTCGCAAGTTCTAGCAACCAGCAAAGAGCTGAAGGTGGACCCTGAGAGAATTAACATCAACGGCGGAGCTATATCCATTGGCCACCCGGTGGCAGCCACCGGCGCCAGGATGGCTACGCATTTAGTTTATCAGCTGAAACGAGGAAATTTGAAGAATGGAGTCGCGGCTTCAAGTTGCGGCGGAGGGCAAGGCATTGCGATCATGTTGGAGAATGTATAATATTTGTGTCTGCTGCGTACAGTGTGCAAATAGATGGCGTTGATTTTTGAA

**FAR:**

>SinsFAR1

CTGTAACCATGTCATGAAGATAGTGACCTAAATTGTGAACCGCACCAGTGTTCAAAATCCACCAAAATAGACAGCCATAGAAGACACACTGCACCAGCTTGTCCAAAACCAAGATTCTACGATAATGCGCCCTAGCTTTCGGCAAGTCCTCGTCTTTTTCATTCAACAAATACTTCCGCCCGCCCACTATACACATTTTGAAGTATTCGTATTTATCAATGTTTTCTATTTCGTAATAGAACTCTGTCTTGTCCTCTTCCTTTATACGGGATCTGAGCATGACGAAGTTCTTATTGTCGAAGGTCCACTGCTTGGTGACGTAGTAACGGAGAGCCAGGTTTGCTATGTAGATGCGACGCTGGAGCTTCACAAGCATGGGCTTCTTCCCGAGGAGCCATAGAACAATATCGATGAATATAGCCGGCAACAAATGGAGCAATAAAACCTTAATATAATGTATAGTTTTAGAAGTAGTAATAGATCCTCCTAAGTTCCAGAGCATATCATTAAGCGGCATGTAAGCTCCTATCTTCTTTCCCGTTTCTACCAATTCCCCCATTGTCATGGTATTCAAGTTTCCCGCGCAACAATTATATATAGGCACGTCATCTGTTGGCTCTAAATCCTTAGTCCCTCGAATCCAGGCGGCGGCTATTATGTTCTTGATGGCTATGTCCACGGGTATATAGTCGGCTATTAGGTCCGGATCCGAATATATACTACGCATAATACCTTTTCCACAAGCCACTAGAATACCAACCGGTCCATTAAAATTCTCGATCCATCCCGCGAAAGGATCGGTATAGCTAGAGATCACAATAGAAGGTCTTATGATGACGACAGGTAACTTCCCTTTCTGCTCGTACGCGACGTGTTCAGCTAATTGCTTGCTAAACGTGTAAGTGTTAGGTATCTCGCCTAAATATTTTGGTGTGAGCGTCCGTATGATATGTTCGTTTACATTCTCGCATACAGATATTGTGTCTCTCCAATCGGCGTGGGCGGGATACATTACTTCTTCTATTACCTCTCTGTTTGTGTTCGAATATGATGTCGACACGTGAATCAGTGCAGATAAATTACTGACTTCGTGCGCCAAATCTATAACGTCTTTAGTGCCTTTGAGATTAAGTTTCACGGCAAATGTCAATGGATCATCAAACCTTACACTAGCGGCCACGTGAAATATAATGTTCACTTTATTGACGATTGTGTCTCGATCTTTTTGAGATAACCCTAATCCAGCCTCCATGACATCACCAGACATAACGAACACCTTCGACTGGTAAACTCCTGGCTTCATTTTCCTTAATCGGTCAAAACATGCTGAAGAATATAATTCGGTCAATCTCTCCTCCGATTTGACACCCTTCTTGCTTCTCAGCAATAAATAAATTCTGTCCAGTTCTGTGCAAGAATATAACAATTTCTCGATAAGCACCTTTCCCATGAAACCTGATCCTCCAGTGATCAAAATAGTTTTCCCTTTATAATATTCCGGAATCGTGGGCACCTTACTCAAATCTCTTTCTTCCAAAAAACCCATTGTTGTAACAAAAATCAAAAACTCAAATAAAAAAACCTAAAACTTTATTAAATCAAAACTTAACGACTTTAATTAAAATCTCTACAAAACTGCCCGTTCATGGGCACAGAATAAAAACAAAACTTTAAAATTCACTTCTATTGTTTCAAGTTTAAAAAAAAATGTAAACGGTAGACACTTTCCTGTACGTATTTATATGTGGAAATTTGTGGAAAAAAATAATGAAATTTCGTTGGTCGGTGCGGTGCGTCTGCGTCGC

>SinsFAR2

GTTTTTTTTTTATTTTTTTATTAATAATTCAGTGCAGAGTGCGTGACTTTTTAAAGTTTAAATATTAATATAATATCTAACTTAACGTTTTTAAGAAGGATTTTAATTAGAAAAAAAATGTCACCAGAGAAGCACTAACAATAAATAAAACGAAAACTGAAACTCGAAAACAAAATGGCGGCCACCAAACTCAGTACAGTCGGAAACAAAATTTCCAACAAAGAATCTTTGGAAAACAATAAAAACAAACAGAATAGTTTAACGACAGATAAAATTGTTTATGCGAACAACAAGATTGGCTTGAACGATATTTGTGCCGACTGTACTAATATTGATAAAATTTACGGCGTCGACGAAAATATGAACAAAACATTGAGGTTGTCCGAGATTCAGCAGTTCTATCGCGGGAAGAACATACTTATTACTGGAGCCACTGGCTTTCTTGGCAAGATCCTGATGGAGAAACTGCTCAGATGCTGTCCTGGTGTTGACAATCTGTACCTGATCGTTAGACCGAAGCGTGGGAAAGATATCTATTCGAGGGTGGAAGAGATATTTGATGATCCTGTGTTCGATCGTCTCAAAGAAGTGATGCCAAAGTTTAGACACAAAATCGAAGTCATCCCTGGTGACTGCGAAGCAGAGGGATTGGGACTTTCGGCGAGCGATAGACAAATTATAATTGAGAAGGTGAACATAATATTCCATTCAGCTGCGACGGTGAAATTCGACGAGCATCTCCGAGCAGCTCTGGTGACCAACGTCAGAGCGCCCCTGCACCTGCTGAAGATGGCCAGAGAAATGAAAGGTCTCAATGTTTTAATGCATGTATCGACGGCATATTCGAACTCCCACCTTTCTTATGTTGAAGAAAAATTCTATCCTTGCGACGCGGACTTCGATCAATTGCAGAATATAGTTGAAAAGCTCACCGATAGAGAAATTGATGAGTTATTACCTAAAGTATTAGGATCATGGCCAAATACATATACGTTCACGAAAGCGTTAGCGGAGAAGGAATTAAGGGAGAATGCTGGCAACACACCTTTGGGCATCTTCAGACCAGCTATAGTAACTTCAACAGCCAAAGAGCCATTGAAATGTTGGTTGGACAACATGTACGGGCCTACCGGAGTGGCCGTAGGCAGTGCAACTGGTATCCTAAGAGTAATGCGTTGCGACGAATCCGTGACGGCAGACATAGTCCCCGTGGACTACGTGGTCAATTGTCTGATGGTAGCTGCAGCTAACGTTTACAAAGCTTATAAACAAAGCCCACAACCTCGGGAACCACTAATATATAATTATGTGAGTTCAGTTGAGAACAGAATCACTTGGGGTGATTTTGTGTCACAAAATATGGCCAGGATTGACAAACATCCATTTTCTAATGCTGTTTGGTATGTGTCCGTGACCCTCAGTCGATCACCAATATTGATTCAAATCTACATATTCTTCCTGCATCTGATACCTGCTGTATTGATGGATGGATTAGCTGTTTGCATCGGAAGGAAGCCTAAAATGTTGAAAGTGTACCGTAAAATTCACAAATTTTCATCGGTACTCTCTTACTTCTGCACGCGAGAGATTACATTCTGTAATCAACGAACGAGAGAGCTATGGGACAACACTTCTGAATTGGATAAACAGCTATTCCCGTTCAGCATGCAAGAAATAGATTGGTCGGAGTTCTTCGAGCATTACTTAGTTGGTCTCAGGCGGTATCTGTTCAAGGAGAGCGACGAAACGCTGCCGGCGGCCAGGATCAAATGGAAAAGATTATATTATCTGCATCAGATAGTAAAAATGGCTTTCTTCGTCCTGGTCTTCTATTGCTTATGGTCAGTTTTAAGTGATCTATGGCGAC

>SinsFAR3

CACGAGTGATCTTATAAGGGTTCCTTTTTTATTTGGAAGTACGGAACCCTAAAAAGATCTAATTGGGTAAATCAGATCTTTCGAAGTAGCGTCTAACCAAGTATTTCCTCAATAAAAAAACCAAATTATCGAAATCGGTACTTATTTGTCGAAGTTACGCCTATTTTAACCTATTATGTTATACACAATATACCTCCTATTGATTTGAGAAAAAGTTTACATAGAAAAATCTCATTATACTTAAATCATATTAAACGGACGGTTTACACGACTAAAATTGATAACAAAAATCACTTTTAACTTATCGAATAGTAAATTCTTAATTAATTAAGTAGGTACATTAATTTAGATTTAAGTCCATTAATCATAATTAAGTTTGAATTTCCCCAAAATCAACTATATTTAATACTTAATTATCCCAAAACCAATCTGAAAATGGTCTTCAAAAAGAAATATAACAGGTTTAGGAGAAGAGCAAAGGTGGTTAATTTCAATATTAGATGCATGTAATATAATCTGTTCCACTTCTTTCTGGCTTCAGGAAGCGTTGTTAAATCATCTTTCAAAAGATACACACGAAGACCAACTATGAATGTTTCAGCTAAATAATCCCAGGACATGTCAGCAATATTGAACGGAAACACGGTCTTGTCAGCGTCAGAGAGGGAGTTCCACATCGTCATGACGCCGTCGTCATTAAAGCGCCAATCTTGCGTCGAAAAGTAGAATAATGTATTCGCTAGCTTCATCACTTTATAGTAAAAATGGAGCATTCTTTTATTGCCTGTAAGAGCTGCGTAAGTGTCGATGAGCAACGCAGGAAGGTAATGCAGGAAGAAATTGTAGAAGAGGAACATGTAGTAATTGCTCGTTGGATTCAAGCCGTAGTACCAGACAGCTTTCGTGGTTGGCTTGTCGATGCCGTGCTTGTGGTTGATCTCGATGAAGCGACTCCACGTGACCGGGTTCCGTGCGCCCGACACGAAGTTGTAGATGGGTATGTTCGACGTTTGGTTCTCCTTGAAATGTTTGGCCGTGTACCACGCGGAGGCCAGAATGGCATTCACCACCAGGTCTACTGGCACCATGTCGGCTACTTTGTTGACGTTCATGTACATTGTACGAAGAACCCCTGTTCCGATGCCAATCATGAGACCCGTGGGACCATACACGCTATCCGTCCAACCGCGTACGGGTTCTTCGTACGTTGATATCACTATGGAAGGTCTTACGATACATATGGGCAAACCCTCGCCATACTTCCTCACGGCTTCTTCAGCAACCGCTTTCGTGAAGCAATACGTGTTGGGATGCTTGCCAAGCAGTTTTGGTGTGATGCTGTCCAGCAAGCTTTCGTCGATCCTCGCCACTGCTTCCAATGCGTCTACGGTCATCGGTGGTTCATAGAATTTCTCCTCGATGTACTTGACGTGAGTGTTCGAGAAAGCCGTTGAGACGTGCGTGATGGCTTTCAGGTTGCGACATTCTTTGGCCAACTGGAGTACTTCCCTCGTGCCGTGGACGTTTATACCGACTGCCACTGGCAATTTCTCGTCAAACTTCACTGTGGCTGCTGAGTTTATTATTACAGTCACCCTGCTGGCAAGCGTTTTGCGATCTTCGTCACTAATGCCCAAGCCGGGTAACTCCATATCACCAGCAATCGCAACCACTTTATGTATACCCTTCGGATTTTCATCGTGTGCTCTGTGAAATAGAAATTCGTCCAGCAATTCGTGAAGGCGAACTTGAGCATCCTTCCCTTTCTTATTCCTTATTAACACGTATATAGTGTCGACATCTGTACACGAACGAAGCAACTTCTCGATTAACACTTTACCCAAAAACCCAGTACCGCCAGTCAAAAACACACACTGGTCCTTAAAGAACTGCTGTATTTCAGTTTTGGACCCTTTACCCTTGGTAAGTCTCTGTTTCGCCTCCTCCTTGGCATCAGATCTGTCTTCCTCCGCGTAGTAGTTGTCCAAGTTGTTCGCCGACACGAACTCGTGGTCTTCGGAGAAGTTCAGGATCGGTGCTGATGGATTCATCGCTTCGTTTTCCACGTCAGCTATACGTACAGAAGTTTCCAAATCATGTAAATTTTCCTGCAACTCCTTGCTCAAGCTCCGAATGTGCTCCAATTCCTTCAGCTGCTGTTCCGAAGGCTCCCCATAAATGTCCACTTCTATCTTTTCCCCTTTGCTTTGAGCTGATACCCACCTCTCCATTTTGTCACGCGTCAGCGTATACTGTCTCAATGGACATTCTTCTAATGAGTGATTCATTGTCCTTTTGGGAAGAAAGATACGCAAACTATTTCAACAAGTTCTTA

>SinsFAR4

GTTTACTTCGGTAGGCCTTCGAGAGAGGTTCGGCAGCTCGAAAAGCTGTGCAACGAACATCGCTCCACATCGCTTGCTACAAATAAAACAACAAGTTCGCCATGGCGTCTACTAAATCAGTGAGGGACTTTTACAAAGGAAGAAACATTCTTATCACCGGTGGTACAGGATTTATGGGAAAGGTTTTGATTGAAAAACTTCTCTACTCAATACCTGACATCGGCAATATTTACATTCTCATTAGGCCCAAGAAAGGAAAATCTATCAACCAAAGACTTGAAGATATGCAACGGCTCCCGTTATTTGATCGAATTAAAAACGAACGACCTAGTACATTACAAAAAATGAAACCTTTGCAAGGAGATGTTTTATTTGAAGATTTGGGATTGTCCAATCTAGATATAGAAAAGTTATTGAACGAGGTATCGGTGATCTTTCATTTTGCTGCAACTTTAAG

>SinsFAR5

AGTTTCCACCACATGCCTGCCTGCGTCAATCAGCTCCTTCCAAGTGACACGTACTTCGTCTGATGAAGTCAAATTCATTACATCGTTTCGACAACTATTCTCCAGATAGTCCCAAGCGATGGTCATGAGCGCGTTCACGACGTAGTCAACAGGGACGAAGTCTGCGTTGGCCTGGTCGTTACCGTACATGGTGCGGATCACGCCTTTACCTGCGCCAATTAGGAGTCCTACTGGTCCGTTGATATTGTCTGCCCAGCCTTTTACAGGATCATTCATTATTGGAACAACAACAGACGGCCTCACAATCACCGCAGGTATGGAGTCCATGGCTTGCTCGACCAGTGATTCGGACAGCGCCTTACTATACGCATATGTATTTGGATAAACGCCTAAATATTTAGGTGTTAAAATATTAATAGTGGCGTCGTCAACAGTTTCGACCATGTTGATTACTTTCTCAGGATCTGATGGCGGTGGATACAGTTCTTCTTCCAGCATCTTCTCTTCTGGATGACAGTACGCCGTTGATACGTGAATGAAGAGCTCGAGCTGATTGCATTGCTTAGCGAGCTCCAGCATATATTTAGTACCCCTTGTGTTGAGTAGCACAGCGGTTTTGATGCCTTCGTCAAATCTAACAGTCGCAGCAGAATGCACGATGATATTTGCAGTCTGACATATAGCTCTGTCACTCTCACTCAATCCGAGGTCTCTTTCGCTCATATCCCCTGACACTGGAGTCACCAGTGAAAGAACTTCATCTTTGCTTCGAGACGTTATCAAGGAGCTGAATAACGGATTATTGAAGATCTCTTCAACCCTTTGTTTTGCCGTTCTACCCTTCTTTTCTCTGATAATTAATATTATACCGCCTATTTTTGGACATAACCTCTCTTTTGCCCACGATTTCGTCCGCGTAAAACTCGTTTATCGTTATTCCCTTAGGAACTATCAAATTTTCAGGGAT

>SinsFAR6

ATGCGTTTATTTACCCCCTTTACCTATTATATATTCAAAGATAACTGATAATAATCAAAGACTCCGACGCGCGACTGGAGCTTACTCTCTAAGTTCTAGAGTAAACTCCAATCGCGCGTCGGAGCTGAGACACACATTTTTTTTTTTAAATACAAAACTTGCGTTTTATGTTAGAAACAAATAAACTAAGCAATTATCGCTTTGAGAAATTACGCTCATTGGAATATTGTATTGTATTGAGATTTGGCTCAAAATAATATCAATGTAAAAATATATAATGTCATATAAAAATATAAAATATAACGAGAAAAATTGCCAAAAACTTATTTTAAAACATTTCATATGTAAACTTGTTTGCTTATTAAATGCCTTTGGATTGACTAAGAGCAATACTACTACTTAACTATATATTTATATAATAATATTGAGCTGAACTGAAATAAACAAAATCATTCAAATTTATTTATTTTATTTAAGTTCATTAAATTGATTACAATTGGGGCCGTTTAAATTACAATATAAACAATCTCCTTTTACAAAAAAAAATATCGAATATGTTCATTCAATTGTGTAAGAATATTACCAATATTATAGACTTGAGCAATCTGCTCAAATTAATAAAATTAGTTACTGGTTATGTTATTAGGATCTCCATTTGAGATGGCGCTTAGCGCCTTGAGCGCAAAGCGGTATGTAGTCTGGCCAGTGGATTTCAGAGACAGTGTATTTGAATTGTTCACGATCTTCAGGCGAGAGTGTGGAAGCAAGTTTGCGAGCCTCACGCGTCCTCATTGTCCACGTGTTTGTCGTGAAATACTCTGTATTATCTCTTATATCATAGAGTCGAGCTAGTAACTTCTCATGCTTAGGTGGTTTTCCTCTAACGCGTAGAATTAAATCGCCTATCTTGCTAGGTAGTGCATTTCTTATAAAGAAGTTCAATTCATTGTAATATTTATTTGTAGTGTACTTTGGTACTGGATACCTGGGATGTCCAAGTCTGACTGATTCGGCAACGAACAAATCAATGATTTCGCCGAAGGTGATAGGGTTTGTATCGCTGCTTGTACAATTGTATACAGCCACTTCATTTGGTTTTCGTCCTTTAGCAGCTGCGACAATTGTGAGGTTAGTGATGTAGTCCACTGGAATGATGTCGAAAGTGAATCTGGGTTCACATAGTAGGCTATTGTATGAGCCTTCAGAAATCGCAACAAGTAAAGCGGTCATACCGAACCAGTTGTCTACCCAACCAACCATAGGCTCATCAATCATTGCCGTTACACATGATGGTCTAACAATGACTGCGGGTACGTGAGCACGGTTTTCTGCTACTACTGCTTCAGCTATCGCTTTCGTAAACGTGTACGTGTTCGGTCGATTACATAAAGATTTCATGTCCGGCCTCTTATCAAAACCGAATTTCTCCGCAAGTTGATAAGCTTCTGCGAGAGGCCTGGGAGAGGGGTAGACTTTTTCATCGATGATAGGTCTATCGATATTCGAGAAGGCTGTTGATACATGCACGAATGATTCAATTTTCTTCATACGTTTTGATAATTCCAGAACTTTTCTAGTACCCTCGACATTGATGTTCAACGCCACTTTTAAAGGTTCATCAAATCTTACTGTTGCAGCTGAATGGATCACCACTGAGACCTTGTCTGTTAACGTTTTCTCGTCTTCAGGTTTAATGCCAAGATTAGGTAAAGTTATGTCACCCGAAATTGGAATTATTTTCTTTAATGCCTCCGGTTTTTCCTTCTTTAATCTCGTAAAGATTATATGATGACTAAGTTGGTTTACTCTTTCTTCGATGCTTGATCCTTTTTTCTCTCTTGCTAAAATGTATATTTTTTCTATATCCTTGCAACTATATAAAAGTTTTTCTATGAAAGCCTTTCCAAGAAATCCAGTTCCACCGGTGATGAAAACAGACTTTCCGGCGTAAAATTCTGCGACCGATTGGTATTTGTTTTCATTTTTCACAGTTTTAACATCGACAGACGTTTGGCCCTCTCTTTTGGGAGCAGACGTAGAGGAGTAAGATAATGCTGTCTGCAATTTTTCCTTTCTATTCATTAACACTCCATTGGGGTCCCGGAGTATTACAAATTGTCGAATTTTACAAAACATTTTTTCCTTTTAAATACTTTTCACTCACAATCACAATATATATATATATACCTATATATATATATATAATCACAAAATATAATATATATATATATATATCTTAACCAGCCAACCAGCTCTAAGCTAATGAGATTTTTATAAAGGCTTACAATTATTTAATATTATGTACATACATGAAACGAATAAAATGTACCATATAATAGTGATGACGTATTAGTACATAAAACTGTCTGAAACTCGTACTTTGATGTTAATTAGG

>SinsFAR7

GTTTATTTTCATTAGTTGAAAGACGTGTTCAATTCAATTGTTAGATACTCGTATTCGATATTTGTTCATAACACGGTGCTGCTATGTCTTCCGAAGGTTACTCAGAAAGCGAACTGGACGCCATGCCGGACCGGATAGCTGGTACCTTCTCAGGGCTGAAGCTTCTCATCACCGGAGGGACGGGGTTTATGGGCAAGGTGCTCGTGGAAAAACTCTTGAGAAAATGTCCCGATATCGATCACATCAATCTTCTAGTTCGAGCGAAGAAAGGAAAGAATCCCAAACAGAGATTGGAAGAAATGTTTAACGGTGAATTATTCGAGAAACTTCGCGAAATGCGCGGAGGCGTAGAGCCTTTGTTAGCGAAGGTGTCCATAGTTTCCGGTGACGTGATGGAACCTGATCTAGCCATCAGCAATGATGACAGACAGAGAATAATCGATGAAGTGGATATTATCATACATGCTGCAGCTACTATCAGATTTGACGAGGAATTAAAGAAAGCTGTGCTCCTGAACGTTCGTGGCACAAAACTTATGGTGGAATTGGCCAAAAAGTGCAAGAAACTAAAGTTATTCATCCACATTTCGACCGCTTACTGCCATCTTCATGAGAAACTTCTAGAAGAGAAGCCGTATCCTCCTCCAGCTGATCCCCACCAAGTCATCCAGGCTGTGGAGTGGATGGACGAGGACACAGTCGCTGCTCTCACACCAAAACTTCTGAATACATTGCCCAATTCATATGCGTTCACCAAAGCGCTGGGCGAGGCGTTAGCTGTGGAGGCCATGGAACACATACCGGTCATGATTCTTAGACCTTCCATTGTGATCCCTATTTGGCAAGACCCTCTGCCCGGATGGACAGATAACATTAATGGTCCTACAGGATTACTCATTGGAGCAGGCAAAGGCGTGATTCGCACTATGTATTGTAAAAGCGATAGTTACGCGGACTTTTTACCGGTGGACGTCTTCATCAATGGCATCATGATAGCTGTATGGAATTATATTACATATGGCGACAAAGCGAGGAATGTGTTGAATTTCACATCGTCAGCGGAGATCAAAGTAACTTGGTCGGAAATGATTGATGCTGGACGCGAGATCATCATGAACAGGGTGCCTTTGAATGGAGTTGTCTGGTACCCGGGCGGTTCGATGAAACACTCCCGGCTCTACCACAACATATGCGCTCTCTTCTTCCACTGGATACCTGCTATTTTAATCGACATTCTGCTCTTTTGTCTCGGCTATAAACCTGTACTAATGCGAGTCCAACGTCGCATAAGCAAAGGTTTCGACGTATTCGAGTATTACACGAACAATCAGTGGGATTTCAAATCGGATCACGCTCAGACTGTGCGCCAGCGCCTCAATCCGAAGGAAAGGAGGGATTATAAAGTAGATGCTGTCGGACTTGACATATCGAATTATTTCGAGGACTGCATAAGGGCGGCTCGCGTATACATTCTGAAAGAATATGATGATACACTACCAGCTGCTAGAAGACATATGAAAGTCATGTGGTGGGTAGATCTCATCACTCGTTGCCTATTCTGGGGTCTTCTCATTTACTGGGCGTACGGATGGATGTCCTCTCTCTGGAGTTTTGCGACTGATTCGAGCGAAACGCTAAGCGTTACGGCGGCTATCTGACCGCCATATTAGTGTAAGCTTGGTGAATAACAAAATAGCGGATGGGAGTTTGGGGGGTATTTCTCTCTCTGTCTTTCTTTCTCTCTCTCTTTCTCTCTCTCTTGTTTTGAACTATTTAATAGCTATCCTTATCTTTTCTTTTAACTTTGGTTGTAACCGAAGAATATTTATCTTATTGGTTACAATAATCCAATAGTTTGGATAATATATGGATACATTTTAAGTGAATATAAAAGTTCCCATCATAATCCCGTTCTTGCCGCCAAAATTTTATTCAAGAAATGTATTCTAATATTATATTTAGCGTTGTTAACCGTGGTACTTAAAATAAAGTATAGTGCATAAAATACAGTATTTCACGTAAAATACTGTATATTATGTA

>SinsFAR8

AAAATGGCTACCGTATAACGTGATTGCAAATTGATTTTTTTTACAAGAAAAAACGCCGTTCAAACCCTTCTAACACATTGAATACGCGACAAATATTATCTAATCAATATTTATTTATAAATTTAAACTAAAGCGTTATGATTGAAAATAAATTCAGTTAAAAACCTAACCTGTATCTGTGCCGATGTTAAATCTTTTTTTTTTTATTCCAAATTCGTTTCGATTACAGTTTCATTTTTATTAACAAATGAAGTTGAAGTTGTGTTCGTTTTTTGAATTTGATTGTGGATTGAAATACAACAGCCGACACTGACATATGAAATTCAGCAGCCATGTTGAATAAAACGCACGCGGTTAAACTCAACTGATACAAAAAGACCTGAAATCTGTACAAGAACAAAAAAAAAAACAAAGCAATTTTCCAACGAAAACCTGAATTCAAAAATCAATTATTATTTTTTTTTGGGGGAGAAAATCACGCACATTTGTGATCGTGTTCATTAATTAAGTAAAAGAAACATTGATGCATTCAGCAAACACGTTTGCTAGAAGTTTCTGAATTCGGTTTCGGCGGGAAAGTTCGCTTCAGATCCCGAGATGGCGTCATGTCTCGCTGGCGGCCATTTTGGCGCCGGACAGGAATATACGCCGGTGGCTGAATTTTACGCAGACAAATCGGTTTTTGTTACTGGTGGCACTGGATTTATGGGGAAGGTGCTTGTCGAGAAACTACTGAGGAGTTGTCCCAAAATAAAAAACATCTATCTATTGATGAGACCGAAGAGAGGACAAGATGTGGCGTCTCGTCTCCACGAACTCACACAATCACCTCTGTTCGAGACGTTGCGGAAAGAAAGGCCTCAAGAACTGAACAAAATAGTTCCAATAGTTGGCGACATTACTGAACCACAGCTGGGTATTAGTCCGGCAGATCAGGCCATGCTCTGTCAGAAGATATCTGTCGTGTTCCATTCGGCTGCGACCGTGAAGTTCGACGAGAAGCTGAAGTTGTCGGTCACCATCAACATGCTGGGCACTCAGCAGCTCGTGCAGCTTTGCCATCGCATGATGGGATTGGAGGCACTGGTGCACGTGTCGACGGCGTATTGCAATTGCGAAAGGGAACGCGTGGAGGAGACGGTGTACGCGCCGCCGGCGCACCCCGAGCACGTGGTCACCCTGGTGCAGACCTTGCCTGACGACTTGGTCGACAGGATCACTCCCGATTTAGTTGGTGACAGGCCTAATACTTATACATTCACAAAGGCCCTAGCGGAGGATATGCTCATAAAGGAGTGCGGCAATCTACCGGTCGCGATTGTGCGGCCATCTATCGTGCTCTCGTCACTTCGAGAGCCGGTCAAAGGATGGGTGGACAATTGGAACGGCCCCAACGGCATCATAGCCGCCGTGGGCAAGGGAATATTCCGCACCATGCTGGGTACCGGCACGCGCATCGCGGACCTGGTGCCCGTAGACACGGTCATCAATCTAATGATCGTGTGCGCGTGGAGAACGCATTTGAGAAGGGGCGATGGTGTAGTTGTATACAATTGCTGTACCGGTCAACAAAACCCCATCACGTGGCAGCGTTTCGTCAGAACCAGCTTTAAATACATGCGAAAACATCCATTTAGTGAAGTGGTGTGGTACCCCGGCGGCGATATTACTAGCAATCGTCTCAAACACAGCACGTTGACGCTGCTGGAGCACCGCGTGCCGGCCGTCGTCATGGATATGGTGGCGCGGGCCACGGGGAATAAACCTATGATGATGAGAGTTCAAAACAAGTTAGAGAAAGCCGCGGCCTGCTTAGAATACTTCACCACCCGCCAATGGGCGTTCTCGGACGACAACGTGCAAGCGTTATGCGCCGCCCTGTCGCCGGAGGACCGCGCCATCTTCGACTTCGACGTTAGCAACATCGACTGGGACGCGTACATCGAGTCCTACGTGCTTGGCATCAGGAGGTTCCTGTTCAAAGAGAGTCCAGAAACGCTGCCCAAGTCCAGGAAGTTGCTTCGTAGGTTACACATAGTTCACGTTCTAACTCAACTGGTGGCCGTGTTCTTCCTATGGAGATTTTTATTCTCCCGTTCCACTGCTCTTCGTAATATATGGCGGCGCATTGTAGAGTTATTAACGGGTGCAGCGCGCCTATTAGCCATAGCCTGATGATAGAAAAAAAAAAGAAAACAAACCTCGTTGACAACGCCCCGTTGAACAGATTAGCAACGCTTCTGTATTCGCTATTATAAAGAACCTAATTTTGACATTTATTTCTTTCGTTGTGACATGTGTTTCAAAATGGCGGTCTCCTCATACGAGACTCGATTTGTGTACGCGTTGGAAGTTAAGAATCGAACAAAACGTTTTTTTTTTCATCGATGGTTAGTTAATGTATAGGTGAAAATTGACTAAATACAAACGTATAAGTCTCAAAGTTGATTTTGAAACTTAACGCGTACACAATCTAAAATTGATATTGACACCAATCGCGTTTATTGTTAAGTAACACAACGGAAAATAAATTAAACTTTAATATTAAAGAATACATAATACGTTTAGGAAATATTGTTTTTTCCTCTTCAATAGTCCAAGCCATTTGTAAGGGGCTGTTAAATATTTTTGCTTCCTTCAAAAAAAAAAAAAGAAATGGCGTCGTGCGTTCTTAAAAATCGGTCGGCCATTTTATAATGTCCAGTTGTCAAAATAAGGTTATTTTTAATGGCGCACGATTTCATTTTTTTTTTCCACTTCCCAAGTGCTAGGCTTTTAATATAGTTCCGTTACTGTTAAAATACCTTATAAATTATGTAATGTGTAATAAATAAATGGTAATTCCAAAATAATTTTGTACCAATATTACTACAGCTCAATAAATATTAACCGATATATAGGAAAAAAAAA

>SinsFAR9

AAATTTTATTTATAAAAAAAAAAATTAAAAATTTTAAAAAATTAAACTAATCACAGACCAGAGTAATCTAAAGAAAATTATAAATAAAATCGTAGAACAGATGTAGTGAATTAATTTTAATTTAAAATGCTTTCTAAGACCAGCCGGTACTGTCTCTATGTCGTCTTTGAGCAAATAAACTCTGACTCCTCTGACATAATTGTAAAAGTAGTCACTCCAGTCGACGCATGCTATGTCGAAGTTGAATATTTGTTTGTCTGCGTCGCATAAATCTTTGTACAGATTCGTTGTGTTGCTATTCGTGAATTTCCATTCCCTTAGTGCGAAGTAGGCCATCACTTCGGAGAATTTGTCGATTTTCGTGTAAGCTTTCCTTAACATAGGTTTCTTGCCAATTAACACGGCGACTCCGTCAACCAAGTAAGCTGGTATCCAATGCAGCAGGAAACAGTATAAGCTGTAAACGAACTTGGAGGGCGTCAGATAGAACAGGTAATACCAAACGGCCTGCATCGTGGGCACTTCGAAGCCGTACGTTTCATTGAACTTCATGAATCTCTCCCAAGTCAGAGGTCTTTGCTCCGAGGAAACGTAATTATAAACCGGCGGAGGCATGTCCGGTGGGGGTGCGTCCTCGTAATTCCCCTGATAGTCTCTAGCTGTCTTCCAGGCTGCGGCTATGCAGCCATTCACCACCATGTCTCCTGGCACCAGGTCTGCGATGGCTTTGGGGCTGCAGTGAAGCACGTGTAGGAGGCCCACGGCGGCGCCTACAACGACGCCTGTTGGTCCGTATACATTGTCGATCCAACCCGCGAAAGGTTCTTCGGCAGTGGCGATCACTATAGCGGGTCTAAACACAGCTACAGGTAGTCCCTTTGCATAATTGCGGACCAAGTTCTCCGCAGCAGATTTTGTGAAAGCATATGTATTTGGAAAGTCTCCGATGAGTCCGGGTGTAATATTGTTCAGAATTTTCTCATCCAAAGTTTCAACTAAGTCGATTAATTTTTCTCCGGGCACAGCGCTATCATAGAACTTCTCCTCGATAACATTCAGCGTACAATTACTATAAGCAGTCGATATATGAACCAAAGCTTTCAATTTAGTGCAAGCTCGTGCCAACAGAAGCATTTCTCTTGTTCCGCGCACGTTGATCTCTACCGCCGTTTTTAAAGCTTCGTCGAATCTTACGGTTGCTGCTCCGTGGAAGATGATTTCCACTTCGTTGATAACTCTCTTTCTGTCTTCGCCGCTGATACCGAGACCAATTTGTCCGACGTCACCTTCTATGACGCCGATTTTCTTCAAAAAATCCGGTCGTTCTTTCTTTAATTTACTGTATAACACATCATCGAACTGTTCTTGCAATCTTTTTGCCGTATCTTTATTTTTCTTTGGTCTTGCCAGAAGATAAACTTTCTTTAGGTCTGGACATGATCTCAATAATTTTTCCACAATCAATTTTCCTAGGAATCCGGTGCCTCCGGTAAGAAGCACCGTCGTGCCAGTATAGAACTTCTGTATTTGCGATTCACCGATTATCTGCTTTTCTTCGTCCAAATCTGTATACGGGATGTCGGTTTCCTGTCTCATGATCTTCACGTCACAGTCCACCGTGGTTCCGTCTACCGTCATTTCGTTCCGTATTCCGAAGTTTTACCAACGAAAAAATAATTAACAAAATTGGAATCGAATCAACACTCCCGGGGCATTAATTTGGAAAGTCACTATATTTATTTATCGCGAACAAGGACTAGACTTCTCTTTCGCCTAAGAACGTGTTAATAGCACGAGTGAACAAGAACCACTGAGCAATA

>SinsFAR10

TTATAACTCAAAATTTTATTAAAATAAATGCTTACATAATAACCATAAGTACAACATACAATATTCACCAAAATAAAAAGCAATTTTTGCTTAATACTAAAATTCTTTTATTTGTTCAGGCTTATGCGAGGAACTGAAAATCGACGAAGAATATTTGAAAAATATCCACATCATTAAACCTATGAAAAGAACACGGAATATCAGGTCCACTGCTTTCATTATTTTCATATGTCTCTTTGCTGCGGGTATAGTGTCATCGGGTTCCTTTAGGATGTATACTCTAGCAGCGCGTACGCAGTCCTCGAAGTAATCTGATATGTTCACACCGTCAGCATCGACTTTATAGTCTTCCTTTTCTCTTGTGTTCAGGCGCGTACGCACCATTTGGCCTATTTCTGATTTGAAATCCCATTGATTGTTTGTGTAGTATTCGAATACATCGAAACCTTTGCTTATGCGGCGATGGACGCGCAACAACACAGGTTCATAACGAAGTATGATAAGAAGTGTGTCGATCAAAATGGCGGGAATCCAATGGAACAAAACGGCGCATATGTTGTGCGCAAGACGAGAATGCTTCATTGAACCACCTGGATACCATACAACTCCATTAAGAGGCACTCTGTTAAGTATGATCTCTCTACCCGTATCAATCAGCTCCGACCACGTCACCTTGACCTCGGCTGAGCTGGTGAAGTTGAAGATCGTAGATTTCTTGTCGCCATAACTTAGGTAATTCCAAACGACCACCATAATACCATTGATGAATACGTCTACGGGTAGATAGTCGGCGTAGCTGTCGCTGCGACAATACATTGTGCGAAGTACCCCTTTGCCAGCGCCTATTAGCAGACCAGTGGGCCCATTTATGTTATCCGTCCATCCTGGCAACGGTTCCCGCCATATTGGGATTACAATTGAAGGTCTGAGGATCATGACGGGGATGTGCTCCATGGCTTCCACTGCTAGAGCCTCGCCCAGGGCTTTCGTGAATGCATATGAATTAGGTAGCTTGTTCAATAGTTTAGGCGCGATGCTTTCAACAGCTGCGTCGTCCAGGAGCTCAACTGTGGCAAGGACGCGGTGAGGGTCTGCAGGTGGTGGATACGCCTTCTCTTCCAGAAGCTTTTCGTGCAGGTGACAGTATGCCGTTGATATGTGGACGACCAGCTTCAATTTCTTGCATTGCTTGGCCAAATTCAGTATCAGTTTCGTTCCTCGCACATTCAGTAATACGGCTTTCTTCAAAACTTCATCGAATCTGATAGTGGCAGCAGCATGAACTATGATATTCACGTTGTCCATGATATACTGGCGACTGCTGGGCGTCAGGCCAAGGTCTGGCTCAATCACATCACCTTCCACTGGCTTTATTTTCTCCAGTAGACGGGATACCCCTCCTCGCATTGTCGCCACTTTTGCGAACAGCGGTCCGTTGAAAATATCTTCAAGGCGCTCTCTTGGACTCTTGCCTTTCTTGTTTCTCACTAAGAGTATTATCTCTTTTATTCCAGGGCATTTTCTTAGTAGTTTTTCGACGAGCACTTTCCCCATAAAGCCGGTGCCTCCAGTGATCATCACCGTTTGTCCTGTGAAAGTTTCCGCGATGCGATCTGGTAGTTCGAAATCCATCTCTGAAGTCTTTTCCGTCGACATTTTGAAAGTACTTCTTTATAAAAAGTACTGTCAATGAAAATTACACTTATATTGTCACGGTAACGAACACAGAAACAAAAGCAGTTATCAAAAGTCACGGACTACTATCGTGGTAGTTTTGTGTGCAGCTACTTTTCTATAACCCCCGCCA

>SinsFAR11

GTTTTATTATACATTATCGGATACTAAGATCGAATACTAGCCATTCTATATTGTGACTCCGCAGAGTTGAGTCGTGTGCAATAATTTACTTTATTTTTTCGAAATTAATAAAGTGACAGTTGAATTATTAATGCATTAAAGAGTTTTATTTCAGTTTTTAATTTGAAAGTGTGTTTCGAGTTACGACGGCTATTTAGGGTTAATTTCGATTAGAAATTAATGAAAATGGCGCTATCTATGAGCGTACCAGAGTACTACGCCGGGAAGACAATCTTCATAACCGGCTCGACTGGTTTCATGGGGAAAGTGTTGGTGGAGAAGCTCCTCAGATGCTGTCCAGATCTGAGCAAGATTTATATGCTGATGAGACCGAAGAAAGGGCAGAGTTGCAAGGAGAGATTAAACGATTTCGTCAATTGTCGGGTGTTCGGTCGTATCCAGAAAGAGTATCCCCATACGTTGAACAAGTTGAAGCTCATTCAAGGCGACATATTGGCTGAAGATATGGGACTCTCCAATGATGACCGCGAGGAACTGCAGAGGGAAGTTCAGATCGTGTTCCATTGTGCTGCTTGTGTGAGGTTTGACATGGGTCTGCGTGACGCGTTAAACCTGAACACAGTGGGCACAAAAAGAGTGCTCAAACTGGTCGAAGACATGAAGAATATAGAGGTGTTTCTCCACGTGTCGACAGCGTACTGCCGCTGCGAGCTGCAACAGCTGGAGGAGAAGCTGTACCCGTCCAAGCACCGCCCCCAGCACATAATGGAATGCTGCAGCTGGATGGATGATGATCTGCTCACACATATGGAGCCTAAATTGATCGAACCGCAGCCGAATACTTATGCGTACACCAAGTCTTTGACTGAAGACTTGGTGTCACAACACGAGGGCAAATTCCCGATAGCGATCGCGCGGCCATCTATCGTAACGGCGGCTCACAAAGAACCAATGCCAGGTTGGGTGGATAACCTCAACGGGCCAACGGGATTGTTGGTCGGTGCTGGAAAAGGTGTGATACGAACAATGCTTTGCAATGAGAATCTCAAAGCTGACGTGGTTCCTGTAGACTTGGCAGTGAATGGCTGCATACTGTTGGCTTACTCGACAGGAATGGAAAAGCCAAAAGAGATTCAAGTGTGCAATCTGACGGAGTCTGGACAGAATCCCATCACTTGGGGCAAAGCACTTGATATGGGTCGCGTCCACGTGCAAGAATTCCCATTCTCCACCTGTCTCTGGTACCCTGGCGGCTCCCCCAAGAGCTCCAGAGTGCTGCATCAGATCGCTCTAATCTTCACGCACTTGCTGCCTGCTTACTTCATAGATCTTCTACTCTATTTGCTGGGGAAGAAAACTTTTATGGTGAAAGTGCAAAAACGCATCAGCTACGGTCTAGAAGTGCTGCAGTATTACACAACAAAGGAGTGGTACTTCAAGAACGACAATTATCTTGCGCTACGCAAGAAAATAACGAAAGAAGATGACGAAATCTTTTACACTGATATTAAGGTGGTGGATTGGAGCATGTACATCAGGGACTACATAAAGGGCGCACGCGAGTACTGCTGCAAGGAAGATCCTTCCACGCTGCCGCAAGCCAGGAAGCTGCATACACTATTGTACTACGTAGACAGAGCGGTCCAGTTTATCATCTATTCGCTATGCGCATATTTTCTATATTTCTATATTAAGTTATTCATTAGTTTCCTCAGCTAATTAATATATGTATGACTATGTGTAACATAAATATATAATCTGTATTGTACGTCAATTGGTAATATACCATATTCTTAATAGGTATGTCGATATGTTTATGTAAGATGTTGAATTTAAGGGCACCGATCGGCCAATAGATGGCGTTCATTTTTGAAA

>SinsFAR12

TTTTGTTTTTTATGTACTTAGGGCTAAGGAAAGGAAGTCAAAAAAAATGTACTGTAATTATATAAAACAATGGATTTTTTGGTTATTAACTTTAGAAGACATACATAATGGTCTGTGATACATAATACATACATAATTATATTCTTTCTGACTTCTACTGAGGTAATTAAAATCGAAATAGATAGTTTATCATTGAAATGTCATAAAACTATGTTTTGTGTTGCTATATGCATAAATATTCACGTAAGGACGGAATATAGTATATTTTAAATGTCAACGCCATCTATTGACACATCGGTGCACATCTCGTCGATCGATCATCGTCGAACTTGAAGCTGTTGTTCCCAGTTTTCATCGTGTTTAAAACATGATGAAAGTCTTTTCCGAAAGGACCGAATGCTGTTGGACTTGAGAAGGCGTTGTAAAGCGTCACCGAACGATTGGGGCGAGCGCTTATGGCGCTCAAGACGAGATTTTAGTGCTACTGGCCTAAGGGTACCACCTCGGAAAGGCCCGGGGTCGGCTTAAATCTCCCGCGCACCACACAGGGCTCGCGTGGTGGAGTATGAGCGTATGTTCTCATGCCCAGCTGTGAGTGAATAATAAGTATACCTAAAATATTTATGTACAAAATGGAACAGATTGGAAAGTTAAATCTTTAGTTTCTGTAGCGCTGAGGGCGTGAGGCGAGCGAACAGCGCGCTGAACAGGGACAGCAACAGCCTCAGGGTTCCGTAGACGAACTTGCGCGCGAACTTGTTCTGCAGCACCACACGTAGCAATAATATACTCGAGATCAATTTCACGGCATTGTGTAGAATGTACATTCTGTGGACGTGACGCTTTGCAGCTTCCAGATCTTGATCTTTCTCTTTGAGTAAATATTTTCTGGTCCCTCGGACGAAGTTGTAGCAATGTTCGTCCCACTCAATAGTGTTGGGATCCAGGTTGTATATAGCGGCGTCCTGCGGCGAGAGGCGCGCGCGCAGCTTGCGCACGCGCTCGTTCTTGAACTGCCACTCGCGCAGAGAGAAGAACGTCAACACGTCGTTCATGGCGCTCAACTTCTGGCTCGCTATTATCAAACTCAATCTTTTCTTAATGCGCAGGAGTCGGGCGACGTACTCGGCGAGGTGCAGCGGGGCAGTTTGCAGCAGGAATTCGTACGTTTTCAACACATATCTATTCTCAAATGTAGTCCCCGAAGGGTACCACAGCGGAGCGTCCATGGGGTACTCCCTCATCGCCCGCCAGAGCGTCGTCTCGAAGCGATGCCACGTTATGGGATTCTCGCCCGTAGAGCAGTTGTATACGACAGCTTCTAATGGTCTGTCAGTAGCAATTTCCCAGGCGACGGCAATGAGCGTGTCTATAGCGATGTCGACCGGCAGCAGGTCAGCTCGAGCGGACTCGCGGCAGCAGAACACATGGAGCATTCCTTTGCCAGCTCCGACGACCACCCCGCTGGGACCGTTCAGATTTTCCACCCAGCCGGGGAACGGGTAGCGAAGTGATGACACCACTATGGTAGGCCTGAAGATAGCGACAGGATATCCGCGTCCAGGATACCGATTCACTGCAGACTCGGCCATTGCCTTTGTATACGTGTATGTATTAGGTTTAGGTGCTATGTACTTAGGTGTGATGAGTTTTAATAGATCGGCGGGGGCAGCGTCCGCCAGTGCCAGGAGCTGCGCCATGTCTGTCGGTGCTGGGTAGACGCGTTCTTCGACAACGTCCTGGTCCGCGTTGCTGTATGCAGTCGACACGTGGACAAACGCCTCAATGTGCGGTAGTTTGTCGCACATTTCCAAGAGTGCGACAACCGAACGCAGGTTCATGTCCACAGCTTTCTTCAGCTCCTCGTCGAACTTGAGGGTCGCCGCGGCATGGAACACCACTGACACTTCCTGCAACAGCTGCAAAGCACCAGAGTTCAGCCCGAGGCCAGGCTGCTCCACGTCGCCCGCAATCGTTACTATCTTGTCCAGCTGTTTTGGATTATTCTGACGTATAACATCGAAGACCTGCGACTGTTTGAGTTGCAGCAGCCGGACTTCAGGCGAGGAGTTCTTCTTCGGTCGCATCAGCAAGAACAGGCGGCCGACGTCGGGGCATGTCGCTAGTATACGCTCCACTAGCACCTTGCCCATAAACCCAGTGCCTCCCGTGATGAGTATGGACCGTCCGGCGTAGAACTGTGGGATTTGCGGCCCGGAGCTCATTACTCTTGCTACCATTGTGGTTGCTTTAAAACTTTTACATTGTTTTATTAATTTTTGCCCATACAAATTCCATCTCAATACTTAATTTTATACCCATGATTTTACATGTATAGTGCTTATATACTCGCCTAAGCATTTCTACAATACCTACCTACGTCATATTGAATACCTGAACGAAAATATGTACATATATATTGTATACAATTTAGTTAGTGGGAAAATT

>SinsFAR13

GGGGCCCCAAAAAAAAAGGGAAAGGGAACCCCCCCCCCCCCCCCTCCGACTCGTGGCAAATATTTGCCCTTGAAACAGATATTTGCACGGTGTGGGAATCGAGCCCGAGACCTCTGTGTTAATATATAGCCGCGCCACTGCGCCGCCGAGGGGTCGTCAAATATGAATAGAGACATTTTGTCTGCGTGTGTCATTGGCTCACGTCCTAATGACAGGTTTAAAGCATAAAAAAAATTACATTTTAAATATTTTTTTCATATATCAAGTTGTATAATGAAATTTTAACTATCGCGTTTATGTGCATTTCAACGTTTATATCTTAAAAAATATTAATTTAATAAAAGACTACTTTGTAAGTATACACATCACTCAAGTGATGCATTAAGCATCGATATGCTAATATTGATCGATTGCGTTGATTTCAAATATAATATGTTGTTACATTAACATTTATATATAACATATAACAATACAGTTTTACTAAATGTCTGGTGAAGTATTAATTTTTTTCGACTATAAGTCAACTTTACAGGACATTGACGGAGTCATTATAATTAATGGATTGAACTTGGCTCGATTTCAATCCATTAATTATAATGATTCTGTCAACGTCCTGTAAAGTTGATAACCACACATGGATCTGTTTTATATGATTATCAACTTTACAGATTTTACTTGATTGAAAACAGCGGAATTTAAGTCGAAATAAATAAATGGTTTACTACAATCATATTTCGCGAAATGATGTTTTATCAATATAGGTAAAAAAACCAACGCCATCTCATCTATTAACATATCGGTGCTTAAATAATTATGTTTAATTAAAAAAAGCATTAGGCGCCATACTGGAGTCCCAATCATCCAGTTTAAAATATTTTTTAAAGAAGATATATAACATTCGTTTTTGCTTCTTGTAAAATTGCATTTTTGTAGATGAGTTATTTATACAAAATCATTGATATTTTTGCATTTATAAATAAATACTAATAATTTACATATAAAGCCATCAATTAGGAACTAGTTATAAAGAAGCGTTTTGTGCGCGCGCCCAAGAGGCTGCATTTCGGGATCTCTTACAAAATTGTATGAATATCTATCTGTGCAATGAATTTTATTATCAACCGCCTAGCGTCCGTGGCTCGGACGCGCGCACGATACGCTTGTTATAACTTCTCCTTAACAGTTGATTGTTACTGCATTTTCCATTTGGGGTCTTAAAGCCATAGTATGAGATTATATTTTCTTTAACTCTGCAGAACGCGCAACCCCTGAAGTCTTTCTTCATGTACTTTTCAGCAAAAAAAAAAAAAACATTTTTATACAATAATTGACATTTTCTCGCTAAAACGCTCCTAATTGTCATGGCGGCTTGTGTGACGTCACATGTTAATACATTCATACAAAACGCTTCGTAAACTTGTAAGTACGGGCGTGGTACACGCCGTGACGTCACGCCCTCTCAATTAGCGTTTCGACATCACGTGACTTTAATTCATATTTTATGTTTTTATTTTAATAAAATTAATATATTTTTAACTTCTAATTTATAAATCAAGTTGATTTTCTGCATATTAGCAACTAAATCTATAATTTGAAATCAAAATTTTAATTTTATCTTTTTACTGGAAACTAGCCTATTATTTTAATTATTTTTAATATTTTACGCATTATTGGTACTTTATCGTCGATATGATGTATTTGAGTAATGAATAAAGACACATTTTGAATATATCGCACACTCAAACATAAAGCGACTGAAATTTAAAAACATATTGTAATAAGTTCTTTAGCCGCAGGTTGCGACGGCATGCTGGTCGGAACAAATTATGCGTGACACGAAATGCGTTACTTAAACTCTGTCTCTCTCCCTCTACACTCACTGCTTGTTGCCTCTTGTGTAGTTTTCATAAAGAAATAATTAATCTAACCGCCATCTAGTCACTTTTTTTATGAACAACGCCATAAGATTGTCAAGTAAAAAAAAGTTTAGTTGTAACCTGCCTTATTGATATTGCACGTAGTTTAGTGTATGACCACTAGATGGCGTATATACGATATACGAACCACATTAAGTTACTTTATAATGACAACAGATGGCACTAATTGTAATATCAAAATATCTTCACCAACATTTATAATTAATATTTGTCAAATAGCGTAAGAAACTTCGTTCATAGCGGGTGTCCCGCGACACCACACGGCTTGTTTATTATTATAGATACCAACATTAACAAATGTAATAAAAATTTACATCTGAAGTCTAATTAACGCAGTGACTACATTTTGACGCAGCCGTTTATATGGCTGCGTATAGAGATATCAATATATTGGGAATATCACAACGATAAAATTACAAGATAAATATTGCTATGATCATTACATGAAATAATGTTCCCTGATACTTTCTGCTTTAGTGTATGTACGAGTACTATTTCTGTGTTTTGTTTTTGTATCCATTTTGATTTATCTTCAAAATATACGTAAGGAATACTTATTTCTTGTTTTATTGTTATGTAAAAATTACTTAAACTCAAACTCAAATTATTTAAACTGAAACTCTTGAGTTTTTGCACTCGCGCTTGAGATAAGAGTCGCTTGAGATACGAAATTATCTTTAATATCTCAACTCCCCTTTGTGCGTTTTAATATTTTTATTTTAACTTATACGGAATATAAATGAAATCTGTTCCTGAAATAAAGTCTAAAGAAAATTATGTATTGACATAATATGCATCTACATCGTAAGTTGAAGGTTCATCGACTTGAGACAAACATTATTGTATAGAAAATTTGACGGTTTAAAAATTGTCCGTTTATAATTTTTTATAGATAACGTCGTATTTTAAAAAGTTAAATAAATAAATGTACAAATATGTGCGAATTCAAAAATTAGGGACCGTACGCGTTTGTATGAACTGAAATGTTCAAGGTCCAAGGTCAAAACCCCATGCTCAGGGAAGCGCCTTAAGTTTTGATAGTCTTGAATTAATTACAAACATGTCAAACTTATTTATATCGCCTCTTTTTTTTATCAGGGGATAAAAACAATGTCTATAGGAAACTATATAATATATATATAACAGGTGTTATCCATACACTTTGAGCCATGCTCAGTCCAGTCAGACGAGCGAATGTATACCACACGAGAGCCAACATTAGCGTTTGCCAAATTATATGGACGACCAGGAGAATGGAGTCCTTGCTTCTAGCGGCGGCTAACGTTTTCTCCTTTTCCTTGTTGAGATATCTACGGACTCCTAAATGTAAGTTCACGAAGTAATCCTTCCAATTCAACGTAGATATGTCGATATAAAACAACTGTTTGTCTATCTTGTTAAGTTTAGCGGCCAACTCTATCGTATTCGTGTTGTGGAAACGCCATTCGTTGAATATGAACTTTTCCAAAAGATTCAAAGACGACCAAACATTTTTGTGAAGACGGAACAGAATAGGCCTTCCGCCGGTAATCCTTAATAATTGATCGAGTAGAAATCCAGGGAAGAAGTGGACAAATATGGCCGATAGGCGAAACAGCCACAGCGACGGCACGAATTTTAGATGTGGGTACCACACAGCACTTTTTAAGGGGTATTTATGTAAAATTTCATTGATTTCAGATTCTATCAACTCCCATTTGAACGGTCTCTGCGTAGAAGATGAACAGTGGTAAACTGTCAGACCAGATTTCTCCTTGGCAGCGTACCATCCAGCGACTAATAGTTCGTTCACGACCACATCGACTGGTATGTAATCCGCGATATTTTCTTTAGCCAATGGCAGACGACGGACCACACCTTTAGACGCACCCATGAGAAAACCTTGGGGTCCAACTTTGGAACACGTCCATCCTGGTACTGGCTCTTTCCATGCAGCCACAATCATAGTGGGTCGGACGATGGTACATGGGAACAAGTTAGCACAATTAGCGACTTCATGTTCGGCCAAGTGTTTCGTGAATGTATACGTGTTTGGATGGTCTTTCAGTATCTTTTTCTCAATATTGTCCAAGCCTTCATCAGATAAACTATTGACAAGGGAAATGACTCTTTCCACGTCTTCCGGGGCCTCGTAAACTTTCTCGTGAGCTTCGTTCAAGTATGAATTCACGTAAGCCGACGATACGTGAATTAATACCTTTAAATTCTTGATCTGTTTACAAAGCTCAGTAATACGCCTCGTTCCGAGAACGTTAATTTTCACCGTGGGCCGCAAGTTTTCTTGGAAGTCCAGCGTCGCAGCCGAATGTATGACCACATTGATATTCTCAATTAGTACTTGTCTATCCTGTGCACTAAGACCTAGATTCTCTTCGCCGACGTCGCCCGATACGGGGATTATTTTTTTGAAAATATCCACAGATTGTGTCTCTAGGAGTTTTTCGAATACAGGATGTACTTGTATTTCCTCAACTCTCTGCGATATCTCTTTGCCTTTCTTAGGTCTCATGAGCAAATACACATTGCCTACATTCGGTATTGTGCGCAATATCTTCTCTATAAGACATAGGCCCACGAAACCGGTGCCGCCGGTCATGAAGAGATTCTTGCCGGTGTAAAATGCACGCACTTGCGACTCGTCAGCCATTTTTTTTATGGAGATTCAATTCTGAGTTGTGGGAAAAAAATCTTGAGTAAATAAAACACGTTAAAACACTGTTAAACAAAAATAATGAAGATTTCCAACAAATACATTTAATAACAACAACACACGAACATCCCGCACGGAGGCGTGG

**AD:**

>SinsAD1

TTTGTACCGCGTCGCGTCGGAAAGGACGGCCGCCCGCGCCGCTTGGTGCCATTGTGGTTTTATTCGCACAACACTAGACATCTTAAATTGTTTAATTTCATTTGTACTGGTTTTTATGTGAAATAAATTTTAAAATTAATTACCAAATAATGTCCGTACCGGAGTACTTTTTGTTGGAAGATGGGGATCGCCTACCTCGAATTGGTTTTGGCACTTGGCAGGCATCCGACGAAGTTCTGGAAAAGGCAGTGGATACTGCACTGGCGTGTGGATACAGACATTTCGACAGTGCGCAAGCTTATGAAAACGAGGCCGCCTTAGGCCGCGCGCTCCAGCGCTGGATAGGAGGCGATGCGGCTAAAAGGAAAGAGCTATTCGTTGTTACAAAACTGCCTCCTGGAGGTAATCATCCAGAACTAGTTGAAGAATATTTCGAGACGTCCCTAAGAGATTTAGGTCTGGATTACTTAGATCTCTATCTGATACATGTGCCGTTCGCCTTCGAACACATTCCTGGCGATCTACATCCGAAAAACTCTGATGGCACTATGAAGATGGATACCAGTACAGACCTCGTTGCTGTTTGGAAGGCCGTGCTAAAATTAAAGGAATCGGGCCGTGTGCGACACGTTGGAGTGTCGAATATGAATGAAGAACAAATTTCTCGTCTCTGCAAAGTGTCAAAACCGGAGTGTCTTCAGATAGAAGTGCACGCGTTATGTCAGCAGGTCCCATTGATCGCCACAGCGAACAAATTTAACATACCGGTAGTTGCATATTCGCCTTTGGGTTCCAAGGCTCTCGCTGACGCTTTAGCTGCTAAAACTGGACGCGAATATCCTGATTTGTTGCAACTGCCCACCGTGCAGCGTATCGCCGAGGCGCACGGACGTACTCCTGCGCAGATACTACTTCGACAACTATTGCAACGCGGTGTCGCTGCCATTCCGAAAAGCACGAATCCTGAACGCATCAAGCAGAATATATCAGTATGGGACTTTCAGCTCAGCGATGGGGAGATGGCTGAACTAGCTGCACTCGATCGCGGGGAACGCGGACGCATCTGTGACTTTTCTTTCTTCATGGGAATTGAGAAACATCCCGAGTTCCCTTTTAAAAAATAAACATTGTTTCATTATTAAAGAACATAAAAAATAACTCTTATATTTGTGCAAAATTTATAAATAAAATATGTTCTTGAATGTGATACCAACAAAAAAA

>SinsAD2

ATATATATGTATATATATATATACTTATCGCTTCCACCGATTACTTGTTGCGAAATGACTCAGTTCAAATGTAATAAAGTAACGAGGATAAAAATAATAATTTTTTATCGTTACCGGTGGCTTAAACATATTGTCATTTTAAAACACTTATTGTAAAGGCGTGTCTGAACGGGCCATATTTTTATACTGTAATATATGGCCGCTATAGTGTTGCCAATAATGATAGCAAGAATATAATACAATATAATAGAAGTATATTTAGTACTACTTTTTTCCGCGGCTTCGTCCGCGTATAAGGGCTTGTTTACCGGAGTGAAGCGCTGAGGGCATCCTAATGCGTACACCTCCTTAGCACACCTAATAGCAAAAGTTCCATCTTAATTAATTTATCTATTGCGGTGATAAAGTCACATTATCTATTACACTATCTATTTCTTACTAACCCGTAGGAATCCTTTCATTTCCCAGGATAAAAATTATCCTAAATGTTAATCCTGAATATAATATTTTTTTTTAATTAAATTCAATGTACAGACAATCGACGGTTTTGTAGTATTATTATACACGAATAAAACTTTTGAACCTTTTAAATAATGCGACATATAAACTAAAGTACTTTAAGATTATTTCTATTAAGATTATCAATGACATTCGACTGTTCTTTAAGACAATTTCTACTTTATTTATCAATTACATTGCCTGTGCCCTTTAAGAAAACACTCCTTGGCCTAATATTTCATATGCTTTTTGGACATTATTGGTGATGTCCTCAGCGGGTCTATTTGTGGTCACCAGCCATGTGCTCCCACTTTCTCCTTGCTTGTACGCTTCTACCAAACCACGAGCTGCTGATTCTGAACTTTGAACAGGTAGTTTCGATAATGCTGCAAAGAAATCCTCATCCGTTTCCTTGTCAATTCCTCCGAATTTTGTTCTAGTTAGTAAAGCAGTATCTGTAGCTCCAAAACAAATACTCAAAACACGAACTCCTGAACGGGAGTAATGCGGTTCCTTCCCAAGGCAATTGCTGAACTGTTGAACAGCACTCTTTGTACCGCTGTAAACTGACAAACTGCTGAACTGCTTTAAAGCGACTATTGACGAAATGTTTATTATAGTACCACCTTTCCCGCCACGATCTCTACGCATTAATTCATAAGCTTTAAGCGAACTCGTTATCAGTGCTGTCACATTCACAGCGATCATCTTCAAATAGACATTCGGACTATCGTTCATAATGCCGGCATTGTTGACAACAACATCTATATATCCCGTCTCTTTTAGTACTTGCTCATAGGCTGTGTTTAAATCGTCAGTAGTTACATCGCATTTTATGAATTTGGATCTGTTCGCCCCATATTTAGCATTCATTTCGGTTTCGAAAGCTTTACCGGTATTTACGTCTATATCCAATATTGCTACATGCTTAGCTCCTTCGTTTAAAAAAAATCGAACTATATCGGCTCCAATTCCAGTCGCACCTCCGGTAACCAGCACCACTTTATCTTTCACTTCATACATTTTGACTTAAGAAATATGGAAAGTAGCAACTTATAATGCTATATACGTAATTTTATATACGTATAAGAAGTCACCTTATTTGTTGTAGCTATTAAGACAAATAATTATATGATCTAAAATATAGCTATGGTAACTATACCATACTCATATTAACGCACTTTTACAAGAGGCATGGTTCGTGCAGCGGTCAGATATGAAATAATCATTATAATACTAGCTTGCGTT

>SinsAD3

GAGCGCCTAGTGGAACGCAGCATATATAATCTCTATTACGTTTTAGTGGCAAGGTGGCAAGTGAACAATAAACATGAAAACTAAGTACAATAAGTGCCAACTTTGAGCTTGACGTTTTCATAGTTTTCTATTCTATGAATTTACCCCGGTACCCCTTGTCCCACGGTCAAGGTCGCGCGCTAAAGTAATTTCAAGGTCTTATCGTTGTTGTTGCTACGTGCTAGTTCTCGCTTTATTATCGCTGTATTTTGTTACATCACAGCACAGACTGTATTATTATTAAATATTTGTGTAAATTCAATAGATTCTCGTTTACTTTAGTTGTTAGAAGTCGGTTCGGTTGTACAGAAGCGTCTTAAGAATTTGATTAACATGTCGACGGCTGGTAAAGTGATAAATTGTCGCGCGGCTGTCGCCTGGGAAGCCGGGAAACCGTTGTCTATTGAAGAAATTGAGGTAGACCCGCCCAAAGCGGGTGAAGTACGCGTTAAAATCATCGCAACCGGTGTCTGCCATACAGACGCATACACACTATCCGGCAAGGACCCAGAGGGAGTATTCCCCGTCATACTTGGACATGAGGGTGGCGGGATCGTCGAAAGCGTCGGAGAAGGCGTTACCTCTGTCAAGCCCGGTGACCACGTCATTCCTTTGTATATCCCACAATGCAACACCTGCAAATTCTGCCTGAATCCAAAGACCAACCTTTGCCAGAAAGTCCGCGTGACTCAAGGCCAGGGCGTCATGCCCGACGGCACTAAAAGGTTCCGTTGCAAAGGAAAGGAGCTTTACCATTTCATGGGATGTTCCACATTTAGTGAGTACACAGTAGTTTTAGAAATATCTCTTTGCAAGGTCGCAGATGCTGCTCCATTGGATAAAGTATGTTTGCTGGGATGTGGTGTTCCGACAGGATATGGAGCTGCTTTGAATACGGCTAAAGTGGAACCAGGTTCCAACTGCGCCATATTTGGTCTGGGTGCAGTAGGACTAGCTGTAGCTTTGGGCTGTAAAGTGGCTGGAGCTAAACGCATTATCGGAGTGGACATCAACCCTGACAAGTTTGAGGTGGCTAAGAAATTTGGTGTGAACGAATTTGTCAACCCTAAGGAGTATGATAAGCCAATTCAGCAGGTTCTCATTGATTTAACTGATGGGGGTTTGGATTATACATTTGAGTGCATCGGTAATGTGAATACTATGCGTGCAGCTTTGGAAGCTTGTCATAAGGGCTGGGGTGTATCCGTTATCATTGGTGTGGCCGCTGCTGGTGAGGAGATCAGTACTCGTCCATTCCAGCTTGTCACTGGTCGCACATGGAAGGGAACAGCTTTTGGTGGTTACAAGAGCCGTGAGAGCGTCCCTAAACTTGTAGATGAATATCTGAGTAAGAAGCTGCTTTTGGATGAATTTGTAACGCACCATGTGCCTTTGACGGAGATCAACGAAGCTTTCCATTTGATGCACACTGGAAAATCGATTCGAGCTGTTGTTGATTTGTAAATGTCTAAGTCTGAAGTTGTATTGTTAAGCATTTATTCTTCTATGTTATAATTACAATTTGTAATTATTGTACTTTGCATTATATACAACACTTTCAAAATCGTTTTTTATGATTCAATATCCCTTTGAATGAGATAATACATAGTAATAATAATGGGATTCTATATATGAATCGGTTAATTAAGAAAGGGAACAAGTCAGCATTTTTCACAAATGGCAATTTTCACAAAATATGCTTTTTTGGGCAAGATTTACGATATAATTATGAAACGGAATATGTCGGACCATCCGTCTAAAAGTTATAAGGCACCAAGGTTTTTCAACTAGATAAGAAGAGGAACAAGTCACATGACATGTTCCCTTTCTTACTTAACCAAAATTTAGCTGGTTGGGTAACTTTTTTTTACAAGTTTTTAATTTGACATTTAAACAGCTGTTATTTTTAGGTTACTTTTTCACATTTTGTCGCGGTATAGCTGGATATTTTCTAACTTTAATTTTAGTTTCTAAATTTAAGTTTTACAAGAATACTTTAAGTTAAAATGTCGGAAAACGAATACGAGGATGTAGAAGATATAGCGAGAAAAAGGAAAGGTGTAAGAAATGAAACTAAATCGGAAAGAATCAAACGCAGTCGTATTAAAGGCCTACCATATGAAAATTACAAAGGAGCATCGGTTTCCGGTAGAAGACCTGGTGAGTCTTGTCTTTGTTCATTGAAATGTTTCGAAAAGATTGACGAAGAGTCACGAACACAAACCTTTGCTCTTTTACATGATCTCAGTACTAAAAACGAGCAGGACATACTTTTGTACGGTCTCATTGAAGTCGTACCAATCAAACAAAAAAGACCACGGACTAGCGATCCTCAAAAAGTGAAACCTAAACAGGCTTCCTACAAGTTTCATGTAAAAGTTGGCGATTCCAAAGTACGTGTTTGTAAAAAGGCATTTATGAGCATCTACTCTATCAAAAATCATGCCTTATTTCGATTGCAGCATTTAAATTTAAAAGGGACAATTCCCACTGATCAAAGAGGGAAACAAAGTAATCAGAGAATAATATCTGGTGATGTTATTCATGAAATTAAAGAACACATCTTAAGCTTTCCTGTTAAGCAATCGCATTATGATTCCAAGGAGTATCTTTATTTGGCAGCAGATTTAAACATCAAAGCGATGTATAAGCTTTATAAGGAAAAATTTCCAGCATCAACAGTGAAATTATCCTTTTATTATAAAGTTTTTAAAGATCATTTCAACTTGCACTTTGGAAGACCCCAAGTTGATGTCTGCTGTGAATGTGAAAGGCTGAATATAAGGATAAAGTCACCAGACCTAAATGCAACAGCTGTTATGACTACAAAAGCTGAGCTGGCAGTACACAAAAGAAGAGCGAAGAAATTTTATGCCAGTATTAA

>SinsAD4

GTAATTTATGACAACACAAATCTTTAAATTCAAGCTATTTTTATGATATCCAATAAAGTGACAACTAAACATAATGATAAGTGAAAAATACGAGTAGTGGGGGGAGGTCAATGTTCATCATTGGTTGATATATTATATTGATATTAGGTATATACATAACACAAAACATTTGCAGTATCTACCTTTAAAGTGAAGAGTTTCCCGCACCACGGGCAAAAGTACTTCGTGTCGTGATCTGGATGCACCTTGTGTTTGTCATTTTAAGGAGGTAGATATCATTTCTATTTTAAAGCAATGTTGTTTAAGTATTTATATACAACAACTCGTTTCTATTCTGGCTCAGTTGGTAAAGTACGAGCACAAAAATATGTTCTTACAAAATATTTTCAAGGTGAACCCAAGAAAAGCGATTTTAAAATAGTTGAAGAAGAACTTCCGGAATTAAAAGATGGAGAAATATTGACAGAAGCACAATACCTTAGCGTCGATCCATACATGCGTGCTTATATGATAGGATATAAATTACCGACGGATATGATTGGAGGACAAATTGCCAAGATTATAGCCAGTCGTAACAAAAACTTTCCAGTCGGTAAATATGTTACTGCATCATTTGGTTGGCGCACTCACACTGTGTGTAATCCAGAAGAACCTCCCAGTTTAGGCTTTCTGCCTCTGACTCTTGTACCTGAAGTTAAACCGCATCCAGTTTCTCTGGCGTTAGGAGTGCTTGGTATGCCGGGCAATACGGCGTATTTTGGATTGAAAGAGATATGCAGACCTAAAGCAGGAGAAACCATTGCTATAACAGGTGCAGCAGGTGCTGTTGGCTCTCACGTTGGTCAAATTGCTAAAATATTGGGGTGCCGAGTCATAGGATTTGCTGGAACAGATGAGAAGTGTCAGTATTTAAAAGAAATAGGCTTTGATCATGCTTTTAACTATAAAACAGCTGATATTAGAGCAGCTCTCAAGGAAGGTGCACCTAAACGCGTGGATTGTTATTTCGATAATGTGGGTGGAGAAATAAGTTCGACCATTATTAATTTTATGAATAAAAATGGACGAGTGGCTGTTTGTGGATCCATATCTTCGTACAACGATAATATGTTACCAAAAGCAACTATTTTACAACCTGCCTTAGTGTTCAAAGAACTTCAAATAGAAGGATTTTTAGTTAACCGCTGGATTGATCGCTGGGAGGAAGGCGTCAATGCAAATTTAAATTGGCTACATGAAGGAAAGCTACAATATAAAGAAAAAATATATCATGGCTTCGATAACATGGTTGAGGCTTTAGTTGGCATTTTAAGAGGAGAAAACACTGGTAAAGCAGTTGTTAAAGTAAAATAGATTTTTGATAAAATAAACGCATAATAAATATGTCAAAAAAAA

>SinsAD5

ATTGAACGTCGCATAAGACGAATCTAGTCGCACCGGCCCCACTAAGAACACATTAAAACAAAATGGCCCAAGGACTGAAAAACAAAGTCGTCGTCATCACTGGTGGAGCCGTCGGCATCGGTCTCGAGATCGCCGACAAATTCCTGCAGAACTTCGCGAAGGTGGCCGTTCTTCTAGACATCAACGAGGTCCAAGGCGCTGAAGCTGTCAAGACCCTAACCAGCAAACACGGCAGCGGAAAAGCAGTATTCTACAAAGCTGACGTCACTTCAGATTTGGACAAAATCTACAATACTGTAGTAGGCACATACAAGAACGTAGATGTCCTTGTCAACAACGCCGGCATACTCGACGATACTAAAGACAAGTTAACTATCGATATCAACGTCACAGCTCTCATCCTGTGGTCCATGAAGTACTGGGAGCACATGAGGAAGGATAAGGGTGGCAACGGCGGCACCATCATCAACTTGGCTTCGATTTACGGCTTCAGGGTCGACCAGTATCTGCCAATTTACCAGGCTTCGAAGTTCGCAGTTTTCGGCTTCACCAAATCTCTGGGACACGTTTACAATTACAACAGAACCGGTGTGAAGGTAGTAGCAATTTGCCCCGGATTTACCGAGACGAAGTTGACAGCGTCTCCGAAAACGTGGCCCGATCAGCAACTACAGGCAGACTTCACCAAGTTCGTGGCGCAACAAGCTTGGCAAAAGGTCGACGCTGTCGGCAACGCCGCTGTAGAGATTTATGGAAAGCCCAGCGGTACCGCCTGGCTGATCGAAGGTGCCAAACCCATCGTCGAGGTCTGAATTGGTGAGGTAATGTTTCAGAAAGTATTTGTATATAATTGTTGAAGCGGACTGTCTGATTAAAAAAAAAATCTTGTGCCATGACTTGTAATTTGTATTACAAGTCGATATAGTAAACTATTGTACTTTGTTGAAACCGATGGAAACAATTGTCAAATAATTAATATTGAATTATTATTAACTTTACATTCCGTCTCCAGAAACAGTAGTAAGTGTGGTTACCAAATATTACAGGAAAGTCCATTTATGTATGGAATGAATGTAATTAATTATAAAGACTTGAATTTTTCTAATAATTTTTTTTACTCTATATTGAGGCTTTAATGTTTCTTTTAATTACATAATGTTTTTATATAAAATGTAATAGCATTTAATGTATATTTTTTGTATTCACCTA

**AR:**

>SinsAR1

GAAGGCTTTAGCGTCGAGCAGTCAGGTTTCAACTCTTGGACAAGATGGCACGATTCACAGCACCTCTGCTCTTATTAGCATTTTTTAATGCGGCAATGGCGGAAATAGCACCGACCATACAAATGAATGATGGTAACAGAATTCCAATCATTGCACTGGGAACTTATGGCTCAAGCCAAGACATAGCGAGGATGCGCCAAGCTATAAATTGGGCCGTAGAAACTGGGTATAGGCATATAGATACGGCTTCTTTGTATGGGAATGAAATAGAAATCGGTCAAGGAATAGCTGATGTCATTAGGAAAGGTCTTGTGAAGAGAGAAGATTTGTTTGTCACGACAAAGTTGTGGAATGATAGACATGCTCGTAACCAAGTGGTGCCGGCTCTACGGGAGTCCTTGAGAAAATTGGGTCTGGATTACGTGGATTTATATCTTATTCATTTCCCTGATGCTCAAAAGGCTGATGGCTCACCCGATAACATTGATTACCTAGAAACTTGGCAAGGTATGGAAGAAGCTAAAAGGCTCGGTTTGGCCAAATCGATTGGAGTCTCCAACTTTAATATTCAACAAATTGATAGACTGCTTGCTAACAGTCAGATTAAGCCAGCTGTCAATCAAATTGAGGTCCATTCCACTAAAACCCAAGTGCCTCTTGTCACCCATTGTCAGAATCTTGGCATCGTAGTAACAGCATATAGTCCTTTCGGTCATTTAGTCTCGAGGGGCAATCCTAATGCTCCTCCACCCAGGATTGACGACCCGACCCTCGTGCGCATTGCCAACAAGTATGGAAAATCTACCACTCAAGTGGCGCTTCGGTATCTGATCGATCGTGGATTAATACCATT

>SinsAR2

TTTTTATCTATGCTCCAATGATTTTATTGTTACATATTTAATTCTTCTCAAATGGATAGTGAGGCGAATCTATCCATTGTTTCACGGGGATGGTACGATAGTTCCTGTCGTAACTCCTCAGGGTTTGCTTCTCGTCACCGGTCAGCTCGAAGTCGAAGATGTCGATGTTCTGCTCGATGCGGGACCTGGTGATCGATTTCGGTATCGGTATCGCGCCAAGTTCAACAAGATATCTTAAAGTTATCTGCGGCACGGTCTTGTTGTACTTGGTGGCAATGGCCACCAGCGCCGGCTCGTCGACCCTTGGCGGCGGCGCGTCCGGCGTCGCCTTGCTGGGAAACAGCGACCCGAATGGCGTGTAGGCGGTGACGGCGATGCCCTGCTCCTTGCAGTAAGCCAGCAGCGCCGGCTGCTGGAGGTTCAGGTTTATCTCAACTTGCAGCATAGCCGGTTTGACAGAGGTCTGTGATATCAGTCGCTCGATCTGCTGCTGGTTGAAGTTGCAGATCCCAATGGATTTGGCGAGACCCTGATCCTTAGCCTCCACCATGCCTTGCCATGTCTCCACGTAATCCGTGTTGTCTAGACTGCCGTTGGCATAGAAACCAATGGGCCAATGGATGAGATACAGGTCCACGTAGTCCAGGCTCAGTTGCTTGAGAGACTCCTTCAACGTCGGCACTACAGCGTCTTTAGCGTGGCGTGTGTTCCATAGCTTAGTAGTAATGAATACATCTTCCCTTTTCACAACTCCTTCGCTTATTTTCTTGTTCACTGCGCGTCCCACCTGATCCTCGGTAGAATAGATATGTGCAGTATCAATGTGCCTGTAACCCGCGTCGATGGCCCACTCCACTGCTTTCTCTACTTCATCGCCAACTGGATCAACTAGACCTCCTTCCTTAGAGAAGCCAAGCCAGGTTCCAATACCCAAAGTGGGTATTTCTCTTCCGTCGTTTAACTTCACTAGAGGCACCACGCTGGAACTGCGCACCTCATTAGCAAGCAACGCTATCGCCATTACCAAACAATATTTTAACGAATTCATTTCGCTTTTTTTATTTCTATGAAATATGAATATAATTAAACAAAACACCGCCAGCGCCAAAGATTGGCGTCTTTGCA

>SinsAR3

TACTTACGTTTAAACTTGTTTTATGGGGTGCGGTCAACCCGGGCGAAGCCGGGACGAGCCGCTAGTAATAATATATAGATATTATTATAGATATGACCTGAAATTGAACAGCCATCCACCTAGAATAGTCGGTGCCACGTCTAAAAATAAGTAATTTTTTAATAAATGAAGATATTTATAAAACGTATATTAATCTTCTTTGCTATGTTCAATGATGTATTTGATTTCTGCTTCTGTTAAATTCTTTTTCTCGAATGGAAATTCTGGATGATTGTACCATTTAGCTGGGGTTCTGAGTCTATAATTTTTGTTAAAACCTGATAATTCGGCTACTTCATCAGCAGTAAGACTGAAGTCAAAAATATTAATATTTTGTTCTATACGATTTTTATTAGTAGACCGGGGTATAGGAACTAACTGGCGATCAATCAAATATCGAAGTAGTATTTGGGGCACAGTCTTATTATATTTGCCGGCTAAGCTTTGTAAGGTTGGATGGTCGACGCGTGGAGGCGGAGCATCACTCTTGCGGCCAAGTATAGCTCCAAACGGACTATAAGCCATTACAACAACACCATGATTGCGGCACCATTCAATTAGTTCCTTTTGGGTTATCGTAGGGTTTACCTCAATCTGCAAAACTGCAGGCTTTATTTTCGAATTATTCCAGAGTCTTTCCAATTGTGTTAAATTGAAATTGGATACACCTATAGACTTCGCCAGATTCAATTCTTTGGCGTCTTCCATTCCTTTCCATGTTTCAAGATAATCTGTTGAACTAATACTGCCATCTTTCTTGTATGCCATTGGATAATGTACTAAATATAAATCAACATAGTCCGTCTGCAAGTTCTCCAAAGATTTCCTTAAGGCTGGTATCACTTCATCTCTTTCGTGCGCATCATTCCATAATTTGGTAGTAATGTACACTTGTTGTCGTTCGACGCTATTGTCCCTTAGGTAGTTGTGTACTCCCAAGCCTACTTCGTCCTCAGTAAGGTACAGAGCTGCGGTGTCTATATGCTTATATCCGCCCTCCGAAGCCCATTGTACCGCGCGCGCCAACGAGTGGTTCACTGGCAAGATCCGTGTTCCATCAGCTAAGTGTCCCAAGCTAGTGCCCAATGCGACTGCAGGTATTTCGTTACCATCGATCAATTTGTATGTCACTACTTCTGCTCCATTAACCAATCCATTGGTGTATGACAGAAACAATAGAAAATGAGTCAAGCAAGTAGTCGCGTCCATATCTCGCGATATACTACAACAGACTACATACATATGTACTAGTACATGCGAGATATATTGTAAGTATGATTGAGATGGTAGATCATTATACAAATACAGTGTGTGTTACGCCTGACATGATAAATCAATTTTATAATAAGTATAAGTGCAACTAAACAAGATTTATCTTAGTCTTATTTGCGATAATAATAATGTGGAAAAAAGTGCATATACCTAAAGTTTTATTTACGGGTAAAAAGATATGTTAAAAAAATTGATATATTTTCTTCGTTTTATAAATACCAGGGTTTCTTAATCTTATATCCCCATGTATGTAGGTAGCCCTACTGAATTTACAAAAGACTGTTTACGTAACCCTTTATCAGCCGAAAATATAACTATGTATAGAAAAAAATAAGGTTTTCTCTTTATCAATAACACAGAACATAGGTACAGATTACTGGATAAATAAAATAATATAGGTATTATTCATGTGAGGGATGAGTTTGTG

>SinsAR4

GTTGTAATTAATTCTATACGTACCGCGGTATCGACAACATGGCTCTACTTTGGTTTGTGTTAACTATATTTATTGCAAACACTATGTGTGCTGATGAAATTGATGGAAAAGCACCAATAATTCTGCTGAACGACGGTAACGCTATACCTGCCATCGGAATAGGCACATTTTTGGGCTTTGATGAGAACGGACAAAAAGATCCGGCTGAAAATGAAGTTGAGAACGCCGTCAAATGGGCCCTAAACGCAGGATATCGAATGATAGACACGGCCGCGGCTTATCGCAACGAGGACCTTGTGGGACTTGGTATCAGACAGTCAGGAGTTCCGAGAGATAATGTTTTCATTGTAACTAAGTTAGCATCGACTGAACAGAGGCGTGTCATCCCAGCTTTGAGAGACTCCCTTGCAAGGCTGAACACATCATACGTTGATTTATATTTGATACATAATCCTGTAGCTTTTACGCCAGACCACAGCGCGTACGACATAATAGACTATTTGGACACGTGGAAAAGTATGGAAGAAGCCAAGGAATTGGGTCTCGCGAAGTCAATAGGCATATCCAATTTCAATATAAGCCAAATCGAAAGACTCCTGGCCAACTGCAAAGTGAAGCCGTCAGTTTTACAGGTGGAAGTAAATTTAAATCTAGCTCAAAATAATCTCCTGGAATTTTGCAAAAGGAATGATATAGTTGTAATGGCTTATTCACCCTTTGGATCGTTGTTCGACAAAACGGATGTCCCTCCTCCCCCTCGAGCTGACGACTCCACGTTAGTTGCATTCGCTCAAAAATATAAGAAAACTACACCTCAGATTGTATTGAAATATTTGGTACAAAGAGGATTGGTACCAATTCCTAAGTCCGTGCACAAAGAAAGAATTGAAGAAAATATAGATATATTTGATTTCGAGTTGACCCCCGAGGAAATGAAAACTCTCGGTAAATTCAACAAAAATTACCGTGTCGTATGGCCGAATTTTTGGCAAGACCATCCTTATTACCCATTCGAGAGGAAAGATGTCCCCGACCCAGATCCTTTCAAAGGAAAGAAACAAGGAAATTAAAAGCGGTGATGGAGATTAATTGTACTCACGTTTGTGGCATTTATATCAATGATTACATTTCATGCTAAAAACAATAAGGCCGCCTTTTTTCACCATGCTTTTTCATAAGTATTATATTATGTTATTTTTATTTTATGTAAGGTGTGAAATAAAGTATAATATTATTATTATTTTGTTCGCAT

>SinsAR5

GACAGCCATATAAGCTACAGCGTCAAGAAATTGACATTATTTTGTTAGTAGGTACATAATGTTATGCACGTGTAACATTATTTGCACGTGTATGTAATGTACTTATTCATGTTATAAATACATTATTCATGTTATAAAGCTTATACAGGCTTTTATAATATGGCTCTTGAACTGGTTAGTGTGTGGTAGTCGTTCGAACGAACAACATGGCTTCACTCATGATATTATCGGTCTTCGGTTTCGGATTCAATGTGGTGATGGCTACTCAAGCACCAACTGTAGTGTTAAACGATGGAAATAAAATACCAATGGTTGCTTTAGGAACTGGTCGAGGGACAGCAAAAGAGTCCGAATCGGTCGATGAAGTGCGCAAAGCAGTCTTTTGGGCAATCGAAGCTGGGTACAGACACATAGACACGGCTGCTATATATGATGATGAGCCACAGGTGGGCCAAGGAATAGCAGATGCCATCGCTAAAGGCATCGTGAAGAGGGAGGAACTGTTTGTCACTACTAAGTTATGGAACGACAGACATGCCCGTGAAAGAGTTATTCCTAGCTTGAAAGAATCGCTAGCCAGATTAGCTTTGGATTACGTCGACTTATATTTGATTCATTTCCCGATAGCCACAAAGGCTGATGGTTCTCGAGACGATATTGATTACCTTGAAACGTGGCAGGGTATGGAGGAGGCAAAGGAATTGGGGCTCGCAAAATCTATAGGAGTGTCCAACTTTAATTGCACACAAATCGATAGGCTCATAGCTAACAGTAAAATCAAGCCTGCTGTTAATCAGATAGAGGTAAACCCAACGTTAACACAAGAACCATTAGTTTCCCATTGTCAAAAGTTGGGTGTGGTTGTTATGGCATACAGCCCGTTCGGTTTCATGGTGACTAGGAAGCACGACCATACTCCTCCACCCCGAGCTGATGATCCTAATTTAGTCAAGATTGCTGAAAAGTATGGAAAGACGACTGGACAGATAGTTTTGCGGTATTTGATTGACAGAGAATTGATTCCTGTACCAAAATCGACGAACAAAGAGCGAATTGCACAGAACATTGCTTTATTTGAATTCCAACTCACAGCCGATGAAATCGCTAGTATCAATAAATTCAATAAAGACATTAGAGTGATCAATCCTAAAGGCTGGAAGGACTATCCCAACTATCCATTTGAACGAGAGTAAATTGATATTGTAATAAATCAATAAAGTCTGCAGTATTATTAAAAAAAAA

**ATF:**

>SinsATF1

TTTAAACTTTTATGATTTATTGATTTTTCAGTCTACAAAATATAACATTTTTTAAATGTCTATATGTCTGTTTTCAATATTTACAAGAGGTATAAAACTATTACTGAATTATTTTAATAACAATCCTATGTGAAATACATCACAATCAAATAAATGTATACTGTTTTACAGCAAATAAATATTTTATTTTAAAGGCACTGCAGTAAATATATAGATATAGACAAAACTTGAGATGTGATTGAAATAAGTCATTATAATATTATCAAGTCCAATAAAAATAATGTTTATTTGTTTTCACTTACAATTTTTCAATCATGATAGATGATGCTCCCCCACCACCATTACAGATTGAAGCTACACCTTTTTCTCCTTTCTTCAGAGCATGACACAAATGTACTACAATTCGAGCACCAGACATGCCAATAGGATGACCTAGACTTACTGCACCTCCATGTACATTTACTTTAGACGGATCCAACTCTAAAAGTTTCAAATTAGCAAGAGCTACAACACTGAACGCTTCATTAATTTCCCATAGAGCTACATCATCTTTCTTTACACCTGTTTTTTCTAATAATTTAGGAATTGCTACCGCAGGTGCTATAGGAAAATCTATTGGATCACATTCTCCATCAGCATAACCTATGACACGAGCTATAGGTTTCACATTCAATCTTTCTGCTGCATCTGGAGTCATCAATACTAATGCAGCGGCACCATCATTCAGAGTTGAGGCATTCCCTGCAGTTACCGTTCCATTTTCTCTTTGAAATACTGTTGCCAATTTGCCAAATTTTTCAAAATTAACTTTTTTATATTCCTCATCTTCGGCAAAGATTTGGGGTGGAGCTCCTCGCTTTTGTGGCACAGATACAGGGACAATTTCATCTGCGAATACTTTAGATTCGCTGGCTGCGGCACTTCTCTTATAACTAGAAATAGCATATTCATCTTGTTGTTGTCTTGTGATGTTCAATTTCTTTGCTGTGTTCTCAGCACAGTTGCCCATATGAAATTTATTATATACATCAGTGAGACCATCAAATACAATTCCATCAACTAACTGCATACCCCCATATGTAGTTTCACCTCTCTTCAAATAGAATGGCACATTTGACATTGATTCGATGCCCCCAGCTAGTATTACATCTTGAGTTCCCGTCTGAAGCCCTTGTGATGCCAGCATTATAGATTTCATGCCAGAAGAGCAGACTTTATTTACGGTTGTGCATATTGTACTTTTTGGAAGACCTGCAAATATAACTGCCTGCCGTGCCGGAGCTTGTCCTAAATTTGCAGAGCATACATTCCCTATGTAGACCTCCTTAACTTCTTCCTTTGGAATGCCTGCTCTTTCTACAGCTGCTTTAACTGCTACACCGCCCAGCTCGGTCGCCGAAAGACTCGCCAAGCTGCCCCTGAAGGATCCAATAGGAGTCCTCACTGCTGAAGCTATCACAACCTCGTTAAGAGAAACTTTCGACGAAAAGGCCGCCATGGCTTTAAAAAACTTATTTACAGGTTGCATTTTTATGCTAATTATTCTACTTCCTTTTAAGAAAATCATGATTTATGTATTTTATTCCAGAGTACACCCTTATACAAGACACTGTTCAACGCTTCGTTCTTTGTTGAACAAATATAATACTTTATCGCTTTAAATTAGAAAAAGTATGTATAACAAATAACAAACTTATCTTGAGATACAATAGTAACAATTCTTGTAGTATAATAAACTTTAACTCATTGCAATAAAGTTGAAAATAAGAATAGAT

>SinsATF2

ATCATCTATCATGATTGAAAAATTAACACAAGACATAGTTGGACCGCCGGTTCTGACTTTCTATACAAAAGATCCATGTCCTCTATGTGACATTGTTATGGAAGAATTAGAACCATATAAAGATAGAATAATTATAGAAAAAGTTGATATAACTGAGAAAAATAATTTAAGGTGGTTAAGATTGTACAGACACGATATTCCAGTATTATTTCTCAGTGGACAATTCTTATGCATGCATAGACTGGATAAACTATTATTAGAAAGAAGATTACAAGCAATTGAAGAAAAAAAGTAGATAAATACATGTATTATTTATATAAATGATTACTTTCTTCCTCTGGTAATGGCAAATTAAACTTATTTTTAAAGAACATTCAAA

>Actin

GACACTTGTTAGATGCACCGTGCAGTGATGCACTATAGATGCACTTAAATGGCAGTGCGGCACGAGACCGCTGCGCCCTCCTTGTACGGGGCGGCCCCGGTCCTCGTGCCGACCTATAATAGTGGTGCGCGTGCGACGCTCGCTCACTACCGCGTCGCCTCTCACTGCGCATTCAATCTAATATCTGTACATCGGTGAGCGAGTGAACCAAACCTGTGAGCATGTGTGATGACGATGCGGGTGCGCTCGTGGTGGACAACGGTTCCGGCATGTGTAAAGCCGGGTTCGCCGGTGATGATGCACCTCGTGCCGTATTTCCTTCCATAGTGGGACGTCCACGACACCAAGGAGTGATGGTCGGTATGGGTCAAAAAGACTCTTATGTAGGAGACGAGGCTCAGAGTAAGAGGGGTATCCTTACGCTGAAGTACCCTATCGAACATGGAATCATCACCAACTGGGACGACATGGAAAAGATTTGGCATCATACTTTCTATAATGAGTTACGTGTTGCCCCGGAAGAACACCCAATTTTGTTGACAGAAGCTCCGCTCAATCCAAAAGCAAACCGTGAGAAGATGACGCAAATTATGTTCGAGACGTTCAACTGCCCCGCCATGTACGTCGCCATTCAAGCGGTGCTGTCGCTCTACGCATCCGGCCGCACCACCGGCATCGTGCTGGACTCCGGCGACGGCGTGTCACATACGGTGCCAATTTACGAGGGCTACGCGTTGCCGCACGCTATACTTCGACTTGACCTCGCAGGCCGGGATCTCACCGACTATCTCATGAAAATTCTTACAGAACGCGGTTATTCGTTCACGACCACGGCCGAACGAGAAATCGTACGCGATATTAAGGAGAAGCTCTGTTATGTGGCACTCGATTTCGAACAAGAGATGCAAACAGCGGCCGCGTCCACTTCGCTAGAGAAGTCTTACGAATTGCCCGATGGCCAGGTGATCACTATAGGCAACGAAAGGTTCCGCTGCCCTGAAGCCTTATTTCAGCCATCGTTTCTGGGCATGGAATCCTGCGGCATCCACGAGACCGTGTATAACTCGATCATGAAATGCGACGTCGATATCCGTAAGGATTTATATGCTAATACGGTGCTCTCGGGCGGCACTACCATGTACCCAGGTATCGCTGATCGTATGCAAAAAGAGATCACGGCGCTGGCTCCATCGACCATCAAAATTAAGATCATTGCACCCCCCGAAAGAAAGTATTCAGTGTGGATCGGTGGTTCTATCCTAGCGTCACTTTCAACTTTCCAGCAGATGTGGATTTCAAAGCAAGAATACGATGAATCGGGTCCCGGTATCGTACATCGCAAATGTTTCTGAGCAGCTTGCACTTTTGCTTCTTAATAGCTACATTGCTTCGCACGCGCATCTCGCTAGTACACCACCCATGTTAACATATTTTTTATACTTTGCATAGAACACTGTGCTTTGTTGCATCGTGTCCTGAGTTTTATTTAAGATCAATTGTTTCGAATACACTTTTTGTTATTAATAAAAAA
